# Filovirus reactive antibodies in wildlife hunting communities suggest ongoing zoonotic spillover in forested Guinea

**DOI:** 10.1038/s41467-026-77653-3

**Published:** 2026-10-08

**Authors:** Grace Hood, Joseph Akoi Bore, Tom Tipton, Zézé Onivogui, Koly Sovogui, Kpadè Zézé Zoumanigui, Joseph Timothy, McKenna Roe, Lianying Yan, Simon Scott, Nigel Temperton, Edward Wright, Gwenda Hughes, Sanaba Boumbaly, Eric D. Laing, N’Faly Magassouba, Kimberly Fornace, Miles Carroll

**Affiliations:** 1 Pandemic Sciences Institute and Centre for Human Genetics, University of Oxford, Oxford, United Kingdom; 2 Centre de Recherche et d’Analyse Biomédicale (CRAM), Macenta, Republic of Guinea; 3 University of Sonfonia, Conakry, Republic of Guinea; 4 London School of Hygiene & Tropical Medicine, London, UK; 5 Department of Microbiology and Immunology, Uniformed Services University, Bethesda, MD USA; 6 Henry M. Jackson Foundation for the Advancement of Military Medicine, Inc., Rockville, MD USA; 7 Viral Pseudotype Unit, University of Kent and Greenwich, London, UK; 8 School of Life Sciences, University of Sussex, Falmer, UK; 9 United Kingdom Public Health Rapid Support Team, London, UK; 10 Le Centre de Recherche en Virologie, Laboratoire des Fièvres Hémorragiques Virales de Guinée (CRV-LFHVG), Conakry, Republic of Guinea; 11 Saw Swee Hock School of Public Health and National University Health System, National University Singapore, Singapore, Singapore; 12 Centre for Nature-based Climate Solutions, National University of Singapore, Singapore, Singapore

**Keywords:** Viral epidemiology, Ebola virus, Ecological epidemiology, Forest ecology

## Abstract

Forested Guinea has been the site of multiple high-consequence viral spillover events, including the index case of the 2013-16 West African Ebola epidemic. Deforestation and wildlife hunting rapidly expand the human-animal interface, increasing opportunities for transmission. Here we integrate serologic, spatial and questionnaire data to characterize zoonotic filovirus spillover events and risk factors amongst wildlife hunter communities in Forested Guinea. Hunters and close contacts from 43 villages in 2017 (*n* = 511) and 2023 (*n* = 648) were screened using a multiplex microsphere-based immunoassay panel of 9 filoviruses, identifying IgG binding against Ebola virus (EBOV) in 57/1159 individuals (4.9%), as well as species-specific binding against Bundibugyo (BDBV) (49/1159, 4.2%), Taï Forest (TAFV) (13/1159, 1.1%), Reston (4/1159, 0.3%), Měnglà (MLAV) (8/1159, 0.7%), Lloviu (LLOV) (8/1159, 0.7%) and Bombali (BOMV) (1/1159, 0.1%) viruses. Parallel pseudotyped virus neutralization assays identified species-specific neutralization of EBOV, BDBV and TAFV, as well as likely cross-neutralization of BOMV, LLOV and MLAV, suggesting multiple zoonotic virus spillover events. Mixed-effects regression analyses of satellite-derived land-cover data identified environmental risk factors (including forest fragmentation) reflecting potential pathways of exposure. Together, our data suggest multiple ongoing zoonotic filovirus spillover events in wildlife hunting communities and highlight the importance of landscape configuration in driving pathogen spillover.

## Abstract (web summary)

Risk of virus spillover is heightened in areas where the human-wildlife interface is expanding, such as Forested Guinea. Here, the authors present results from a serological survey to characterize filovirus exposure in wildlife hunting communities.

## Introduction

Forested Guinea, Republic of Guinea, has been a hotspot for high-consequence viral spillover, including the 2013–16 West African Ebola epidemic’s index case, and West Africa’s first Marburg virus (MARV) case in 2021, highlighting the ongoing threat zoonotic filovirus spillover poses to the health and economic security of the region^1,2^. This historically forested and mountainous region shares porous borders with Sierra Leone, Liberia and Côte d’Ivoire. The majority of the population is engaged in rural subsistence farming, with 66.9% of the population living below the poverty line. Healthcare access is limited, with World Bank estimates of 0.2 medical doctors per 10,000 people falling far below the global average of 17.2 per 10,000. Climate change, political instability and multiple refugee crises from neighboring civil wars further confound infectious disease surveillance, particularly at the extensive human-wildlife interface, which is progressively expanded by deforestation^3^. Land-use changes, such as the anthropogenic conversion of natural habitats for agricultural or urban use, drive viral spillover by fundamentally disturbing reservoir species’ ecology, altering their distribution, behavior and physiology, simultaneously increasing both human interaction and pathogen shedding^4–6^. Further, evidence shows human-disturbed landscapes systematically select for species traits correlated with host status, driving a net increase in zoonotic host diversity^7^. Analyses of land cover surrounding Ebola virus spillover events have suggested associations with deforestation, highlighting the need for further investigation^8,9^.

More immediate drivers of spillover, such as wildlife hunting, have also been implicated. Historical outbreak investigations have identified epidemiological links between individual cases and exposure to susceptible host species^1,2,10–15^, while large-scale seroprevalence studies have uncovered significant population-level associations between viral exposure and wildlife hunting^16^. In the case of filoviruses, bats are often highlighted as the known reservoirs of Marburg virus and Ravn virus^17,18^, and suspected reservoirs of other orthoebolaviruses, based on a growing body of molecular, serological and ecological evidence^19,20^. This evidence includes the detection of Ebola virus (EBOV) RNA and antibodies in multiple insectivorous and fruit bat species in the Democratic Republic of Congo and Gabon^19,21^; the recovery of the complete Bombali virus genome from insectivorous bats in Sierra-Leone^22^ and RNA in Guinea^23^, Kenya^24^, Mozambique^25^ and the presence of anti-Sudan virus (SUDV) and anti-Reston virus (RESTV) antibodies from multiple bat species across Asia and Africa^26–28^. Similarly, Lloviu virus (LLOV) has been repeatedly isolated from Schreiber’s bats in Hungary and Italy^29–31^ and the complete genome for Měnglà virus (MLAV) has been recovered from Rousettus bats in China^32^. Other animal species, including nonhuman primates^33–36^ and duikers (*Cephalophus* spp.)^37^, are known to be susceptible to orthoebolaviruses, and may serve as intermediate hosts, as in the case of the only documented human case of Taï Forest virus (TAFV), who was infected in Cote d’Ivoire while performing a necropsy on a wild chimpanzee^38,39^.

The severe pathogenicity of many filoviruses has been established (with EBOV, MARV, Sudan (SUDV), and Bundibugyo (BDBV) viruses causing human outbreaks with case fatality rates ranging from 25% to 90%), while others may cause milder (TAFV) or even asymptomatic human infection (RESTV)^40^. For others, including BOMV, LLOV, MLAV or novel species, the human infective and pathogenic potential remains unknown, highlighting the urgent need for ongoing surveillance. Multipathogen seroepidemiological methods provide great utility in the study and surveillance of infrequent spillover events at the human-animal interface, particularly for pathogens of variable clinical presentation and reliably sustained, measurable immunoglobulin-G (IgG) persistence, as is documented for EBOV^1,41–45^.

Previous serosurveillance of wildlife hunting communities in Forested Guinea in 2017 identified distinct immune signatures to multiple EBOV antigens (GP, NP, VP40) by ELISA or western blot, which were non-neutralizing and thus not plausibly explained by prior EBOV-Makona exposure, suggesting exposure to other antigenically related non-EBOV filoviruses^46^. In this study, we return to Forested Guinea to resample and expand the wildlife hunter study population 6 years later, in order to characterize these serological signatures in a multiplex bead-based immunoassay screening for binding antibodies against 9 filovirus glycoprotein antigens (EBOV, BDBV, TAFV, BOMV, SUDV, RESTV, LLOV, MLAV, RAVV) followed by parallel pseudotyped neutralization assays to disentangle species-specific from cross-reactive neutralizing antibody responses. These data suggest the underreported circulation of multiple zoonotic filoviruses, including EBOV, BDBV and TAFV, demonstrating both persistent antibody responses and ongoing seroconversion and highlighting risk factors associated with putative zoonotic transmission, including environmental land-use changes and hunting of wildlife species (nonhuman primates, bats).

## Results

### Study sites and study populations

Study sites were purposively sampled to include villages that were affected (*n* = 16) and unaffected (*n* = 28) by the 2013–2016 West African Ebola epidemic, as reported in national databases from the Macenta Prefectural Health Direction office. Official designations were confirmed by key informant interviews with village leaders and healthcare workers. Within villages, families known to practice wildlife hunting were approached, and the senior hunter and their spouse (or next closest relative) were invited to participate. Participants <18 years of age, pregnant, with comorbidities, and visitors to the region were excluded. Demographic data, including age, sex, occupation, and data on wildlife hunting practices, were collected by researchers via questionnaires administered in French and Toma (a regional indigenous language).

A total of 511 participants were recruited from 40 villages in 2017 (as previously published^46^), and 648 participants were recruited from 43 villages in 2023 (Table 1). Of the 2017 study population, 52% (268/511) were resampled as part of the follow-up 6 years later. Wildlife hunters (all male) constituted 56% of the study population, with the remaining 45% reporting close household contact with a hunter. Farming was the predominant occupation in 2017 (93%, 476/511) and 2023 (86%, 556/648), however some healthcare workers were also enrolled (3% and 12% in 2017 and 2023 respectively).

**Table 1:** Demographic characteristics of participants in the Guinean wildlife hunting community, 2017 and 2023

Characteristic	2017 n/N (%) *	2023 n/N (%)
**Total no. of participants**	511	648
**Sex**		
Male	288 (56)	407 (63)
Female	223 (44)	241 (37)
**Age, group by years**		
18–30	144 (28)	143 (22)
30–40	120 (24)	154 (24)
40–50	133 (26)	156 (24)
50–60	75 (15)	107 (17)
>60	39 (8)	88 (14)
**Age, years**		
Median (IQR)	39 (28-48)	40 (30-51)
Mean (Range)	39 (18-90)	42 (18-110)
**Wildlife hunting**		
Hunter	278 (54)	341 (53)
Non-hunter	232 (45)	306 (47)
Unknown	1 ( < 1)	1 ( < 1)
**Primary occupation**		
Farmer	476 (93)	556 (86)
Healthcare worker	17 (3)	76 (12)
Tradesperson	1 ( < 1)	8 (1)
Other	16 (3)	4 (1)
Student	1 ( < 1)	4 (1)

Data are presented as n/N (%) unless otherwise indicated, where n is the number of participants with the characteristic, and N is the total number of participants included in the respective survey year. Age is summarized as median (IQR) and mean (range). Percentages may not total exactly 100% because of rounding. * Previously published^46^. Abbreviations: IQR, interquartile range.

Serum samples (*n* = 63) collected in 2020 from healthy blood donors, aged 17–80 years, from the United Kingdom were utilized as a presumed negative, non-endemic comparative study population^47^.

Serum samples (*n* = 3) were collected between 2015 and 2017 from Guinean PCR-confirmed Ebola virus disease (EVD) survivors 3–14 months post-infection and pooled for use as a positive control for Ebola virus. Serum samples (*n* = 15) were also collected from urban settings (Coyah and Guéckédou, Guinea) in 2015–2017 from individuals with no known epidemiological contact with EVD, no known case history, and who were negative by EBOV-GP ELISA^41^.

### Wildlife hunting and bat exposure in Forested Guinea

In 2023, a detailed questionnaire on wildlife hunting and other activities was administered to all participants in Toma and French. Consumption of wildlife or “bushmeat” was ubiquitous, with 90% of respondents (581/648) consuming wildlife meat, and 94% (583/619) helping handle, wash or prepare wildlife carcasses prior to cooking. Wildlife was consumed a median of 7 [IQR: 4–15] days per month, and amongst hunters, hunting was practiced a median of 6 [IQR: 3–10] days per month. Wildlife hunting generally was not considered to be seasonal by the majority of respondents (93%, 576/619), with the notable exception of bat hunting. Bat exposure and hunting were reported to be highly seasonal, with 87% (292/334) of respondents seeing them or hunting them (92%, 122/133) at the start of the dry season. Agouti was the most commonly reported food source, followed sequentially by rodent, antelope, boar, monkey, pangolin and bat. Bat hunting was found to be a relatively specialized practice with only 32% (207/648) of individuals either hunting bats or living with a bat hunter. Bats are typically hunted in the forest, in trees, or in the village, and rarely in caves.

Bat contact was more frequent and was typically reported as 2-3 days per week (46%, 296/648), taking place around the village (45%, 259/574) or in the house (28%,158/574) or the forest (14%, 80/574) between 12–6 am and in the afternoon. Forest activities included hunting or collecting wood, plants or food. Cave usage was uncommon (25%, 139/546) and comprised short visits only, with an average time of 13 min [min–max; 0–60] spent inside per visit. Caves were typically used by both hunters and non-hunters. Guano harvesting was rarely reported (0.3%, 2/648).

### Filovirus antigen reactivity in Guinean wildlife hunting communities by multiplex microsphere-based immunoassay screening

**Fig. 1:**
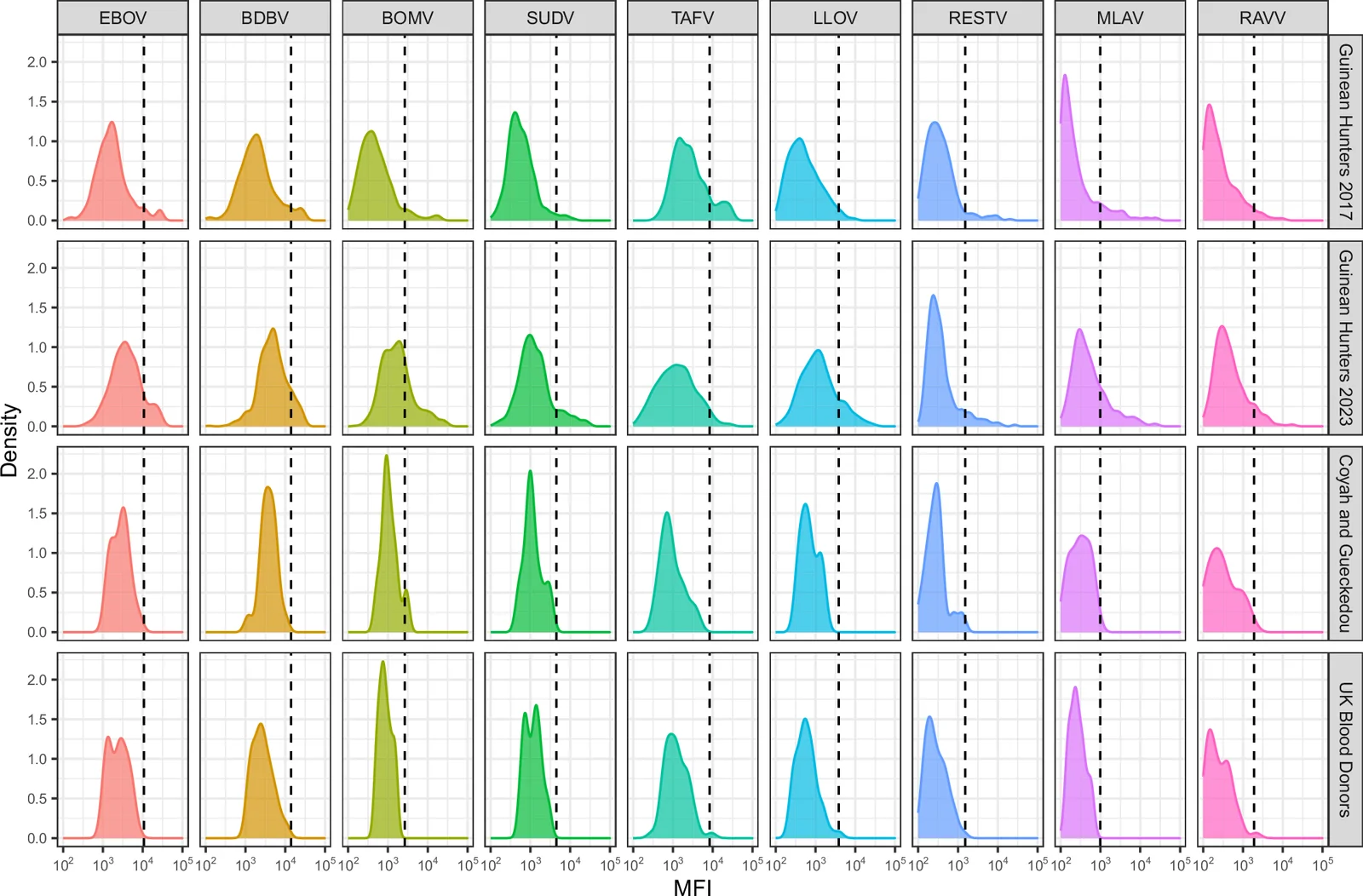
Probability density plots of anti-filovirus glycoprotein IgG distributions among Guinean wildlife hunting communities in 2017 (*n* = 511) and 2023 (*n* = 648), Guinean Coyah controls (*n* = 18) and the United Kingdom (UK) blood donor study populations (*n* = 63). IgG responses were measured by multiplex microsphere-based immunoassay (MMIA) in background-subtracted median fluorescence intensity (MFI-bg) on the Luminex platform. Dashed vertical lines mark seropositivity cutoffs based on the presumed unexposed non-endemic study population (UK blood donor) by calculating five standard deviations above the background-subtracted (MFI-bg) mean. Abbreviations: BDBV Bundibugyo virus; BOMV Bombali virus; EBOV Ebola virus; IgG immunoglobulin G; LLOV Lloviu virus; MLAV Měnglà virus; RAVV Ravn virus; RESTV Reston virus; SUDV Sudan virus; TAFV Taï Forest virus; UK United Kingdom.

Population-level differences in the distributions of IgG antibodies against multiple zoonotic filoviruses were detected between the Forested Guinea wildlife hunting communities (2017 and 2023) and the Ebola-negative Coyah and Guéckédou (*n* = 18) and United Kingdom (UK) control study populations (*n* = 63) (Fig. 1). Initial seropositivity cut-offs were based on the presumed unexposed non-endemic study population (UK blood donors) by calculating five standard deviations above the background-subtracted (MFI-bg) mean. As seen in Fig. 1, a high proportion of the wildlife hunting population had responses above the seropositivity threshold for not only EBOV and closely related orthoebolaviruses, such as BDBV and TAFV, but also more distant filoviruses, including BOMV, LLOV, RESTV and MLAV.

### Classification of immune responses by *K*-means clustering reveals EBOV and non-EBOV filovirus reactivity in wildlife hunting communities, 2017 and 2023

**Fig. 2:**
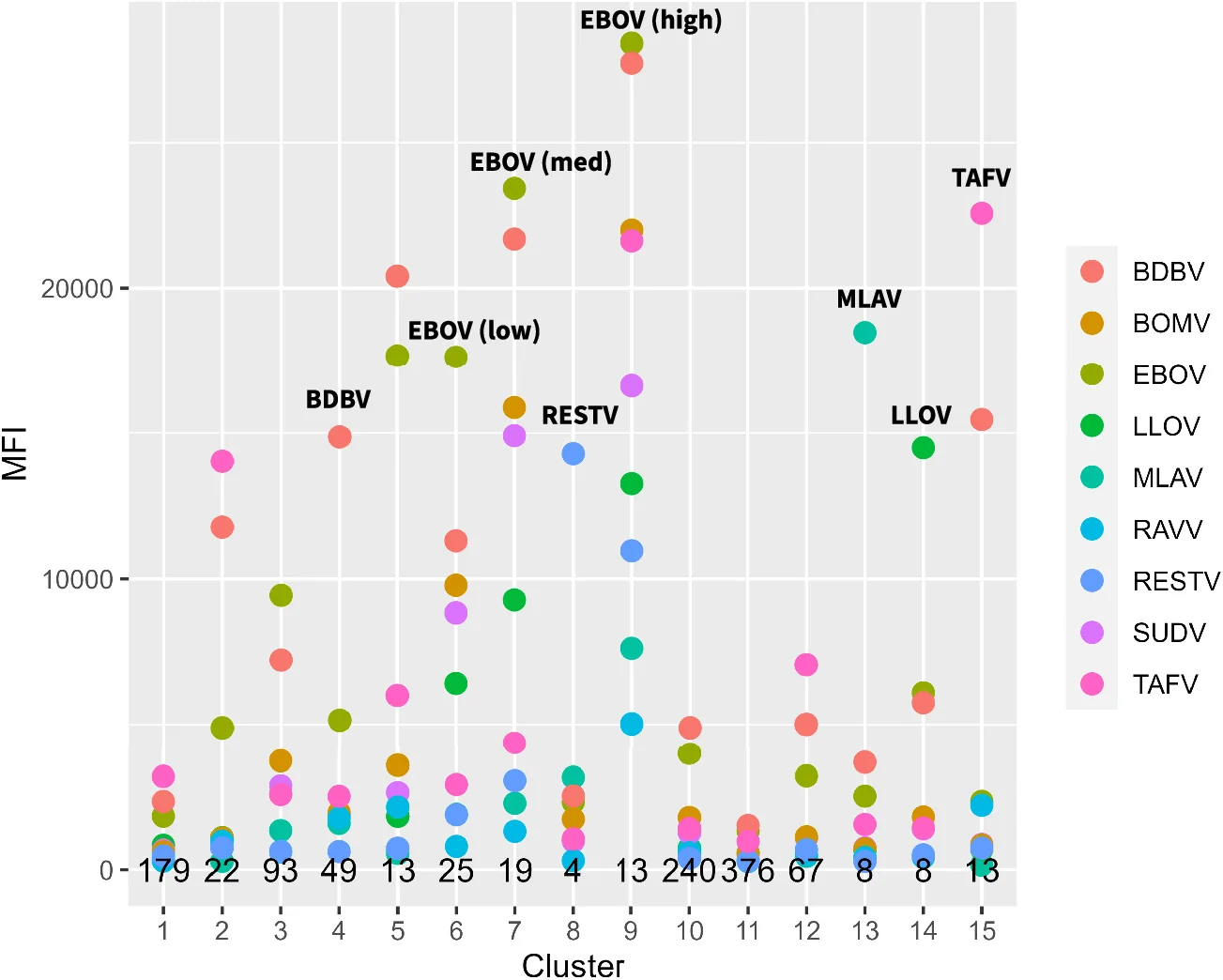
The *K*-means clustering algorithm partitioned anti-filovirus glycoprotein (GP) antibody responses from 2017 and 2023 into *k* = 15 clusters with qualitatively different serological responses. The mean reactivity for each filovirus antigen by cluster, and the number of samples in each cluster, is shown. IgG responses were measured by multiplex microsphere-based immunoassay (MMIA) in median fluorescence intensity (MFI) units on the Luminex platform. Several sets of clusters showed similar ordinal patterns of antibody reactivity across filovirus GPs. Specifically, clusters 2, 12, and 15 shared an ordinal pattern in which average MFI values were highest for TAFV, followed by BDBV and EBOV. Clusters 3, 6, 7, and 9 exhibited a pattern in which average MFI values were highest for EBOV, followed by BDBV and BOMV. Clusters 4 and 5 exhibited a pattern in which average MFI values were highest for BDBV, followed by EBOV. Individual responses of wildlife hunters within these clusters are presented in the Supplementary to show the intra-cluster variation as a qualitative check on whether these clusters are distinct. Abbreviations: BDBV Bundibugyo virus; BOMV Bombali virus; EBOV Ebola virus; GP glycoprotein; IgG immunoglobulin G; LLOV Lloviu virus; MLAV Měnglà virus; RAVV Ravn virus; RESTV Reston virus; SUDV Sudan virus; TAFV Taï Forest virus.

Given the heterotypic cross-reactivity seen between antigenically related viruses, we utilized an unsupervised machine learning algorithm (*K*-means clustering) to classify the IgG binding antibody responses of individuals against multiple filoviruses into “clusters”, or unique exposure profiles. The *K*-means clustering algorithm revealed a stable underlying data structure and was used to group individuals with similar serological responses together into *k* = 15 clusters (Fig. 2). This approach to determining seropositivity allowed us to avoid the use of a serological threshold for each virus, thus minimizing the misclassification of cross-reactive individuals as true positives and identifying groups of individuals with common exposures across multiple viruses.

**Fig. 3:**
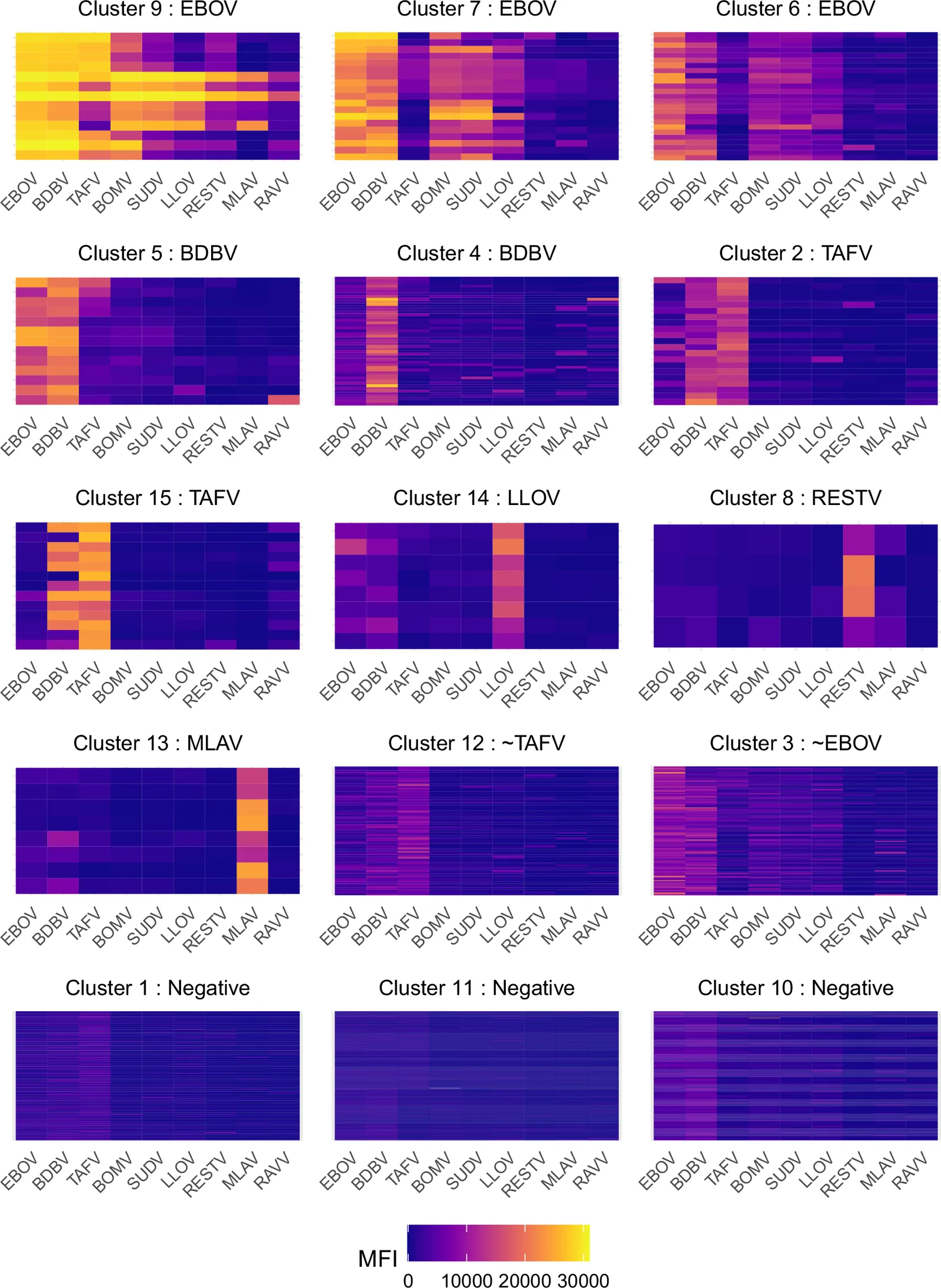
Anti-filovirus glycoprotein (GP) antibody responses of individuals clustered by *K*-means clustering (*k* = 15), Guinean wildlife hunters, 2017 and 2023 (*n* = 1159). IgG responses measured by multiplex microsphere-based immunoassay (MMIA) in median fluorescence units (MFI) on the Luminex platform. Abbreviations: BDBV Bundibugyo virus; BOMV Bombali virus; EBOV Ebola virus; GP glycoprotein; IgG immunoglobulin G; LLOV Lloviu virus; MLAV Měnglà virus; RAVV Ravn virus; RESTV Reston virus; SUDV Sudan virus; TAFV Taï Forest virus.

Three clusters with a total of 57 individuals (57/1159, 4.9%, Clusters 6, 7, 9) were characterized by high anti-EBOV binding, and heterotypic cross-reactivity against related filoviruses, which decreased with phylogenetic distance (Fig. 3) and were considered consistent with prior EBOV exposure, based on their similarity to binding patterns observed in PCR-confirmed EBOV survivors 12 and 24 months post-infection^48^. Interestingly, clusters were also identified with more species-specific antibody binding against singular filoviruses, often in the absence of anti-EBOV antibodies. This included clusters of individuals with antibody binding against BDBV (*n* = 49, 4.2%, Cluster 4), TAFV (*n* = 13, 1.1%, Cluster 15), RESTV (*n* = 4, 0.3%, Cluster 8), MLAV (*n* = 8, 0.7%, Cluster 13), LLOV (*n* = 8, 0.7%, Cluster 14) and one individual with binding against BOMV (*n* = 1, 0.1%) (Fig. 3). Individuals within these clusters were subsequently prioritized for functional assessment and further differentiation by parallel pseudotyped virus neutralization assays (PVNA).

### Filovirus neutralizing activity in Guinean wildlife hunting communities

Individuals with antibody profiles consistent with exposure to BDBV, TAFV, BOMV, RESTV and LLOV were then assessed for their ability to neutralize the filovirus in parallel with Ebola virus-Makona PV. Neutralizing ability was tested by a lentivirus-backbone filovirus glycoprotein pseudotyped virus (PV) neutralization assay. A pool of PCR-confirmed EBOV-Makona survivors from 2014 was included for comparison. Negative controls included an EBOV-negative Guinean pool from Coyah and Guéckédou (*n* = 3) and a United Kingdom blood donor.

**Fig. 4:**
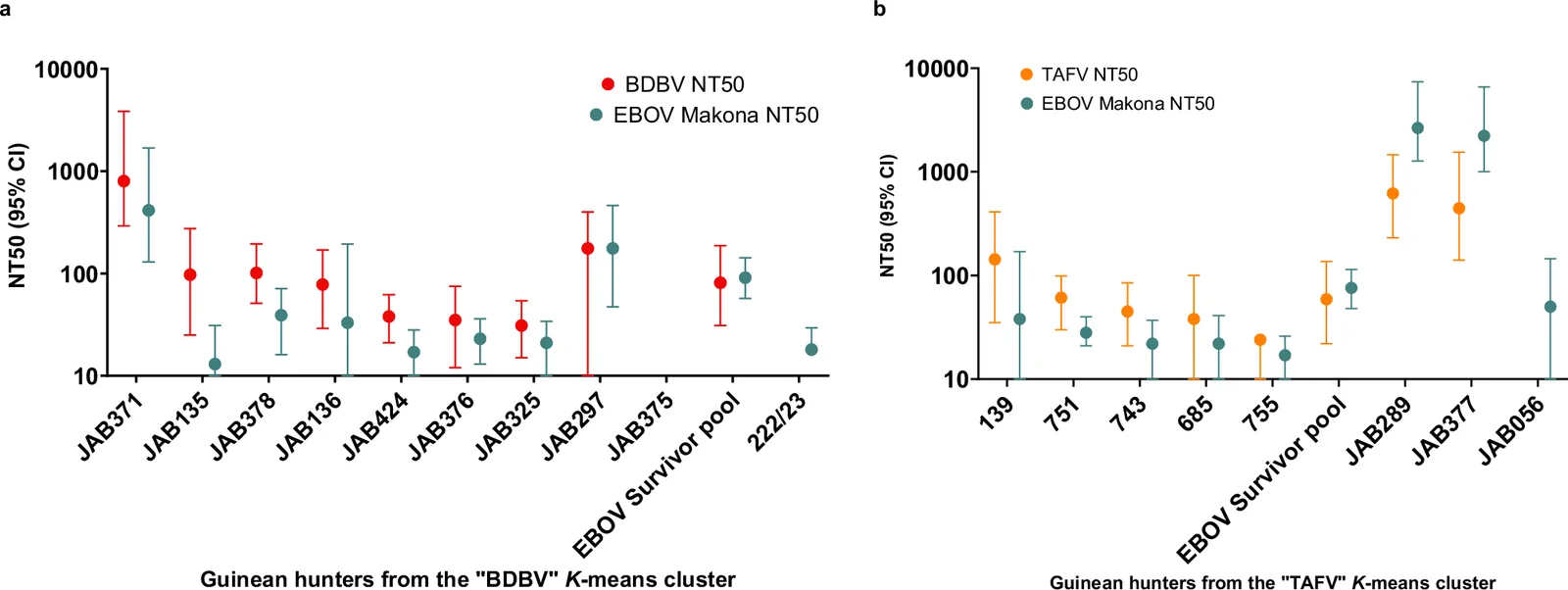
Neutralizing ability of individual sera samples from the Guinean wildlife hunting community against. **a** Bundibugyo (BDBV) and Ebola (EBOV) viruses. **b** Taï Forest virus (TAFV) and EBOV viruses, as tested in a lentiviral backbone pseudotyped virus neutralization assay. Sera were identified by *K*-means clustering as showing the highest specific binding antibody responses against **a** BDBV **b** TAFV. NT50 values (displayed as points, unless NT50 = 0) and 95% confidence intervals (displayed as whiskers, unless >0) were calculated using nonlinear regression in GraphPad Prism 10.0.3. Neutralization assays were performed in technical triplicate for each independent serum sample. The EBOV survivor pool consists of sera from *n* = 3 PCR-confirmed EBOV survivors 3–14 months post-infection. Negative controls included sera from an EBOV-negative Guinean pool (*n* = 3) and a UK blood donor: neither showed neutralization. Additional demographic information is available in the Supplementary Information. Abbreviations: BDBV Bundibugyo virus; CI confidence interval; EBOV Ebola virus; IgG immunoglobulin G; NT50 50% neutralization titer; PCR polymerase chain reaction.

Of those tested from the BDBV cluster, 5/10 individuals had lower NT50 estimates against BDBV than EBOV (though 95% confidence intervals (CI) overlapped), and neutralized BDBV more strongly than the EBOV survivor pool, and all came from two villages which reported no cases of EBOV (Fig. 4). Two of these individuals were household contacts (JAB135, JAB136), also from an EBOV-unaffected village. Persistent responses were shown by those resampled in 2023 (JAB297, JAB376). The remaining samples (including the EBOV survivor pool) showed stronger neutralization of EBOV, with some neutralization of BDBV (Fig. 4).

Of the individuals tested from the TAFV cluster, 5/8 individuals from 2023 had lower NT50 estimates against TAFV than EBOV (with some overlap of 95% CIs) (Fig. 4). Of the LLOV cluster, 1/9 individuals had stronger neutralizing ability against LLOV than EBOV. All samples from the BOMV and RESTV cluster neutralized EBOV more strongly than BOMV or RESTV, though all showed some neutralizing activity against both. In all neutralization assays, the PCR-confirmed EBOV survivor pool showed strong neutralization responses against EBOV-Makona (NT50 90, 95% CI: 57–142), and also demonstrated a lower level of cross-neutralization of BDBV, RESTV, TAFV and LLOV.

### Durable EBOV antibody responses, 6 years post- 2013-16 West African EBOV epidemic

**Fig. 5:**
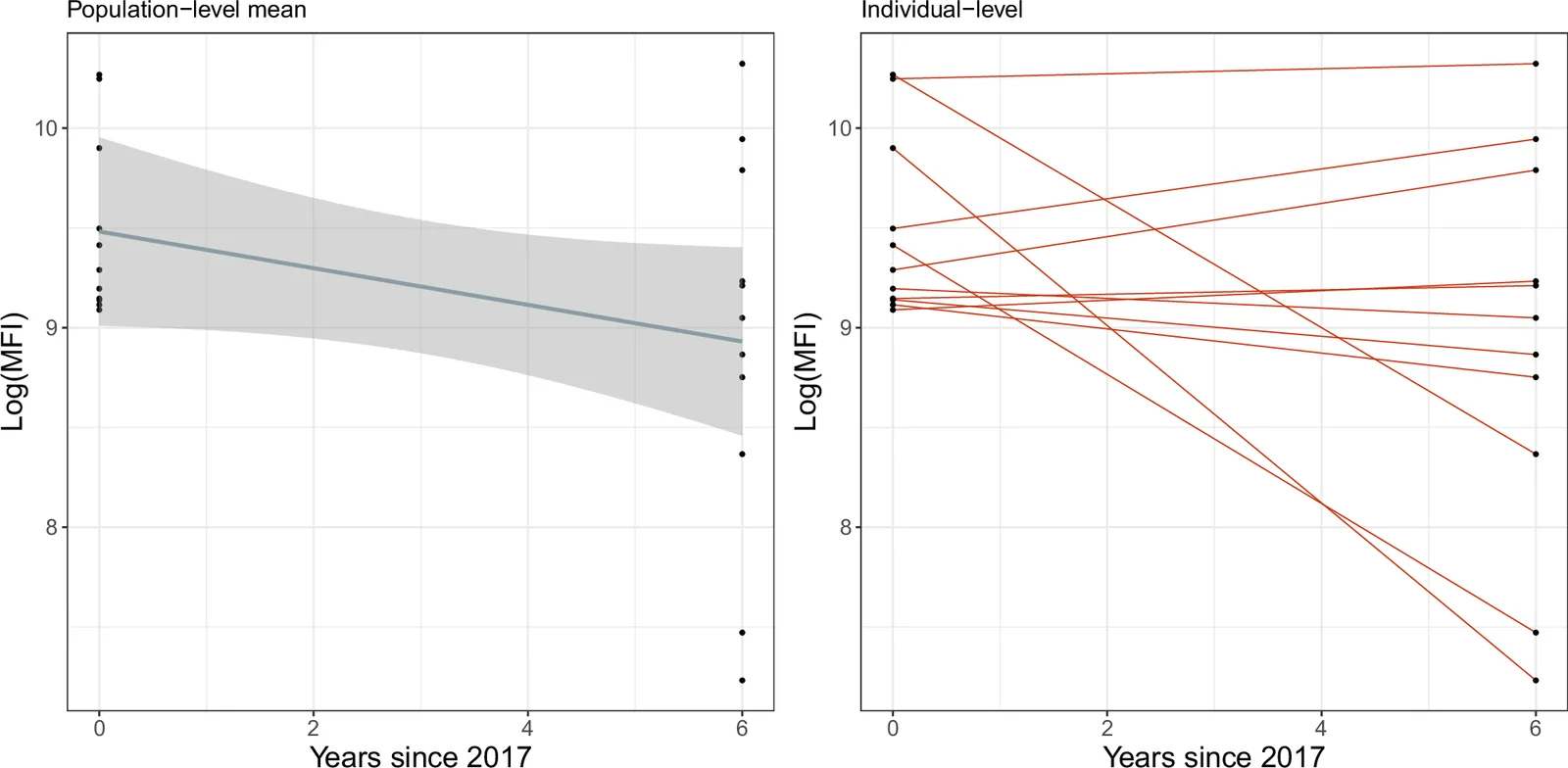
Population-level mean (regression line) with 95% confidence interval (shaded) and individual-level antibody decay models for Ebola virus (EBOV). IgG responses were measured by multiplex microsphere-based immunoassay (MMIA) in background-subtracted median fluorescence units (MFI). Antibody decay rates were modeled as an exponential decrease from time of infection, with a regression model fitted to IgG antibody data from each individual above a seropositivity threshold for EBOV in 2017 who was resampled in 2023 (*n* = 11). Abbreviations: IgG immunoglobulin G.

For individuals with detectable EBOV antibodies in 2017, a comparison of 2017–2023 demonstrated the population-level durability of anti-EBOV antibodies across all villages, including in villages that were not reported as affected by the 2013–16 EBOV epidemic. Antibody decay rates were modeled as an exponential decrease from time of infection, with a regression model fitted to IgG antibody data from each individual above a seropositivity threshold for EBOV in 2017 who was resampled in 2023 (*n* = 11). Based on the population average of these models, the EBOV IgG antibody half-life was estimated as 7.5 years (Fig. 5), though the possibility of re-infection and subsequent antibody boosting could not be excluded.

### Village-level hotspots identified for BDBV in locations unaffected by the 2013–2016 West African EBOV epidemic

**Fig. 6:**
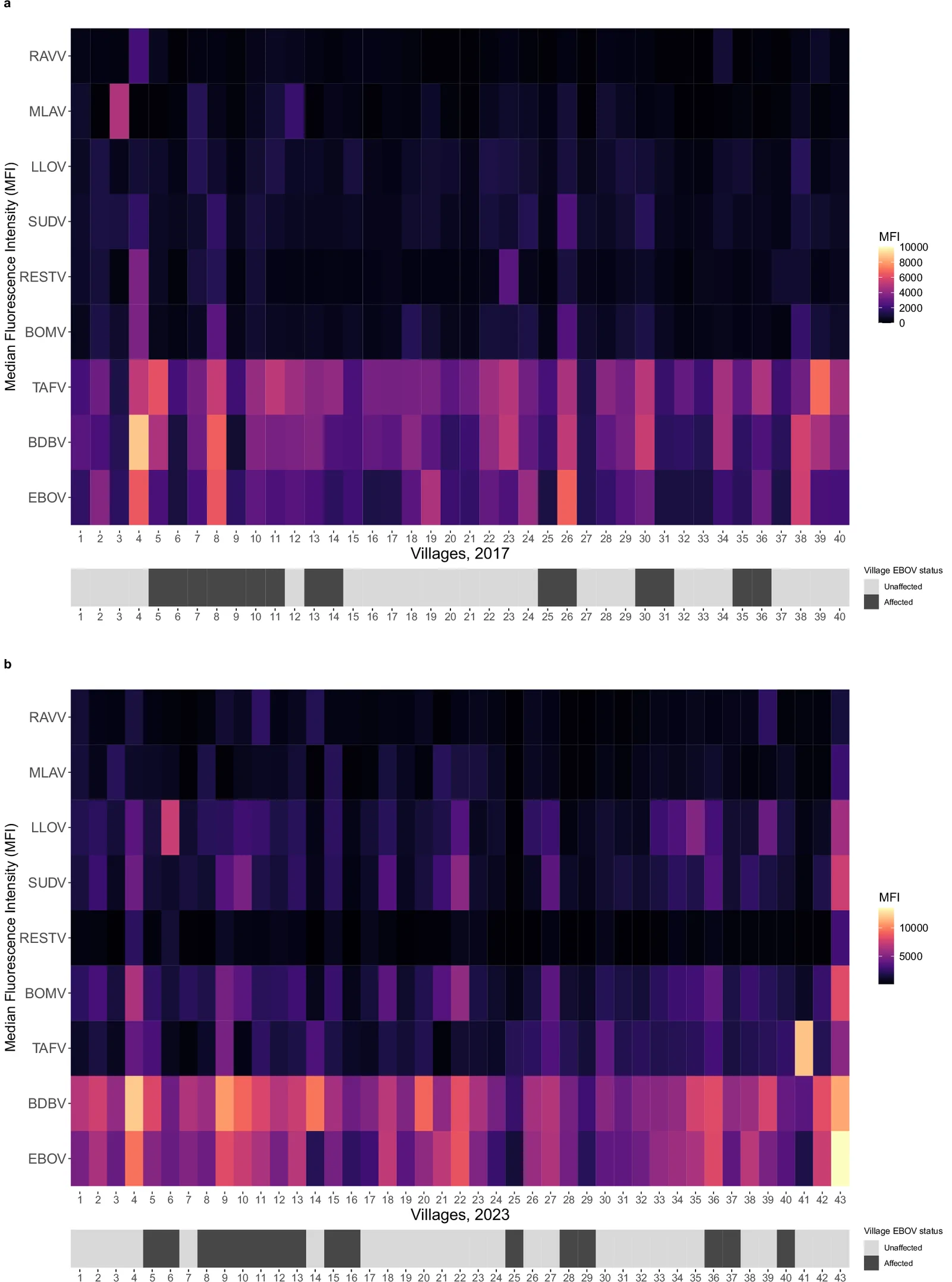
Mean anti-filovirus IgG responses amongst Guinean wildlife hunting communities in 2017 and 2023, stratified by village. IgG responses were measured by multiplex microsphere-based immunoassay (MMIA) in background-subtracted median fluorescence intensity (MFI). Villages (*n* = 40 in 2017, *n* = 43 in 2023) were determined to be affected or unaffected by the 2013–16 West African Ebola virus epidemic based on official Macenta Ministry of Health designations followed by key informant interviews of healthcare workers and village leaders. Villages are numbered from 1–43 by geographic proximity. Abbreviations: BDBV Bundibugyo virus; BOMV Bombali virus; EBOV Ebola virus; IgG immunoglobulin G; LLOV Lloviu virus; MLAV Měnglà virus; RAVV Ravn virus; RESTV Reston virus; SUDV Sudan virus; TAFV Taï Forest virus.

**Fig. 7:**
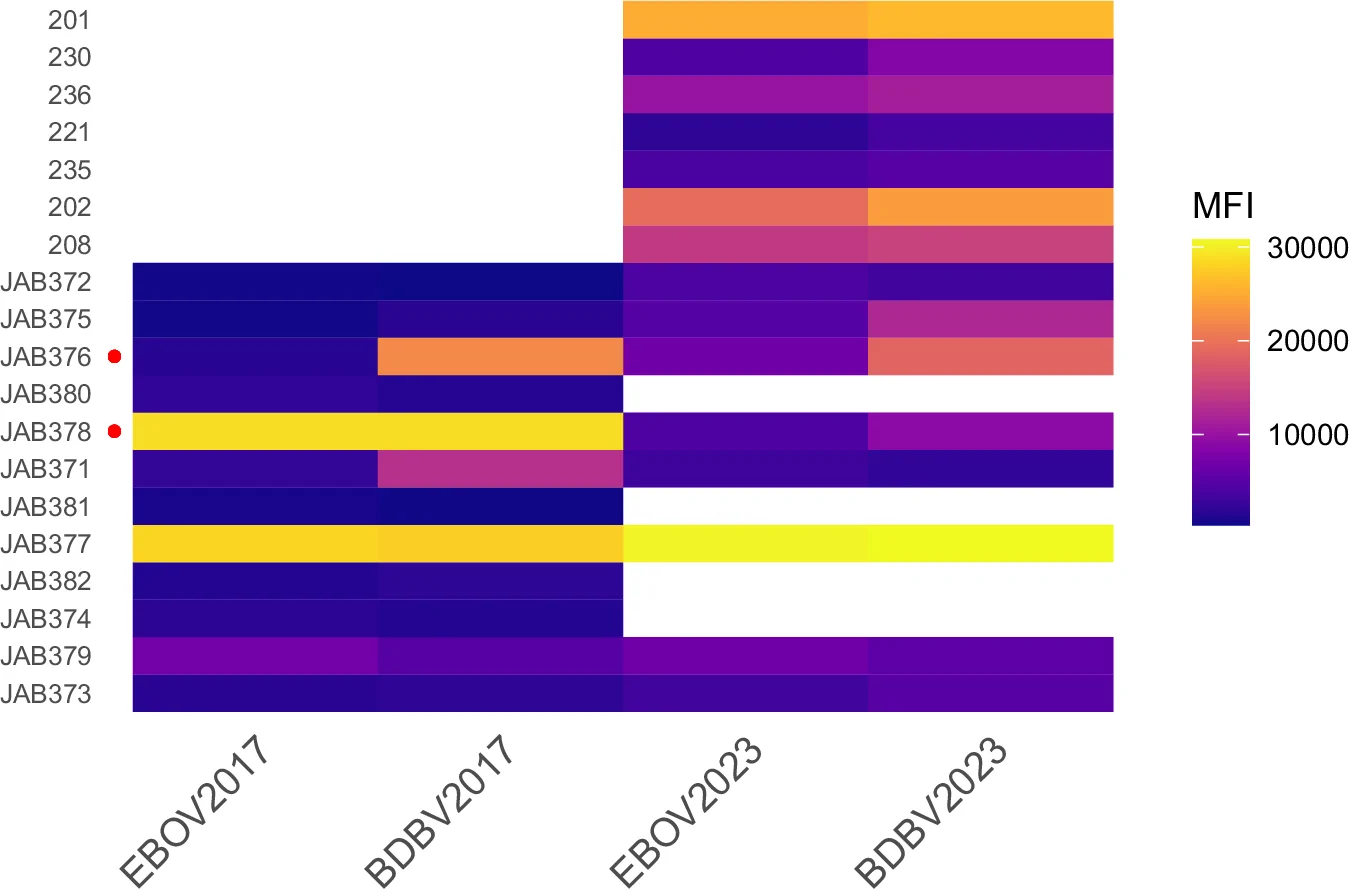
Individual IgG responses to Ebola (EBOV) and Bundibugyo (BDBV) virus amongst Guinean wildlife hunting communities from Village 4 in 2017 and 2023. IgG responses were measured by multiplex microsphere-based immunoassay (MMIA) as background-subtracted median fluorescence intensity (MFI). Individuals with a lower estimated NT50 for BDBV than EBOV in parallel lentiviral pseudotyped virus neutralization tests are denoted with a red dot. Abbreviations: IgG immunoglobulin G; NT50 50% neutralization titer.

Mean antibody responses were calculated for each filovirus antigen by village, and revealed marked village-level heterogeneity (Fig. 6). For example, Village 4 in 2017 showed significantly increased mean BDBV responses compared to other villages (*P* < 0.0001). Some of the highest BDBV-neutralizing individuals identified were located in Village 4 within the same household, and further, demonstrated superior binding and neutralizing activity against BDBV than EBOV in the MMIA and lentiviral pseudotyped neutralization assays (Fig. 7). Village 4 was confirmed by both official Macenta Ministry of Health designations and key informant interviews of healthcare workers and village leaders to be unaffected by the 2013–16 West African Ebola epidemic.

### Wildlife hunting and zoonotic filovirus exposure

Individual-level risk factors were investigated for the 2023 study population in univariable and multivariable models using questionnaire data. Sex was significantly associated with BDBV cluster seropositivity, with the odds of being in the BDBV cluster 2.49 times higher amongst males (95% CI: 1.00–6.16, *P* = 0.048) compared to females (Supplementary Table 8). Nonhuman primate consumption was associated with higher odds of BDBV cluster seropositivity in both univariable (OR = 3.49, 95% CI: 1.25–9.74, *P* = 0.017) and multivariable models (aOR = 5.03, 95% CI: 1.70–14.90, *P* = 0.004) (Supplementary Table 8).

### Influence of land cover patterns on exposure to zoonotic filoviruses in Guinean wildlife hunting communities

We extracted metrics of land cover (trees, crops, built area) and forest fragmentation (perimeter area ratio, shape-index and fractal dimension) from satellite-derived land-cover maps for 2017 and 2023 (Environmental Systems Research Institute, European Space Agency Sentinel-2)^49^. We extracted metrics at 500 m, 1 km, 2 km, 5 km and 10 km distances from the village centroids, which were examined for significance across varying spatial scales using a data-driven approach.

Mixed-effect multivariable logistic regression models were then used to examine the association between environmental drivers and risk for each zoonotic virus (based on their *K*-means-based antibody exposure profiles) and identified significant associations between landscape patterns and serological filovirus exposure. Models also examined individual risk factors reported in the questionnaire, including age, sex, occupation and active wildlife hunting; these did not significantly improve the model.

Significant environmental variables were identified at various spatial scales for all antigens, with forest coverage and configuration (shape index, perimeter area ratios, fractal dimension index) found to be associated with EBOV, BDBV, TAFV, LLOV and MLAV reactivity (Table 2). Differing landscape patterns were identified for each filovirus antigen, with EBOV exposure being negatively associated with tree coverage. Forest fragmentation (within a 500 m radius of the village) and living in built areas were positively associated with BDBV seroreactivity. Reactivity to other filovirus antigens (TAFV, LLOV) was positively associated with proximity to intact forest (5–10 km radius of the village).

**Table 2:** Significant associations between viral antigen exposure risk and environmental factors

Viral antigen	Land cover type	Metric	Scale, m	Adjusted OR (95% CI)	*P-*value
**EBOV**	Tree	Coverage (percentage)	2000	0.74 (0.59–0.93)	0.01
	Forest	Shape Index^1^	2000	0.82 (0.66–1.02)	0.07
	Forest	Perimeter Area Ratio^2^	10,000	0.86 (0.71–1.05)	0.13
**BDBV**	Built area	Coverage (percentage)	5000	3.29 (1.37–7.93)	0.008
	Forest	Shape Index^1^	500	1.37 (1.04–1.80)	0.02
**TAFV**	Forest	Fractal dimension	10,000	0.77 (0.62–0.96)	0.02
	Built area	Coverage (percentage)	5000	0.29 (0.09–0.91)	0.035
**LLOV**	Forest	Fractal dimension	5000	0.46 (0.22–0.95)	0.035

1. Shape index standardizes patch shape relative to a circle, where 1 represents a perfect circle and values > 1 indicate increasingly elongated or irregular shapes. 2. Perimeter-area ratio measures patch shape complexity, with higher values indicating more irregular or fragmented forest patches. 3. Fractal dimension quantifies boundary complexity, with values approaching 2 indicating increasingly complex and irregular patch boundaries. Environmental associations with filovirus seropositivity were assessed using generalized linear mixed-effects models fitted in R (v4.2.1) using the lme4 package (v1.1.37), with *K*-means cluster classifications used as binary seropositivity outcomes and sampling year included as a random effect. Two-sided Wald *z*-tests were used to assess regression coefficients. Environmental variables were extracted from ESRI European Space Agency Sentinel-2 land-cover classification maps (10-m spatial resolution) captured in 2017 and 2023. Study sites were geolocated to village centroids and environmental variables, including land-cover proportions and forest fragmentation metrics, were extracted at circular buffer radii of 500 m, 1 km, 2 km, 5 km and 10 km. Variables with *P*-values > 0.2 or high collinearity (Pearson correlation coefficient >0.75) were excluded prior to multivariable modeling. Iterative bidirectional stepwise model selection was performed using the Akaike information criterion (AIC) to identify best-fit multivariable models. No formal adjustment for multiple comparisons was applied because variables were evaluated within an exploratory AIC-based model selection framework. Reported effect sizes represent adjusted odds ratios with 95% confidence intervals. Abbreviations: *AIC* Akaike information criterion; *BDBV* Bundibugyo virus; *CI* confidence interval; *EBOV* Ebola virus; *ESRI* Environmental Systems Research Institute; *LLOV* Lloviu virus; *m* meters; *OR* odds ratio; *TAFV* Taï Forest virus.

## Discussion

We report a 6-year repeated cross-sectional study that collected serum samples in 2017 and 2023 from a high-risk population of wildlife hunters and close contacts residing within a rapidly deforesting area in Forested Guinea. Serological screening of the sera by a multiplex microsphere-based immunoassay against 9 filovirus glycoproteins revealed reactivity against not only EBOV (which caused a devastating outbreak in the region in 2013–16), but also other zoonotic filoviruses, including BDBV, TAFV, BOMV, RESTV, SUDV, LLOV and MLAV viruses. The study demonstrated the presence of EBOV GP-reactive antibodies at the population-level, 10 years on from the beginning of the 2013–2016 West African epidemic, supporting previous observations of durable anti-EBOV IgG binding and neutralization^41,42^ as well as ebolavirus species cross-reactivity^50^ and cross-neutralization^51^.

The use of an unsupervised machine-learning approach (K-means clustering) enabled the identification of distinct patterns of antibody responses across the study population. These patterns ranged from broadly reactive profiles, characterized by increased binding to multiple filovirus glycoproteins (e.g., clusters 6, 7, and 9), to more restricted profiles showing increased reactivity to a smaller subset of antigens (e.g., cluster 5, with reactivity to BDBV and EBOV), as well as clusters exhibiting species-specific reactivity patterns. Interpretation of serological profiles with binding to multiple antigens is inherently challenging, as such patterns may arise from heterotypic cross-reactivity, sequential or multiple infections, or exposure to antigenically intermediate (potentially novel) filoviruses^48,51^. Consistent with this, cluster assignments were not interpreted as definitive classifications of exposure. However, with the selected clustering resolution (*k* = 15), this approach enabled identification of small groups of individuals with species-specific antibody reactivity patterns, including clusters characterized by relatively higher reactivity to individual filovirus glycoproteins (e.g., clusters 4 [BDBV], 15 [TAFV], 8 [RESTV], 13 [MLAV], and 14 [LLOV]). Individuals within these clusters were subsequently prioritized for functional assessment and further differentiation by parallel pseudotyped virus neutralization assays (PVNA).

Five (5/10) individuals demonstrated higher neutralizing activity against BDBV than against EBOV-Makona virus. PVNAs were standardized to the extent possible (performed in parallel using identical protocols, controls, reagents, pseudoviral production methods and assay input titers); however, uncontrolled variables such as defective particle ratios, GP expression on plasma membranes, etc., may have introduced variability between assays. Even so, these findings provide serological evidence that these individuals were exposed to a virus more antigenically similar to BDBV than EBOV. Individual-level epidemiological data further support this hypothesis, as two of these individuals were marital partners residing in a village unaffected by EBOV. Their spousal relationship increases the probability that both were infected with the same BDBV-like virus, rather than each independently developing an atypical post-EBOV IgG response skewed towards cross-reactive epitopes. If the latter were true, and their polyclonal response primarily targeted highly-conserved structural features of the GP, it could be expected that these cross-reactive antibodies would recognize multiple members of the tested heterologous ebolavirus species, rather than being restricted to BDBV and EBOV. This hypothesis is further supported by spatial heterogeneity, with Village 4, which was EBOV-unaffected, found to have significantly higher mean BDBV antibody response in 2017 and 2023. As a final thread of evidence, nonhuman primate hunting was found to be positively associated with anti-BDBV antibody responses in multivariable analysis, as was the case in the 2012 BDBV outbreak in the Democratic Republic of the Congo, where many patients reported close contact with bushmeat^52^.

Other putative zoonotic filovirus spillover events, such as the TAFV virus, were also epidemiologically plausible, especially given the geographic proximity to the only known human case, which occurred following contact with a dead chimpanzee in neighboring Côte d’Ivoire. While there was some overlap of the NT50 95% confidence intervals, 5 (5/8) individuals who showed specific IgG binding against TAFV and minimal binding against EBOV also neutralized TAFV more strongly. Similar to BDBV, TAFV was found in 2023 to be spatially clustered in a village that was not affected by EBOV in 2013–16.

Other serological signatures of potential exposure were identified, including responses to viruses of unknown human infectivity and pathogenicity, including BOMV, which was recovered from free-tailed bats in neighboring Sierra Leone. While BOMV is known to circulate in bat reservoirs in the area, its spillover potential remains unknown. While wild-type BOMV GP does not efficiently mediate viral entry into human cells under experimental conditions, minimal mutations in the receptor-binding domain were required to improve viral entry and enhance binding to human NPC1, suggesting some potential for host-switching^53^. Pseudotyped virus neutralization assays showed similarly potent neutralization of both BOMV GP and EBOV GP pseudotyped viruses, which was a surprising finding given the lack of anti-EBOV binding antibodies identified by the MMIA screen. This may be due to differences in the presentation, availability or glycosylation of conformational epitopes on the soluble trimeric recombinant glycoprotein ectodomains conjugated to beads as compared to GPs decorating the envelope of a lentiviral particle. Taken together, these results may suggest infection with a virus antigenically equidistant, but distinct, from both EBOV and BOMV.

The study presents evidence of 2 individuals who demonstrated seroconversion to LLOV or a related virus occurring between 2017 and 2023, highlighting the value of repeated surveillance (Supplementary Fig. 3). Unlike other filoviruses, LLOV is non-endemic to the region, and only 1/8 individuals were found to exhibit more potent neutralization of LLOV (compared to EBOV). This may be interpreted cautiously as preliminary evidence for the circulation of an antigenically related cuevavirus in the region (rather than the circulation of the LLOV species, yet to be identified outside of Europe).

The overall seroreactivity seen against multiple filovirus species in this population is consistent with other studies, which identified evidence of EBOV circulation from Forested Guinea prior to the 2013–2016 outbreak^54^, implying ongoing spillover. A 2024 cross-sectional serosurvey in forested areas of rural Cameroon similarly identified high seroreactivities to Ebola and Sudan orthoebolaviruses despite no reported outbreaks, implying the silent circulation of filoviruses^45^.

Given the spatial heterogeneity of the data, we explored whether local land-use changes were associated with distinct patterns of filovirus-reactive antibody responses. Varying associations within landscape factors are potentially indicative of differing transmission pathways. For example, antibody profiles dominated by EBOV-reactivity were negatively associated with tree coverage and forest fragmentation, potentially reflecting the predominance of human-to-human transmission over zoonotic spillover pathways. Exposure to BDBV, a pathogen also noted for ongoing nonzoonotic transmission, was associated with living in built areas with very proximal deforestation (within 500 m), potentially reflecting the risk of encroachment into the forest. By contrast, reactivity to other filovirus pathogens (TAFV, LLOV, MLAV) was associated with areas near larger patches of intact forest, potentially reflecting exposure that occurs during hunting (where hunters may move deeper into the forest) as opposed to peridomestic transmission at forest-village edges. Our analyses were limited by the spatial resolution of the data (collected at the village level), and further investigation of this hypothesized transmission pathway through extensive population-based surveys and individual GPS tracking would better characterize how the interaction of hunting behaviors and the local environment may drive risk. Together, these highlight the potential role of local movements and habitat fragmentation in driving these spillover risks. Conservation in this context may protect local communities by serving as an ecological countermeasure against pathogen spillover.

A major limitation in the study design was the targeted sampling of wildlife hunting populations. While this may increase the probability of detecting zoonotic virus exposures, population-based surveys would be needed to assess wider exposure risks. Exposure to wildlife meat was ubiquitous among the Guinean study population, which severely limited our ability to examine associations between wildlife meat and virus exposure, independent of confounders such as socioeconomic status and remoteness. Despite this, differences in the relative risk posed by the consumption of different animal species (e.g., NHPs) were identified, likely reflecting the wildlife reservoirs or intermediate hosts of these pathogens.

Unfortunately, due to our age-based exclusion criteria, early-life exposure could not be evaluated, despite documented wildlife hunting practices (rodents) amongst children^55^. The value of such data is highlighted by Hounmenou et al., who identified anti-EBOV antibodies in children from rural western Guinea born after the 2013–16 outbreak, which are unlikely to be maternally derived, given their age^56^. In the future, higher resolution longitudinal measurements over a wider age range could allow for model-based estimates of seroconversion rates or a force of infection for these pathogens, and more accurate modeling of antibody decay.

As discussed above, our attempts to classify villages as either affected or unaffected by the 2013–14 West African Ebola outbreak will be biased by both underreporting and misdiagnosis of historical EBOV cases, due to social stigma, lack of access to healthcare facilities, and the endemicity of malaria and typhoid fever. Further, individual EBOV infection history is not known for the participants, as they were not subjected to PCR testing while symptomatic, and Ebola survivor certificates were not issued by local health authorities, which presented a significant challenge in the interpretation of serological results.

The use of serological methods for surveillance presents both limitations and strengths. While our interpretations of seroprevalence were complicated by cross-reactivity and potential infection of as-yet unidentified but antigenically similar viruses, and a lack of defined thresholds, serosurveys allow us to detect infrequent events in populations with limited healthcare access, and increase the chances of detecting non-pathogen-specific responses which may be indicative of novel spillover events. While authentic virus neutralization remains the gold standard for measuring the presence and magnitude of neutralizing antibodies, the biosafety handling requirements of the viruses in question meant that pseudotyped viruses were used as a surrogate, though they may imperfectly represent the authentic virus. Further, while comprehensive pseudotyped neutralization of all samples against the entire panel of filoviruses would have yielded further insight, this was not possible due to limitations in sera volume and assay throughput. Future serosurveillance studies may also benefit from incorporating additional filovirus antigens (e.g., NP (more highly conserved), VP40) in the MMIA.

Overall, our study investigated a large study population from a remote, high-risk community and successfully resampled 52.45% of the original group as part of a repeated cross-sectional study conducted 6 years later. By integrating detailed questionnaire data with behavioral, spatial, and serological analyses, we provided a broad characterization of this high-risk human-wildlife interface, highlighting potentially tractable risk factors such as the consumption of nonhuman primates. Analysis of high-resolution satellite-derived landcover data allowed us to identify different landscape factors associated with exposure to various pathogens, providing insight into potentially differing transmission pathways. We present multiple lines of serological and epidemiological evidence that suggest ongoing spillover of multiple zoonotic filoviruses in wildlife hunters in Forested Guinea. These findings highlight the critical importance of improving the health and economic security of communities living in hotspots while actively safeguarding ecosystems from further deforestation, degradation and fragmentation. Expanding surveillance in high-risk populations not only enables early detection of emerging pathogens but also deepens our understanding of the mechanisms driving their emergence. Strengthening local diagnostic capabilities through sustained capacity-building programs, collaborative partnerships, and the establishment of decentralized laboratory networks will be essential. This approach provides the foundation for designing socially acceptable, context-specific and effective risk mitigation strategies to address spillover at its source.

## Methods

### Ethical approvals and consent

For the wildlife hunter study population, 2017 and 2023, Guinée forestière, Republic of Guinea, ethical approval for the study was granted by the board of UK research ethics council, the national research for health ethic committee of Guinea (permit N°012/CENRS/2017 and N°135/CENRS/22) and the Oxford Tropical Research Ethics Committee (OxTREC:536-22), as has been described in the previous publication^46^. Written informed consent was obtained from all participants.

For the Ebola virus survivor and negative study population 2015 and 2017, Coyah and Guéckédou, Republic of Guinea, ethical approval was obtained from the National Ethics Committee for Health Research, Guinea (33/CNERS/15) and from the National Research Ethics Service, UK, as described in previous publications^41^. Written informed consent was obtained from all participants.

### Sample collection

#### Wildlife hunter study population, 2017 and 2023, Guinée forestière, Republic of Guinea

Recruitment of the 2017 Guinean wildlife hunter study population has been described in the previous publication^46^. Villages were selected based on location (deep within forested regions, proximity to large rivers) and purposively sampled to include those which were affected (*n* = 16 villages) and unaffected (*n* = 28 villages) by the 2013–2016 West African Ebola epidemic, as reported in national databases available at the Macenta Prefectural Health Directorates office. These designations were confirmed by key informant interviews with healthcare workers and village leaders. Within villages, families known to practice wildlife hunting were approached, and the senior hunter and their spouse (or next closest relative) were invited to participate. A total of 511 serum samples were collected in 2017 from 40 villages within 7 subprefectures of Macenta, Republic of Guinea. Blood processing was performed as described in Akoi Bore et al., 2024^46^. 5 mL of venous blood was collected, centrifuged at 2000 G for 10 min and serum was aliquoted and stored at −20° Celsius (C). Participants <18 years of age, pregnant, with chronic disease, or visitors to the region were excluded. Demographic data, including age, sex, location (to the village level), occupation, and exposure to bushmeat hunting (yes/no), were collected via questionnaires in French and Toma. In 2023, a total of 648 serum samples were collected from 43 villages, Macenta, Republic of Guinea, according to the same methods. Detailed questionnaires on wildlife hunting were administered in French and Toma.

#### Ebola virus survivor and negative study population 2015 and 2017, Coyah and Guéckédou, Republic of Guinea

The Ebola virus disease (EVD) survivor, contact and negative cohort comes from an observational longitudinal cohort study (2015–2023) of Ebola EVD survivors, contacts and negative controls based in Guéckédou (the outbreak epicenter) and Coyah (approximately 450 kilometers away), Republic of Guinea, as described in Thom et al^41^. Survivors were initially recruited from EVD survivor associations, and verified in EVD treatment center databases, or by the presentation of EVD survivor certificates. Individuals from the region who reported no history of Ebola virus disease symptoms, had no known exposure to infected cases, and no self-reported attendance at high-risk events (e.g., funerals) were included as negative controls.

#### Blood donor study population, 2020, United Kingdom

A total of 63 serum samples collected from healthy United Kingdom blood donors were used for this study. Samples were collected in 2020 from donors aged 17–80 years by the Scottish National Blood Transfusion Service (SNBTS) as described in Thompson et al., 2020^47^. Ethical approval was obtained for the SNBTS anonymous archive (IRAS project number 18005). SNBTS blood donors gave fully informed consent to virological testing: donation was made under the SNBTS Blood Establishment Authorization, and the study was approved by the SNBTS Research and Sample Governance Committee.

### Detecting IgG binding by multiplex microsphere-based immunoassay (MMIA)

An envelope glycoprotein (GP)-based multiplex microsphere immunoassay, consisting of nine filovirus GP antigens – EBOV, BDBV, BOMV, TAFV, SUDV, RESTV, RAVV, LLOV and MLAV – was produced and performed as described in Roe et al. 2024^48^.

### Pseudotyped filovirus lentivirus production

#### Plasmids

Pseudotyped lentiviruses displaying surface glycoproteins from EBOV, BDBV, BOMV, TAFV, SUDV, RESTV and LLOV were produced using a second-generation lentiviral (HIV-1) system using the backbone plasmid p8.91 (expressing Gag, Pol, Tat and Rev) as a packaging construct, and firefly luciferase (pCSFLW) as a reporter construct^57^. Envelope glycoproteins were codon optimized and cloned into pCAGGS or pCDNA3.1+ expression vectors. Viral envelope glycoprotein (GP) plasmids were kindly provided by Dr. Edward Wright, University of Sussex, Professor Nigel Temperton, University of Kent and Dr. Simon Scott, University of Kent (Supplementary).

#### Pseudotyped lentivirus production

In brief, filovirus glycoprotein expressing construct, gag/pol construct (p8.91) and luciferase reporter carrying construct (PCSFLW) were combined at a ratio of 1:1:1.5 uL with 0.3 µL of 1 µg/mL FuGENE HD transfection reagent per 100 ng DNA in 200 µL OptiMEM (Gibco™, 31985062). Transfections were performed in 10 mL of Dulbecco’s modified Eagle medium (DMEM) 1X, high glucose, GlutaMAX™ (Gibco™, 10569010) supplemented with 1% fetal bovine serum (FBS) (Gibco™, 16000044) and 1% penicillin–streptomycin (Sigma-Aldrich, P4333), incubated at 37 °C/5% CO_2_. Pseudotyped virus supernatant was harvested at 45–50 h and 70–75 h post-transfection and filtered through a 0.45 μm pore cellulose acetate filter. Titers of pseudotyped virus stock were quantified by luciferase-based transduction of target cells (HEK293T), and 50% Tissue Culture Infectious Dose (TCID50)/mL was calculated using the Spearman & Kärber formula^58^.

### Pseudotyped virus neutralization assay (PVNA)

Sera were tested in triplicate, in threefold serial dilutions (using Dulbecco’s modified Eagle medium supplemented with 1% fetal bovine serum (FBS) and 1% penicillin–streptomycin) started at 1:20 (1/20-1/14580) for a volume of 90 µL per well, and incubated with 150 TCID50/well of pseudotyped virus for 1 h at 37 °C, 100 μL of the serum-pseudotyped virus mixture were added in triplicate to each confluent HEK293T/17 cells (American Type Culture Collection, CRL-3216) in a white Nunc™ MicroWell™ 96-well, flat-bottom microplate and incubated at 37 °C. Luminescence was measured 60 h post-infection using the Bright-Glo™ luciferase assay system (Promega) on a GloMax® Explorer Multimode Microplate Reader. All plates contained pseudotyped virus-only and cell-only controls.

Neutralization assay data were analysed in GraphPad Prism 10.1.2. Raw data from the pseudotyped neutralization assay were normalized as a percentage (%) neutralization relative to mean values for pseudotyped virus-only controls (equivalent to 0% infectivity) and cell-only controls (equivalent to 100% infectivity). The half maximal neutralizing titer (NT50) was interpolated by a 4-parameter nonlinear regression with a Hill-Slope constraint of less than 0, as previously described^59^. NT50 values and corresponding 95% confidence intervals were derived from curve fits to serial-dilution replicate data for each sample.

### Statistical analysis

#### K-means clustering

*K*-means clustering was applied as an unsupervised analytical approach to explore patterns of antibody reactivity (based on the anti-filovirus GP MFI values from the MMIA) within the Guinean wildlife hunter study population, to assess multiple exposure patterns without imposing binary seropositivity thresholds for single antigen responses, which can be problematic in the presence of heterotypic cross-reactivity and variable background reactivity.

An unsupervised machine learning algorithm, *k*-means clustering, was utilized to partition the data set into *k* groups. Analysis was performed in R version 4.2.1 (2022-06-23) using the cluster (version 2.1.4) and factoextra (version 1.0.7) packages^60,61^. The number of clusters (k) was evaluated across a range of values (*k* = 4–25) using multiple validation metrics, including within-cluster sum of squares (elbow method), gap statistic, average silhouette width, Calinski–Harabasz index, and Davies–Bouldin index, and visual assessment for biological interpretability^62,63^. Based on this integrated assessment, *k* = 15 was selected. Cluster assignments were used in an exploratory, hypothesis-generating manner to summarize population-level patterns and to guide targeted downstream analyses, including selection of samples for pseudotyped virus neutralization assays, and were not treated as definitive classifications of exposure. All clusters are reported and visualized.

#### Spatial mapping and geostatistical investigation of environmental risk factors

R version 4.2.1 (2022-06-23) landscapemetrics (version 2.1.4)^64^ were used to extract landscape characteristics and examine for associations with serological exposure to viral antigens, as described in previous publications and the Supplementary Methods^65^. A generalized linear mixed-effects model (GLM) was fitted in R using the lme4 (version 1.1.37) package^66^ for each individual filovirus antigen using *K*-means cluster classifications as binary seropositivity outcomes. Sampling year was included as a random effect within the model, and iterative stepwise model selection was utilized to develop the multivariable model of best fit, assessing each demographic and environmental variable for inclusion based on Akaike information criteria (AIC).

### Reporting summary

Further information on research design is available in the Nature Portfolio Reporting Summary linked to this article.

## Supplementary information

Supplementary Information

Reporting Summary

Transparent Peer Review file

## Data Availability

The data generated in this study include individual-level demographic, behavioral, serological and geospatial data from human participants and are available under restricted access due to ethical and privacy considerations related to participant confidentiality and the potential identifiability of study communities. De-identified data supporting the findings of this study may be made available to qualified researchers for non-commercial research purposes following approval by the relevant institutional and national ethics committees in Guinea and the United Kingdom. Requests for access should be directed to the corresponding authors, Grace Hood (grace.hood@well.ox.ac.uk) or Miles Carroll (miles.carroll@ndm.ox.ac.uk), and will be reviewed within approximately 6–8 weeks. The sequence data used in this study are publicly available in GenBank. The GenBank accession numbers for all viral glycoprotein sequences used in this study are as follows. For the multiplex microsphere-based immunoassay antigens: Ebola virus glycoprotein (EBOV GP), NC_002549.1; Bundibugyo virus glycoprotein (BDBV GP), FJ217161.1; Taï Forest virus glycoprotein (TAFV GP), NC_014372; Bombali virus glycoprotein (BOMV GP), YP_009513277.1; Sudan virus glycoprotein (SUDV GP), NC_006432.1; Reston virus glycoprotein (RESTV GP), AF522874.1; Lloviu virus glycoprotein (LLOV GP), NC_016144.1; and Mengla virus glycoprotein (MLAV GP), KX371887.2. For the envelope glycoprotein expression plasmids: Bundibugyo virus glycoprotein (BDBV GP), NC_014373; Bombali virus glycoprotein (BOMV GP), MF319185.1; Ebola virus Makona glycoprotein (EBOV Makona GP), KJ660347.1; Reston virus glycoprotein (RESTV GP), AY769362; Sudan virus glycoprotein (SUDV GP), FJ968794; Taï Forest virus glycoprotein (TAFV GP), FJ217162; and Lloviu virus glycoprotein (LLOV GP), OQ630505.1.
