# Supplementary material for: Filovirus reactive antibodies in wildlife hunting communities suggest ongoing zoonotic spillover in forested Guinea
Source: Nat Commun. 2026 Oct 8;17:9974. doi: 10.1038/s41467-026-77653-3 (PMC13650133; doi:10.1038/s41467-026-77653-3)
Supplement: Supplementary file 1 [file 41467_2026_77653_MOESM1_ESM.pdf]

SUPPLEMENTARY INFORMATION

SUPPLEMENTARY METHODS

Recombinant envelope glycoprotein ectodomains

In brief, 9 soluble, native-like, trimeric filovirus envelope glycoprotein ectodomains (Supplementary Table 1) were produced in a mammalian cell-culture system (FreeStyle 293 Expression System) [67, 68]. Full-length glycoprotein sequences were identified in the NCBI database, transmembrane domains were removed, and remaining ectodomains were human codon optimized and designed with a C-terminal trimeric coiled-coil GCN3 motif (MKQIEDKIEEILSKIYHIENEIARIKKLIGE) followed by a double factor Xa protease cleavage site and Twin-Strep-tag (TST) or S-Tag flanked by 5' BamHI and 3' XbaI restriction enzyme sites. Repeating factor Xa protease cleavage sites (IEGRIEGR) were engineered immediately upstream of the tag to facilitate protein tag removal post-purification. A mock protein antigen stable cell line was also established (expression vector without the glycoprotein open reading frame transfected into the mammalian cell-culture system), purified, and cell culture supernatant was used as a measure of non-specific binding.

Trimeric glycoprotein ectodomain purification

Trimeric glycoprotein ectodomain purification was performed by affinity chromatography and size-exclusion chromatography. Proteins were concentrated and a buffer exchange step used to remove trace materials (serum, cells or cellular debris). Protein tags (TST or S-tag) were removed and western and Coomassie protein blots were performed to confirm removal. Concentration was determined by NanoDrop. Reactivity was determined by western blot. Trimeric filovirus GP ectodomains were then coupled to carboxylated magnetic microspheres (15 µg of antigen per 1.25 x 10^6 microspheres) according to manufacturer (MagPlex) guidelines.

Multiplex microsphere-based immunoassay

Human sera were screened for IgG binding against filovirus envelope attachment proteins using a multiplex microsphere immunoassay (MMIA). Sera diluted 1:400 in PBS without calcium and magnesium and heat inactivated at 60°C for 30 minutes. Following heat inactivation, diluted sera were incubated at room temperature (22–25°C) with a master mix of 1:100 diluted antigen-coupled microspheres in a 96-well microtiter plate for 45 minutes with agitation at 900 RPM. Wells were washed with PBS and 0.05% Tween20 (PBST) and subsequently incubated with 100 µL of biotinylated cross-adsorbed anti-human IgG (Invitrogen, Catalogue number: A18815) diluted 1:5000 in PBST per well for 45 minutes with agitation at 900 RPM. Following secondary incubation, wells were washed with PBST and incubated with 100 µL of streptavidin-phycoerythrin (Bio-Rad) diluted 1:1000 in PBST for 45 minutes with agitation (900 RPM). After tertiary

incubation, wells were washed with PBST and resuspended in 100 µL PBST for 10 minutes at 900 RPM. Where plates could not be immediately read, they were stored at 4°C overnight, then agitated at room temperature (22–25°C) for 30 minutes at 900 RPM prior to reading. Antigen specific binding IgG was quantified on a BioPlex 200 HTF multiplexing system as Median Fluorescent Intensity (MFI). Reported MFI values are adjusted to PBS control well averages.

Calibration of internal reference to WHO standard for EBOV binding antibody concentration

An internal positive control from EBOV was created from pooled serum from 3 PCR-confirmed Ebola Virus Disease (EVD) survivors from the EVD cohort described in Thom et al [41].

Establishment of threshold cut-offs for filovirus IgG by MMIA

Initial seropositivity cut-offs for individual filoviruses (n=9) were based on the presumed unexposed non-endemic cohort (UK blood donor) by calculating five standard deviations above the background subtracted (MFI-bg) mean.

Spatial mapping and geostatistical investigation of environmental risk factors

The land cover classification map was obtained from ESRI European Space Agency Sentinel-2 remote-sensing images captured at a 10-meter resolution in 2017 and 2023 [48]. Mutually-exclusive, standardised discrete land cover classifications were used (trees, water, flooded vegetation, crops, built area, bare ground, snow/ice, clouds and rangeland).

Study sites were geolocated to centroids and environmental data (proportion of land cover type, and measures of forest fragmentation (perimeter area ratio, shape index, and fractal dimension index) [69, 70]) were mean-centred, scaled, and extracted at circular buffer radii of 500 m, 1 km, 2 km, 5 km and 10 km from the village centroid to capture how the strength of association varies by spatial scale [65, 71]. A data-driven approach was utilized to identify environmental risk factors, as previously described in the literature [65, 72]. Mixed-effects logistic regression models were used to examine each environmental variable at each scale (n=126 models) for each individual antigen and variables which were insignificant (p-values >0.2) or highly correlated (Pearson correlation coefficient >0.75) were removed. All statistical analyses were performed with R version 4.2.1 (2022-06-23). A generalised linear mixed-effects model (GLM) was fitted in R using the lme4 (version 1.1.37) package for each individual filovirus antigen using K-means cluster classifications as binary seropositivity outcomes. Sampling year was included as a random effect within the model and iterative bidirectional step-wise model selection was utilized to develop the multivariable model

of best fit, assessing each demographic and environmental variable for inclusion based on Akaike information criteria (AIC).

SUPPLEMENTARY FIGURES

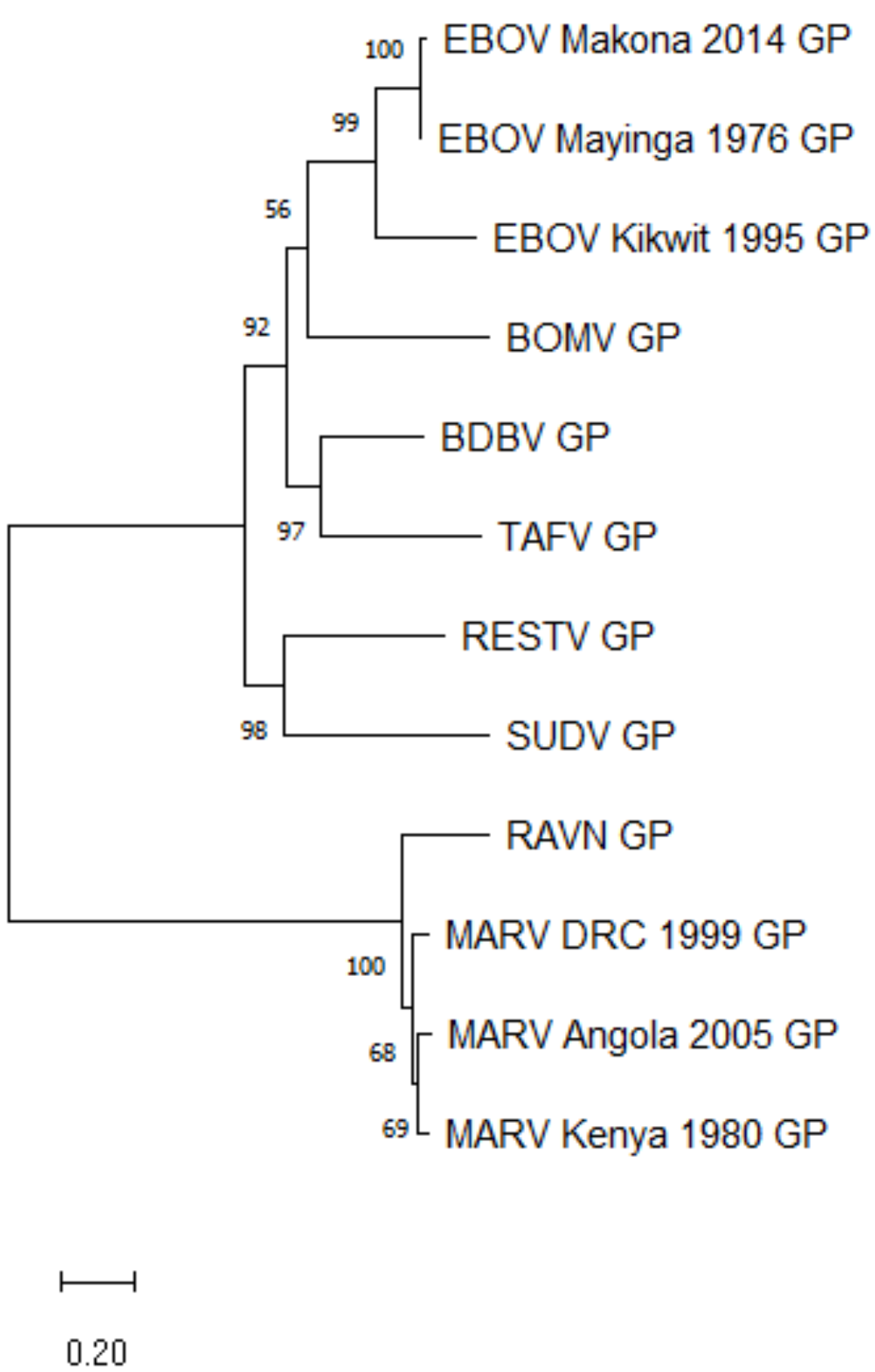

Supplementary Figure 1 Phylogenetic tree of filovirus envelope glycoproteins (GP) by Maximum Likelihood method and General Time Reversible model. Abbreviations: BDBV, Bundibugyo virus; BOMV, Bombali virus; DRC, Democratic Republic of the Congo; EBOV, Ebola virus; GP, glycoprotein; MARV, Marburg virus; RAVN, Ravn virus; RESTV, Reston virus; SUDV, Sudan virus; TAFV, Taï Forest virus.

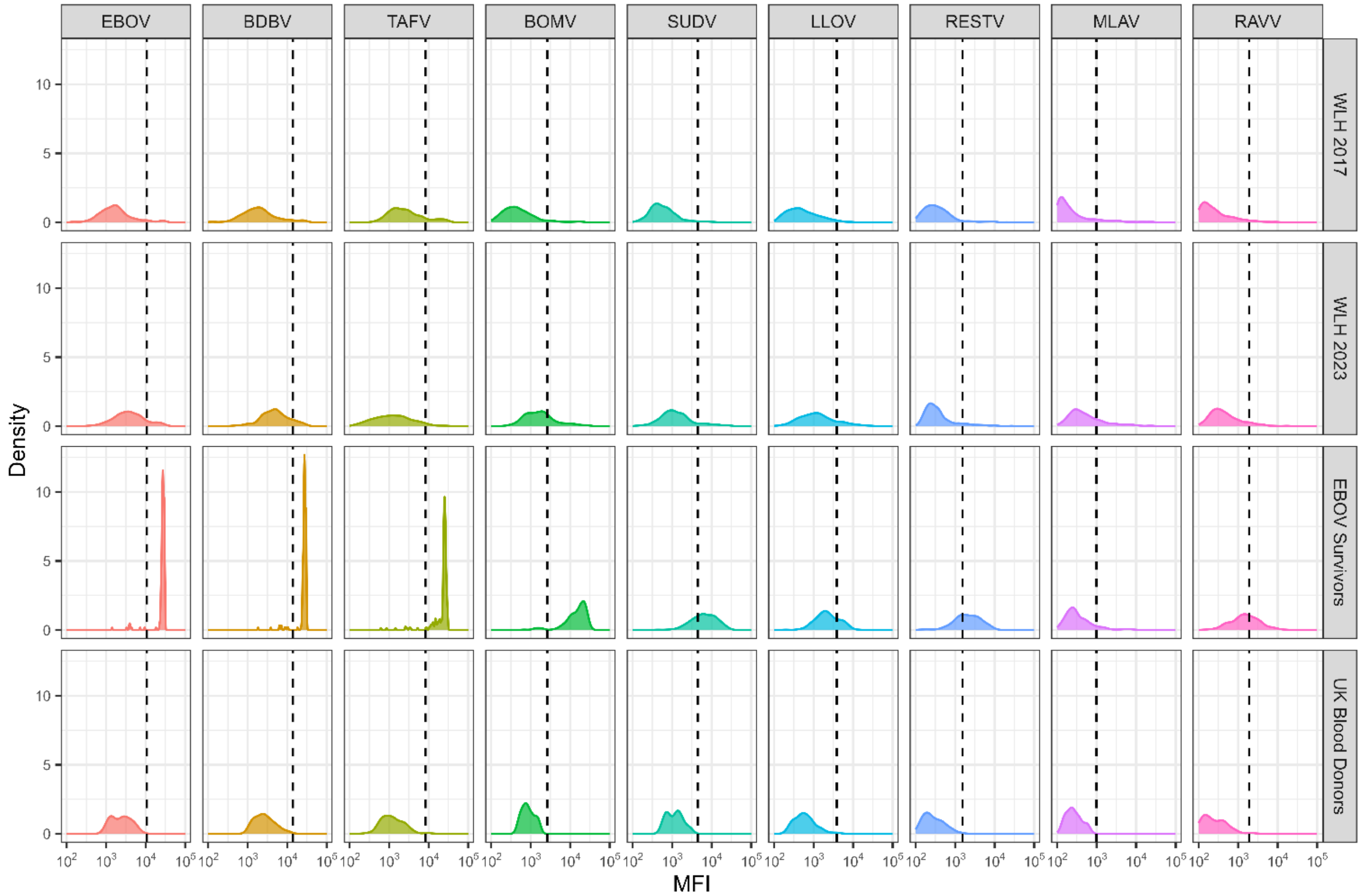

Supplementary Figure 2 Probability density plots of anti-filovirus glycoprotein IgG distributions among Guinean wildlife hunters 2017 (n=511) and 2023 (n=648), United Kingdom blood donor cohort (n=63), and Ebola virus survivors (n=3). IgG responses were measured by multiplex microsphere-based immunoassay (MMIA) in background subtracted median fluorescence units (MFI-bg) on the Luminex platform. Dashed vertical lines mark seropositivity cutoffs based on the presumed unexposed non-endemic cohort (UK blood donor) by calculating five standard deviations above the background subtracted (MFI-bg) mean.   Abbreviations: BDBV, Bundibugyo virus; BOMV, Bombali virus; EBOV, Ebola virus; IgG, immunoglobulin G; LLOV, Lloviu virus; MLAV, Měnglà virus; MFI, median fluorescence intensity; MFI-bg, background-subtracted median fluorescence intensity; MMIA, multiplex microsphere-based immunoassay; RAVV, Ravn virus; RESTV, Reston virus; SUDV, Sudan virus; TAFV, Taï Forest virus; UK, United Kingdom; WLH, wildlife hunters.

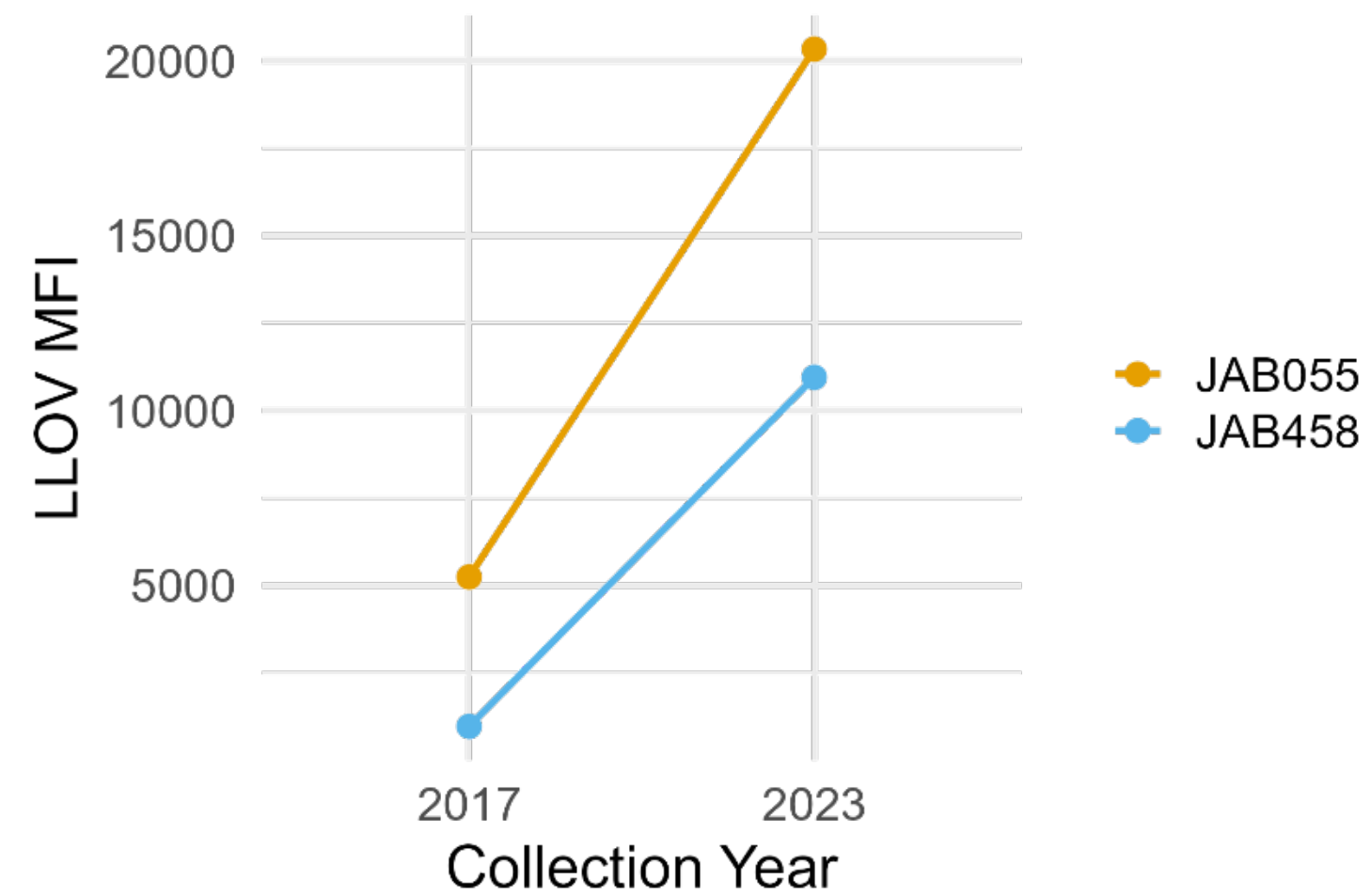

Supplementary Figure 3 Individual anti-filovirus glycoprotein IgG responses of Guinean wildlife hunters in 2017 and 2023 against LLOV. IgG responses measured by multiplex microsphere-based immunoassay (MMIA) in background subtracted median fluorescence units (MFI-bg) on the Luminex platform. Abbreviations: IgG, immunoglobulin G; LLOV, Lloviu virus; MFI, median fluorescence intensity; MFI-bg, background-subtracted median fluorescence intensity; MMIA, multiplex microsphere-based immunoassay. JAB followed by a number refers to the sample ID.

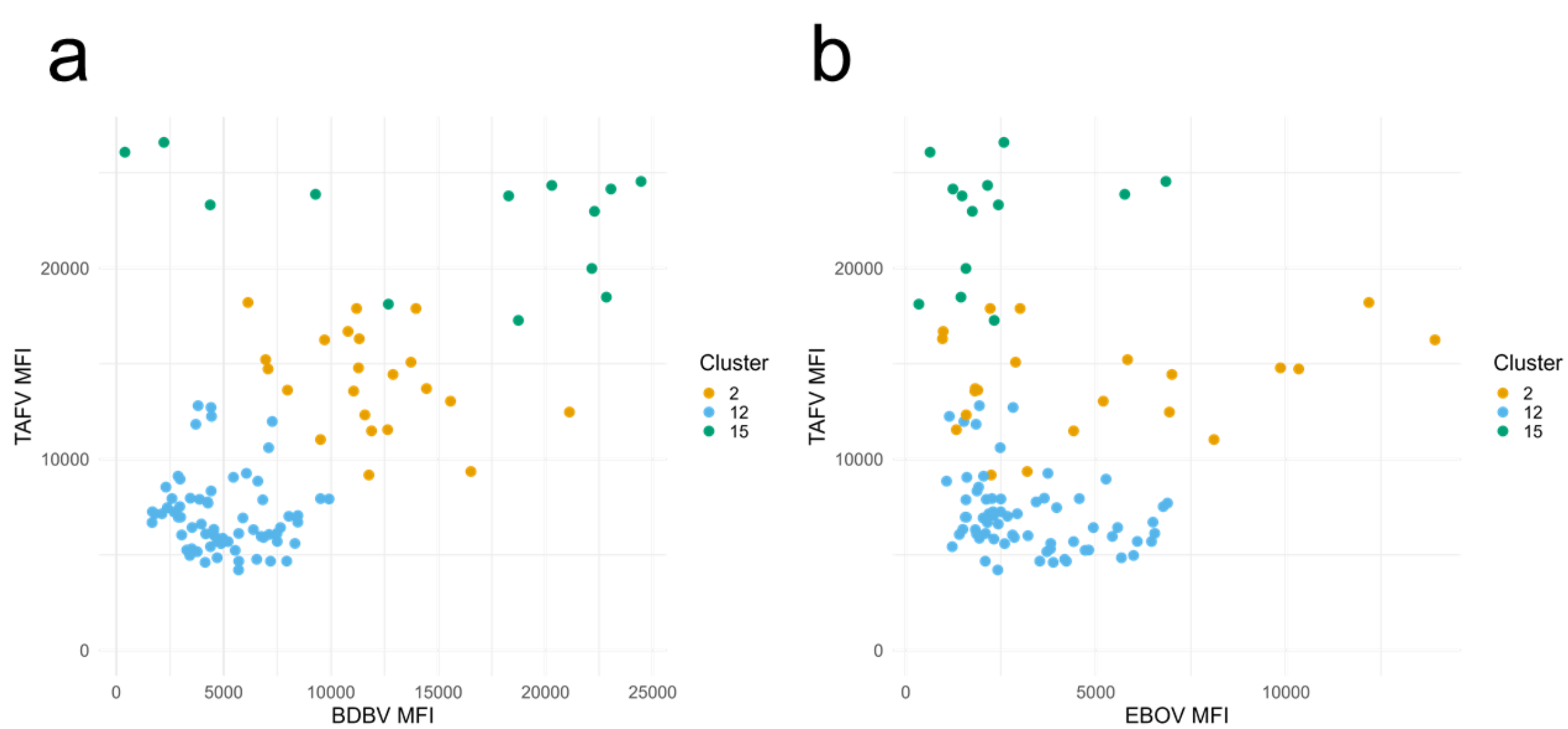

Supplementary Figure 4 Individual MFI responses against (a) TAFV and BDBV (b) TAFV and EBOV for all hunters from the Guinean wildlife hunting cohort (2017 and 2023) who were categorised into Clusters 2, 12 and 15 from the K-means clustering algorithm analysis for k=15. Abbreviations: BDBV, Bundibugyo virus; EBOV, Ebola virus; MFI, median fluorescence intensity; TAFV, Taï Forest virus.

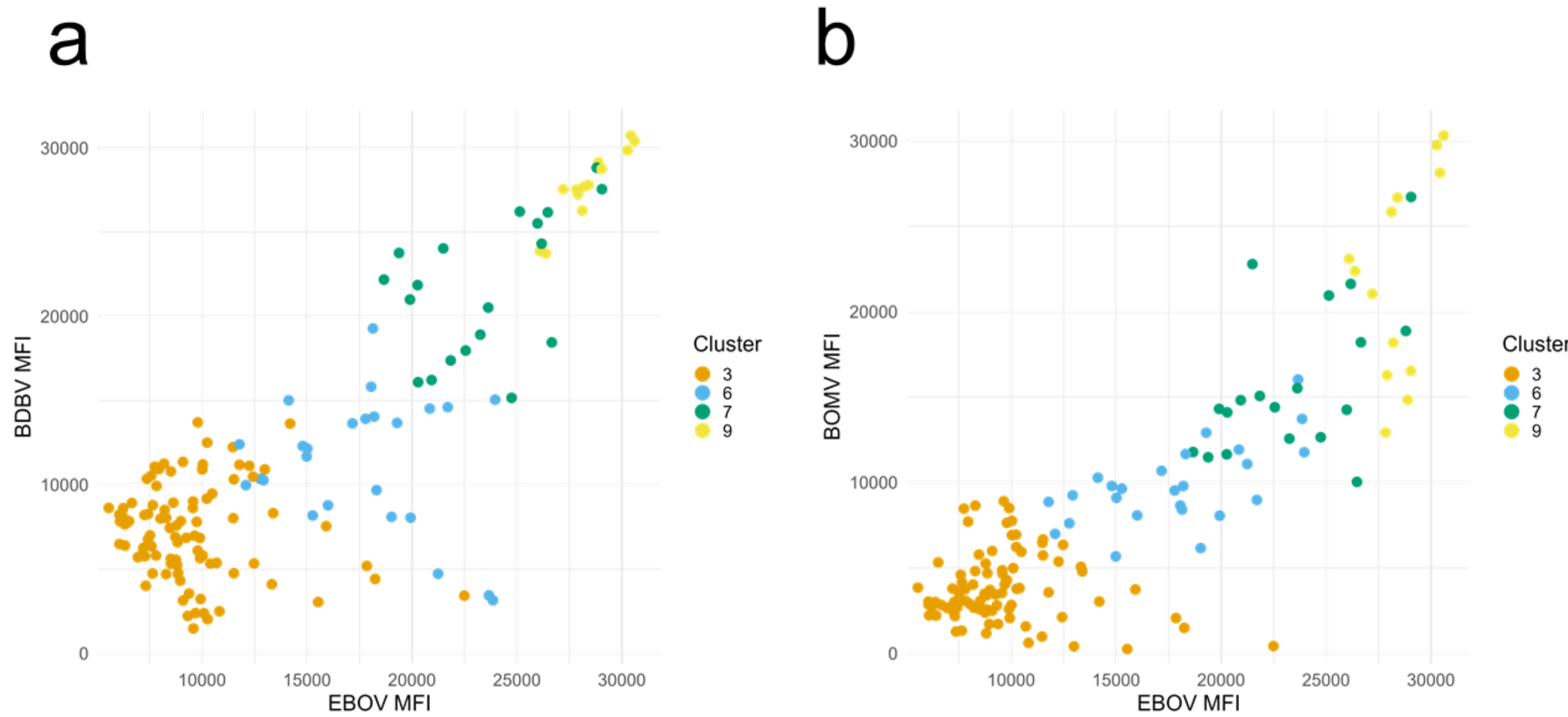

Supplementary Figure 5 Individual MFI responses against (a) BDBV and EBOV (b) BOMV and EBOV for all hunters from the Guinean wildlife hunting cohort (2017 and 2023) who were categorised into Clusters 3, 6, 7 and 9 from the K-means clustering algorithm analysis for k=15. Abbreviations: BDBV, Bundibugyo virus; BOMV, Bombali virus; EBOV, Ebola virus; MFI, median fluorescence intensity.

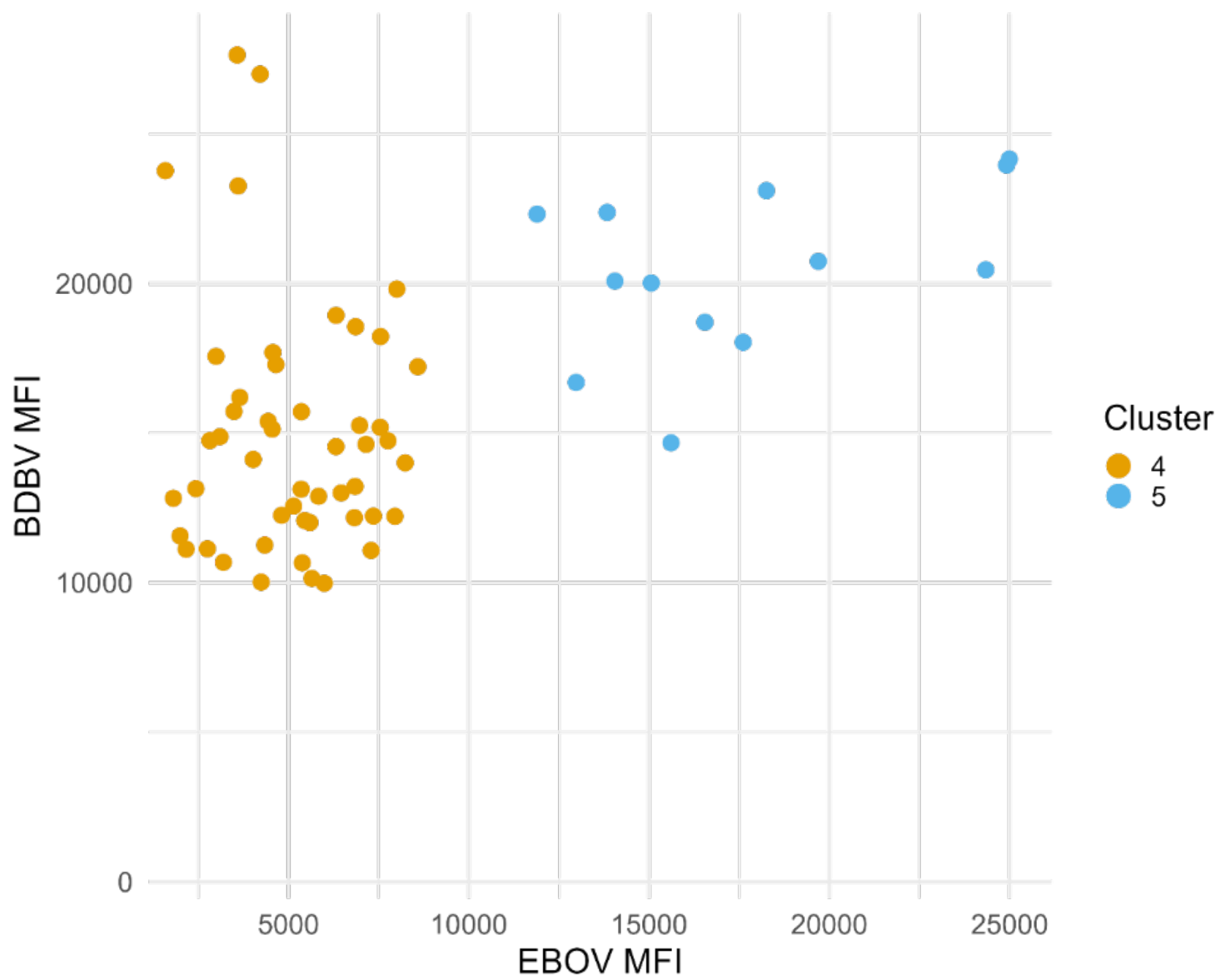

Supplementary Figure 6 Individual MFI responses against BDBV and EBOV from the Guinean wildlife hunting cohort (2017 and 2023) who were categorised into Clusters 4 and 5 from the K-means clustering algorithm analysis for k=15. Abbreviations: BDBV, Bundibugyo virus; EBOV, Ebola virus; MFI, median fluorescence intensity.

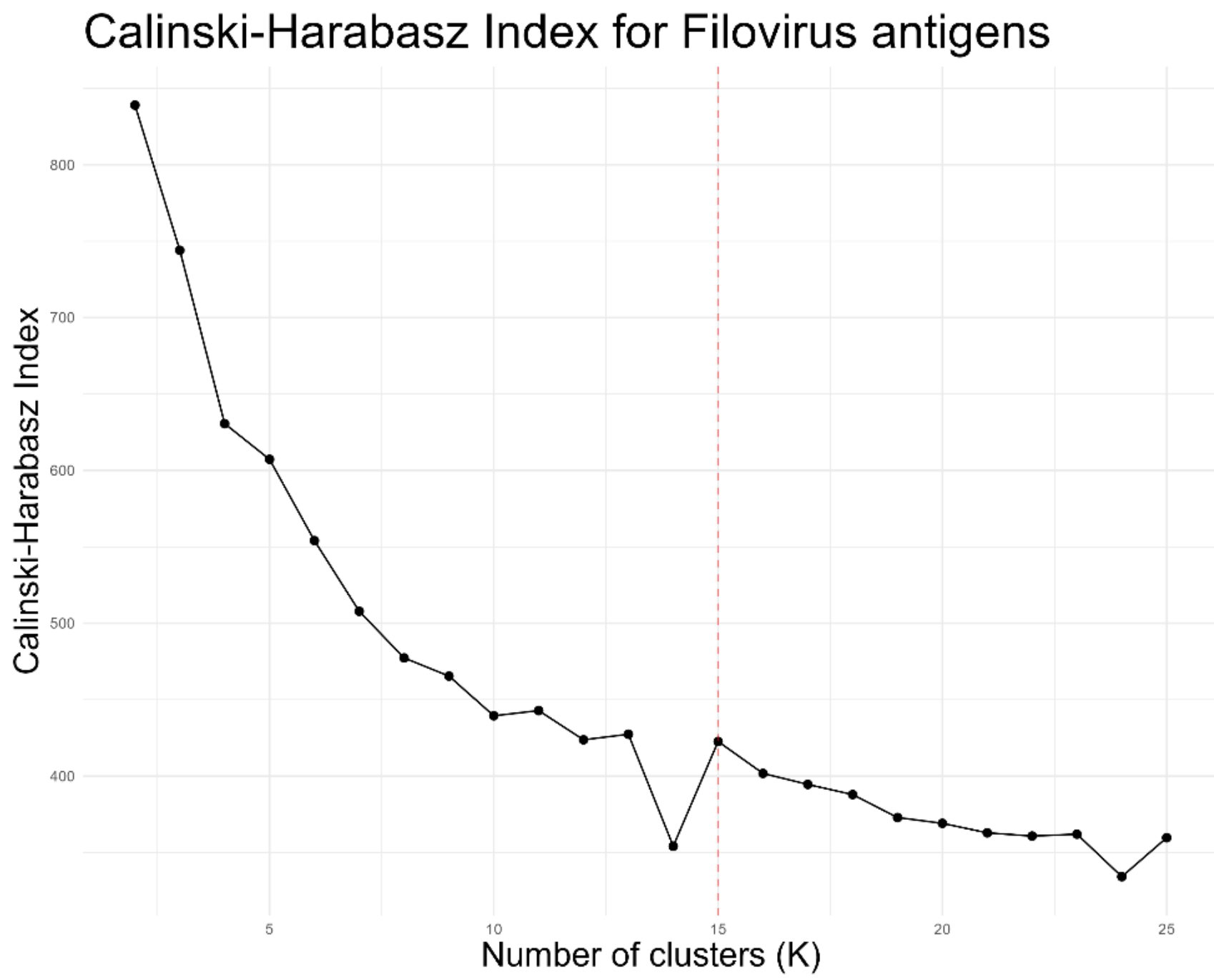

Supplementary Figure 7 Calinski-Harabasz index for K-means clustering for k=1-25.

SUPPLEMENTARY TABLES

Supplementary Table 1 Multiplex Microsphere-based Immunoassay antigens Abbreviations: BDBV, Bundibugyo virus; BOMV, Bombali virus; EBOV, Ebola virus; GP, glycoprotein; LLOV, Lloviu virus; MLAV, Měnglà virus; RESTV, Reston virus; SUDV, Sudan virus; TAFV, Taï Forest virus; N/A, not applicable.

Antigen	Virus isolate	Genbank Accession no.
EBOV GP	Ebola virus/H.sapiens/COD/1976/Yambuku-Mayinga	NC_002549.1
BDBV GP	Bundibugyo virus/H. sapiens/UGA/2007	FJ217161.1
TAFV GP	Tai¯ Forest virus/H. sapiens/COV/1994/Pauleoula-CI	NC_014372
BOMV GP	N/A	YP_009513277.1
SUDV GP	Sudan virus/H. sapiens/UGA/2000/Gulu-808892	NC_006432.1
RESTV GP	Reston virus/M. fascicularis/USA/1989/Pennsylvania	AF522874.1
LLOV GP	Lloviu virus/M.schreibersii-wt/ESP/2003/Asturias-Bat86	NC_016144.1
MLAV GP	Menglavirus/R. leschenaultii/CHN/2015/Sharen-Bat9447-1	KX371887.2

Supplementary Table 2 Envelope Glycoprotein (GP) plasmids Abbreviations: BDBV, Bundibugyo virus; BOMV, Bombali virus; CHO, Chinese hamster ovary; EBOV, Ebola virus; GP, glycoprotein; HEK293T, human embryonic kidney 293T; LLOV, Lloviu virus; RESTV, Reston virus; SUDV, Sudan virus; TAFV, Taï Forest virus.

Viral glycoprotein	GenBank Accession number	Expression cloning vector	Target cells	Source
**Filoviruses**				
**BDBV GP**	NC_014373	pCAGGS	HEK293T/CHO	Dr. Edward Wright, University of Sussex
**BOMV GP**	MF319185.1	pCAGGS	HEK293T/CHO	Dr. Edward Wright, University of Sussex
**EBOV Makona GP 14**	KJ660347.1	pCAGGS	HEK293T/CHO	Dr. Edward Wright, University of Sussex
**RESTV GP**	AY769362	pCAGGS	HEK293T/CHO	Dr. Edward Wright, University of Sussex
**SUDV GP**	FJ968794	pCAGGS	HEK293T/CHO	Dr. Edward Wright, University of Sussex
**TAFV GP**	FJ217162	pCAGGS	HEK293T/CHO	Dr. Edward Wright, University of Sussex
**LLOV GP**	OQ630505.1	pCAGGS	HEK293T	Dr. Simon Scott, University of Kent

Supplementary Table 3 Wildlife exposure questionnaire responses, Forested Guinea 2023. Abbreviations: IQR, interquartile range.; Min, minimum; Max, maximum.

Question	n	N	%
**Do you ever hunt or trap bats?**			
Yes	156	641	24
No	485	641	76
**Do other members of your household hunt or trap bats?**			
Yes	51	491	10
No	440	491	90
**Do you handle bats during preparation or cooking for food?**			
Yes	274	535	51
No	261	648	40
**Do you buy bats from other people or markets?**			
Yes	130	646	20
No	516	648	80
**Which season do you hunt more frequently?**			
Start of dry season	122	133	92
Middle of dry season	4	133	3
End of dry season	5	133	4
Start of rainy season	2	133	2
Middle of rainy season	0	133	0
End of rainy season	0	133	0
**What do you do with the hunted bats?**			
For food only	145	155	94
For food and to sell	8	155	5
Other	1	155	1
To sell only	1	155	1
**Do you ever go into bat caves?**			
Yes	139	546	25
No	407	546	75
**Do you do any other kind of activities with the bats when inside the cave?**			
No	85	86	99
Yes	1	86	1
**On a normal visit, how long (minutes) do you spend inside the cave?**			
Median [IQR]	2 [0 - 20]		
Mean [Min - Max]	13 [0 - 60]		
**Do you harvest bat guano?**			
Yes	2	648	0
No	646	648	100

Supplementary Table 4 Resampling of Guinean wildlife hunters from 2017, in 2023. Abbreviations: BDBV, Bundibugyo virus; EBOV, Ebola virus; LLOV, Lloviu virus; MLAV, Měnglà virus; RESTV, Reston virus; TAFV, Taï Forest virus.

***K*-means based classification**	**Count (individuals)**	**Percentage of total population (%)**	**Number resampled (in 2023)**
EBOV clusters	150	13.29	47
BDBV clusters	62	5.49	28
TAFV clusters	98	8.68	14
RESTV clusters	4	0.35	1
MLAV clusters	8	0.71	1
LLOV clusters	8	0.71	2

Supplementary Table 5 Demographic information for Guinean wildlife hunters tested by pseudotyped neutralisation assay against Bundibugyo virus (BDBV) and Ebola virus (EBOV) (that showed equal or stronger neutralisation against BDBV) Abbreviations: BDBV, Bundibugyo virus; EBOV, Ebola virus; ID, identification. JAB followed by a number refers to the sample ID.

**Sample ID**	**Sampling year**	**Village**	**Village EBOV status**	**Epidemiological information**
**JAB371**	2017	4	Unaffected	
**JAB135**	2017	23	Unaffected	Spouse of JAB136
**JAB378**	2017	4	Unaffected	
**JAB136**	2017	23	Unaffected	Spouse of JAB135
**JAB424**	2017	11	Affected	
**JAB376**	2017	4	Unaffected	
**JAB325**	2017	18	Unaffected	

Sample ID	Sampling year	Village	Village EBOV status	Epidemiological information
**JAB297**	2017	12	Unaffected	
**JAB375**	2017	4	Unaffected	

Supplementary Table 6 Demographic information for Guinean wildlife hunters tested by pseudotyped neutralisation assay against Taï Forest virus (TAFV) and Ebola virus (EBOV) (that showed equal or stronger neutralisation against TAFV) Abbreviations: EBOV, Ebola virus; ID, identification; TAFV, Taï Forest virus.

Sample ID	Sampling year	Village	Village EBOV status
**139**	2023	12	Affected
**751**	2023	41	Unaffected
**743**	2023	41	Unaffected
**685**	2023	30	Unaffected
**755**	2023	35	Unaffected

Supplementary Table 7 Sampling information on number of individuals approached, and participant numbers per village Abbreviations: ID, identification.

Village ID	Number of volunteers sampled	Number of individuals approached
1	12	2495
2	12	1568
3	6	2159
4	12	1427
5	10	1913
6	10	639
7	11	1877
8	7	3840
9	7	548
10	15	3173
11	16	2194
12	2	124
12	14	3117
13	18	3056
14	18	510
15	17	2909
15	6	2145
16	21	1033
17	12	270
18	12	463
19	5	104
20	10	2587
21	19	955
22	13	326
23	10	1084
24	13	136
25	15	3901
26	9	129
27	2	58
27	6	136
28	24	142
29	18	187
30	23	102
31	25	223
32	12	1157
33	2	52
34	6	112
35	12	241
36	6	628
37	6	524
38	8	140
39	6	189
40	29	389

Supplementary Table 8 Univariable and multivariable logistic regression analysis of BDBV exposure (n=62) in Guinean wildlife hunter (WLH) participants, 2023

Variable	Univariable regression Guinean WLH participants 2023		Multivariable regression Guinean WLH participants 2023	
	OR (95% CI)	*p*-value	aOR (95% CI)	*p*-value
**Age**	1.02 (0.99 – 1.05)	0.087	-	-
**Sex**	2.49 (1.00 – 6.16)	0.048	-	-
**Wildlife hunting**	1.05 (0.91 – 1.10)	0.982	-	-
**Perimeter area ratio (Forest) 2000 m**	0.55 (0.35 – 0.86)	0.010	0.67 (0.41 – 1.09)	0.110
**Built Area 10,000 m**	3.14 (1.39 – 7.08)	0.010	2.34 (0.91 – 6.00)	0.080
**Nonhuman primate hunting**	3.50 (1.26 – 9.76)	0.017	5.03 (1.70 – 14.90)	0.004

Abbreviations: AIC, Akaike information criterion; aOR, adjusted odds ratio; BDBV, Bundibugyo virus; CI, confidence interval; OR, odds ratio; WLH, wildlife hunter.

Univariable and multivariable generalized linear mixed-effects logistic regression analyses of factors associated with BDBV seropositivity in Guinean wildlife hunters sampled in 2023 (n=62 seropositive individuals). K-means cluster classifications were used as binary seropositivity outcomes. Models were fitted in R (v4.2.1) using the lme4 package (v1.1.37). Two-sided Wald z-tests were used to assess regression coefficients. Reported effect sizes represent odds ratios (OR) or adjusted odds ratios (aOR) with 95% confidence intervals (CI). Variables with p-values >0.2 or high collinearity (Pearson correlation coefficient >0.75) were excluded prior to multivariable modelling. Iterative bidirectional step-wise model selection based on Akaike information criterion (AIC) was used to identify best-fit multivariable models. No formal adjustment for multiple comparisons was applied because variables were evaluated within an exploratory AIC-based model selection framework. Perimeter-area ratio measures forest patch shape complexity, with higher values indicating more irregular or fragmented forest patches.
