# Supplementary material for: Filovirus reactive antibodies in wildlife hunting communities suggest ongoing zoonotic spillover in forested Guinea
Source: Nat Commun. 2026 Oct 8;17:9974. doi: 10.1038/s41467-026-77653-3 (PMC13650133; doi:10.1038/s41467-026-77653-3)
Supplement: Supplementary file 2 [file 41467_2026_77653_MOESM2_ESM.pdf]

nature portfolio

Corresponding author(s): Grace Hood, Miles Carroll

Last updated by author(s): May 20, 2026

Reporting Summary

Nature Portfolio wishes to improve the reproducibility of the work that we publish. This form provides structure for consistency and transparency in reporting. For further information on Nature Portfolio policies, see our Editorial Policies and the Editorial Policy Checklist.

Statistics

For all statistical analyses, confirm that the following items are present in the figure legend, table legend, main text, or Methods section.

n/a	Confirmed	
☐	☒	The exact sample size (n) for each experimental group/condition, given as a discrete number and unit of measurement
☐	☒	A statement on whether measurements were taken from distinct samples or whether the same sample was measured repeatedly
☐	☒	The statistical test(s) used AND whether they are one- or two-sided<br>*Only common tests should be described solely by name; describe more complex techniques in the Methods section.*
☐	☒	A description of all covariates tested
☐	☒	A description of any assumptions or corrections, such as tests of normality and adjustment for multiple comparisons
☐	☒	A full description of the statistical parameters including central tendency (e.g. means) or other basic estimates (e.g. regression coefficient) AND variation (e.g. standard deviation) or associated estimates of uncertainty (e.g. confidence intervals)
☐	☒	For null hypothesis testing, the test statistic (e.g. F, t, r) with confidence intervals, effect sizes, degrees of freedom and P value noted<br>*Give P values as exact values whenever suitable.*
☒	☐	For Bayesian analysis, information on the choice of priors and Markov chain Monte Carlo settings
☐	☒	For hierarchical and complex designs, identification of the appropriate level for tests and full reporting of outcomes
☐	☒	Estimates of effect sizes (e.g. Cohen's d, Pearson's r), indicating how they were calculated

Our web collection on statistics for biologists contains articles on many of the points above.

Software and code

Policy information about availability of computer code

Data collection	Code used for statistical analyses was written in R (v4.2.1) using publicly available packages, as described in the methods.
Data analysis	Code used for statistical analyses was written in R (v4.2.1) using publicly available packages, as described in the methods.

For manuscripts utilizing custom algorithms or software that are central to the research but not yet described in published literature, software must be made available to editors and reviewers. We strongly encourage code deposition in a community repository (e.g. GitHub). See the Nature Portfolio guidelines for submitting code & software for further information.

Data

Policy information about availability of data

All manuscripts must include a data availability statement. This statement should provide the following information, where applicable:

- Accession codes, unique identifiers, or web links for publicly available datasets
- A description of any restrictions on data availability
- For clinical datasets or third party data, please ensure that the statement adheres to our policy

The data generated in this study include individual-level demographic, behavioural, serological and geospatial data from human participants and are available under restricted access due to ethical and privacy considerations related to participant confidentiality and the potential identifiability of study communities. De-identified data supporting the findings of this study may be made available to qualified researchers for non-commercial research purposes following approval by the relevant institutional and national ethics committees in Guinea and the United Kingdom. Requests for access should be directed to the corresponding authors, Grace Hood

(grace.hood@well.ox.ac.uk) or Miles Carroll (miles.carroll@ndm.ox.ac.uk), and will be reviewed within approximately 6–8 weeks. The sequence data used in this study are publicly available in GenBank. The accession numbers are as follows: EBOV GP, **NC_002549.1** (https://www.ncbi.nlm.nih.gov/nuccore/NC_002549.1); BDBV GP, **FJ217161.1** (https://www.ncbi.nlm.nih.gov/nuccore/FJ217161.1) and **NC_014373** (https://www.ncbi.nlm.nih.gov/nuccore/NC_014373); TAFV GP, **NC_014372** (https://www.ncbi.nlm.nih.gov/nuccore/NC_014372) and **FJ217162** (https://www.ncbi.nlm.nih.gov/nuccore/FJ217162); BOMV GP, **YP_009513277.1** (https://www.ncbi.nlm.nih.gov/protein/YP_009513277.1) and **MF319185.1** (https://www.ncbi.nlm.nih.gov/nuccore/MF319185.1); SUDV GP, **NC_006432.1** (https://www.ncbi.nlm.nih.gov/nuccore/NC_006432.1) and **FJ968794** (https://www.ncbi.nlm.nih.gov/nuccore/FJ968794); RESTV GP, **AF522874.1** (https://www.ncbi.nlm.nih.gov/nuccore/AF522874.1) and **AY769362** (https://www.ncbi.nlm.nih.gov/nuccore/AY769362); LLOV GP, **NC_016144.1** (https://www.ncbi.nlm.nih.gov/nuccore/NC_016144.1) and **OQ630505.1** (https://www.ncbi.nlm.nih.gov/nuccore/OQ630505.1); MLAV GP, **KX371887.2** (https://www.ncbi.nlm.nih.gov/nuccore/KX371887.2); and EBOV Makona GP, **KJ660347.1** (https://www.ncbi.nlm.nih.gov/nuccore/KJ660347.1).

Research involving human participants, their data, or biological material

Policy information about studies with human participants or human data. See also policy information about sex, gender (identity/presentation), and sexual orientation and race, ethnicity and racism.

Reporting on sex and gender: Sex (biological attribute), as self-reported by participants, are reported in the manuscript.

Reporting on race, ethnicity, or other socially relevant groupings: Race/ethnicity is not reported.

Population characteristics: Covariate-relevant participant characteristics collected through questionnaire surveys included age, sex, occupation, hunting activity, wildlife exposure behaviours, and environmental exposure variables derived from village geolocation data.

Recruitment: Recruitment was performed through community engagement activities and local study teams working in participating villages. Eligible participants included wildlife hunters and close contacts who provided informed consent to participate in questionnaire surveys and blood sample collection. Participants were deliberately recruited from wildlife hunting communities considered at elevated risk of zoonotic exposure, and the study was therefore not designed to be representative of the general population of Guinea.

As participation was voluntary, the study may be subject to self-selection bias. Individuals with increased interest in health-related research may have been more likely to participate, although the direction of this bias is uncertain. For example, individuals may have had increased health-seeking behaviour, greater engagement in preventive practices (decreasing estimates of risk) or have been motivated by prior experiences of illness (increasing estimates of risk). Recall bias and desirability bias may also have affected questionnaire-derived behavioural data, particularly for self-reported hunting practices and wildlife exposures.

To mitigate these limitations, recruitment was conducted across multiple villages and analyses integrated serological, spatial and behavioural datasets to support consistency across independent lines of evidence. The findings should therefore be interpreted within the context of the sampled high-risk wildlife hunting communities.

Ethics oversight: Ethical approval for the study was granted by the board of UK research ethics council, the national research for health ethic committee of Guinea (permit Nº012/CENRS/2017 and Nº135/CENRS/22) and the Oxford Tropical Research Ethics Committee (OxTREC:536-22). Written informed consent was obtained from all participants.

Note that full information on the approval of the study protocol must also be provided in the manuscript.

Field-specific reporting

Please select the one below that is the best fit for your research. If you are not sure, read the appropriate sections before making your selection.

☒ Life sciences ☐ Behavioural & social sciences ☐ Ecological, evolutionary & environmental sciences

For a reference copy of the document with all sections, see nature.com/documents/nr-reporting-summary-flat.pdf

Life sciences study design

All studies must disclose on these points even when the disclosure is negative.

Sample size: No formal statistical sample size calculations were performed prior to the study. Sample sizes were determined by the number of eligible and consenting participants that could be recruited from remote wildlife hunting communities during field campaigns conducted in 2017 and 2023. Recruitment was guided by logistical feasibility, community engagement, field accessibility and the availability of resources in geographically remote regions of Forested Guinea. The study aimed to maximize representation across villages and demographic groups within these high-risk populations. For laboratory analyses, all available serum samples with sufficient remaining volume and metadata were included. Subsets of samples for downstream pseudotyped virus neutralisation assays were selected based on antibody reactivity patterns identified through exploratory K-means clustering analyses.

Data exclusions: No data were excluded from analyses.

Replication: Serological and pseudotyped virus neutralisation assays included technical replicates, appropriate positive and negative controls, and

Replication	standardized analytical pipelines. Some experiments could not be repeated extensively due to the limited volume of human serum samples available from this remote field study. These samples were considered highly valuable and finite, requiring careful prioritisation of downstream analyses. No inconsistencies were observed in attempts to reproduce experimental findings.
Randomization	Participants were not randomly allocated into experimental groups because this was an observational field study rather than an interventional study. For downstream analyses, including pseudotyped virus neutralisation assays, samples were selected based on predefined serological reactivity patterns identified through clustering analyses.<br><br>Covariates were controlled for in statistical modelling. Here, statistical models examining environmental and behavioural risk factors incorporated demographic and environmental covariates, and sampling year was included as a random effect within generalized linear mixed-effects models to account for temporal variation.
Blinding	Not relevant to the study design.

Reporting for specific materials, systems and methods

We require information from authors about some types of materials, experimental systems and methods used in many studies. Here, indicate whether each material, system or method listed is relevant to your study. If you are not sure if a list item applies to your research, read the appropriate section before selecting a response.

Materials & experimental systems

n/a	Involved in the study
☐	☒ Antibodies
☐	☒ Eukaryotic cell lines
☒	☐ Palaeontology and archaeology
☒	☐ Animals and other organisms
☒	☐ Clinical data
☒	☐ Dual use research of concern
☒	☐ Plants

Methods

n/a	Involved in the study
☒	☐ ChIP-seq
☒	☐ Flow cytometry
☒	☐ MRI-based neuroimaging

Antibodies

Antibodies used	A biotinylated cross-adsorbed goat anti-human IgG secondary antibody (Invitrogen, Cat. No. A18815; diluted 1:5000) was used for detection of human IgG responses in multiplex microsphere immunoassays.
Validation	Antibody validation was based on manufacturer-provided quality control and validation data demonstrating reactivity against human IgG and cross-adsorption to minimize species cross-reactivity, according to the manufacturer's datasheet. No additional in-house antibody validation was performed.

Eukaryotic cell lines

Policy information about cell lines and Sex and Gender in Research

Cell line source(s)	HEK293T cells (human embryonic kidney cells) were obtained from the American Type Culture Collection (ATCC, CRL-3216).
Authentication	The cell line was not authenticated in-house following purchase.
Mycoplasma contamination	All cell lines tested negative for Mycoplasma contamination.
Commonly misidentified lines (See ICLAC register)	HEK293T/17 cells (ATCC CRL-11268) were obtained from ATCC and are not listed on the International Cell Line Authentication Committee (ICLAC) Register of Misidentified Cell Lines.

Plants

Seed stocks	*Report on the source of all seed stocks or other plant material used. If applicable, state the seed stock centre and catalogue number. If plant specimens were collected from the field, describe the collection location, date and sampling procedures.*
Novel plant genotypes	*Describe the methods by which all novel plant genotypes were produced. This includes those generated by transgenic approaches, gene editing, chemical/radiation-based mutagenesis and hybridization. For transgenic lines, describe the transformation method, the number of independent lines analyzed and the generation upon which experiments were performed. For gene-edited lines, describe the editor used, the endogenous sequence targeted for editing, the targeting guide RNA sequence (if applicable) and how the editor was applied.*
Authentication	*Describe any authentication procedures for each seed stock used or novel genotype generated. Describe any experiments used to assess the effect of a mutation and, where applicable, how potential secondary effects (e.g. second site T-DNA insertions, mosiacism, off-target gene editing) were examined.*
