# Supplementary material for: Filovirus reactive antibodies in wildlife hunting communities suggest ongoing zoonotic spillover in forested Guinea
Source: Nat Commun. 2026 Oct 8;17:9974. doi: 10.1038/s41467-026-77653-3 (PMC13650133; doi:10.1038/s41467-026-77653-3)
Supplement: Supplementary file 3 [file 41467_2026_77653_MOESM3_ESM.pdf]

nature portfolio

Peer Review File

Filovirus Reactive Antibodies in Wildlife Hunting Communities suggest Ongoing Zoonotic Spillover in Forested Guinea

Corresponding Author: Dr Grace Hood

This file contains all reviewer reports in order by version, followed by all author rebuttals in order by version.

Version 0:

Reviewer comments:

Reviewer #1

(Remarks to the Author)
Hood et al., Nat Commun
"Filovirus-reactive antibodies in wildlife hunting communities, Forested Guinea; evidence of ongoing zoonotic spillover events"

Filoviruses are zoonotic pathogens that can be transmitted from animals to humans, leading to serious public health concerns. Here, the authors conducted a longitudinal study to investigate filovirus exposure in wildlife hunting communities in Forested Guinea through serological surveillance combined with environmental risk factor analysis. They collected serum samples from 511 participants in 2017 and 648 in 2023, and measured levels of viral GP-specific antibodies against multiple filoviruses by using multiplex immunoassays, followed by pseudotyped neutralization assays. Unsupervised clustering revealed individuals with antibody responses to multiple filovirus species beyond EBOV, with neutralisation assays demonstrating both species-specific and cross-reactive responses. Integration of satellite-derived land-cover data with serological findings identified associations between environmental factors such as deforestation and infection risk for different filoviruses. This type of study is important for understanding spillover mechanisms and to inform surveillance strategies in high-risk regions. However, several issues regarding methodological clarity and data interpretation need to be addressed.

Major points:

1. Figure 2 shows Cluster 5 with high antibody titers against both BDBV and EBOV, but the authors do not interpretate these findings in either the Results or Discussion. The authors should clarify whether this represents dual exposure to both BDBV and EBOV, cross-reactivity, or infection with an antigenically intermediate virus.
2. The authors do not specify how many of the 511 (2017) and 648 (2023) participants had confirmed prior history of EBOV infection. This is a fundamental methodological issue that affects interpretation of all serological results. Were participants screened for previous EBOV infection? If so, how many had confirmed infection? If not, this should be clearly stated as a limitation.
3. In Figure 4, some of participants showed higher neutralizing antibodies compared to EBOV survivors (e.g., JAB371, JAB289, JAB377), but critical information is missing including the sampling year (2017 vs 2023), village of residence, and their history of previous EBOV infection. This information is important for interpreting whether the observed neutralizing antibodies represents 2013–2016 outbreak-related infection or other exposure events.
4. In Figure 5, some individuals showed maintained or increased IgG titers between 2017 and 2023, but the authors do not adequately consider re-infection as a confounding factor in their antibody half-life calculations. This could substantially affect the interpretation of "ongoing spillover" versus "antibody boosting".
5. On page 17, line 325: The claim that seroconversion to LLOV (or a related virus) indicates "circulation of an antigenically related cuevavirus" is based on extremely limited data (1/8 individuals showing stronger LLOV neutralization). This small subset raises serious concerns about statistical validity and biological significance.
6. Lines 292–296: It seems the authors believe exposure was limited to BDBV-like viruses, but based on the data presented, this reviewer has concerns that exposure to EBOV through contact with bush meat cannot be ruled out. Since the GP proteins of filoviruses are not highly similar, it is unlikely that cross-reactive antibodies would be easily produced, and

exposure to multiple viruses may have occurred through contact with bush meat or other sources.

Minor points:
1. "Error! Reference source not found" appears throughout the manuscript (lines 138, 148, 173, 175, 207).
2. In Table 1, the Guinea 2017 cohort has a total number of participants of 511, divided into 228 males and 223 females. However, there are 60 remaining participants not recorded. Could you explain the missing data? Also, it seems that Table 1 contains calculation errors (228/511 = 45%, not 56% for males). In the "Wildlife hunting" section, for 2017 cohort there are 278 hunters and 232 non-hunters, which adds up to 510. There is 1 missing from the total of 511 participants. Similarly, for the 2023 cohort, there is 1 missing participant.
3. On page 4, line 75: It is stated that the wildlife hunters (all male) constituted 54% of the study population, with the remaining 46% reporting close household contact with a hunter. However, Table 1 states that the wildlife hunters constituted 55%. The data must be presented accurately and consistently.
4. In Figure 4, the EBOV survivor pool shows relatively modest neutralization titers. Since they are confirmed survivors, the authors should comment on this.
5. The authors should specify which figure supports the content of the text. For example, lines 161–180 and 175–180 lack figure references.
6. Figure 2: The colors are similar (e.g., BDBV and TAFV), making it difficult to distinguish between them. The coloring should be adjusted.
7. The text in Figure 3 is too small, making it hard to grasp both the figure's content and the related paragraphs (lines 132–154). This figure needs to be adjusted.
8. Inconsistent terminology ("nonhuman primates" vs "non-human primates"), grammatical errors ("sera was incubated" should be "sera were incubated") (page 23, line 518), and incomplete supplementary references need to be corrected (page 14, line 232). "[IQR: 4 – 15)" should be replaced with "[IQR: 4 – 15]" (page 5, line 97).
9. On page 22, lines 461–474 "Pseudotyped virus neutralisation assay (PVNA)": Please specify the luciferase substrate used and its incubation time before reading. For NT50 Calculation Validity, the statement "Hill-Slope constraint of less than 0" is biologically implausible for a neutralisation curve (the slope should be positive). This likely reflects a typo or misinterpretation of software settings. Please clarify or correct this. Please use the temperature units consistently (e.g., "37°C" instead of "37 degrees"). Please spell out "TCID50" at the first mention. Please clarify or spell out "DMEM 1%FBS/1%P/1%S" as "DMEM supplemented with 1% fetal bovine serum and 1% penicillin-streptomycin".
10. On pages 23–24, lines 515–529 "Multiplex microsphere-based immunoassay": For antigen-coupled microspheres, please specify which filovirus envelope proteins (e.g., Ebola GP, Marburg GP) were used as antigens. Omitting this information limits reproducibility and interpretation of cross-reactivity. Please define "room temperature" numerically (e.g., 22–25°C) to ensure consistency across experiments.

Reviewer #2

(Remarks to the Author)
General Comments

This study investigates serological evidence of filovirus exposure in a wildlife hunter cohort in Guinea. I appreciate that the authors included two negative control cohorts (Negative cohort 2015 and 2017, Coyah and Guéckédou; Blood donor cohort, 2020, UK), which strengthen the study design. The detailed questionnaire on wildlife hunting practices adds valuable context, clearly demonstrating that most respondents not only consumed bushmeat but also handled, washed, or prepared carcasses, with documented hunting of bats.

Previous work has shown that orthoebolaviruses are highly cross-reactive and often indistinguishable serologically, particularly EBOV–BDBV–TAFV and SUDV–RESTV. Cross-reactive binding antibody responses were demonstrated in three of the fifteen identified clusters; however, the remaining clusters consisted of individuals with binding antibody responses against only a single filovirus, which is unexpected given the known cross-reactivity. The BDBV cluster presented in Figure 2 is a notable finding, as five of the thirteen individuals in this cluster were able to neutralize BDBV glycoprotein pseudovirus and showed persistent responses in the 2023 samples. However, these filovirus pseudovirus neutralization assays have not been validated against live filovirus neutralization assays, and it is well known that pseudovirus assays can overestimate virus neutralization.

The authors should consider analyzing individuals separately and presenting individual results. For specific individuals, it would be helpful to see both binding antibody and pseudovirus neutralization responses across the panel of filoviruses to better assess true seropositivity and patterns of cross-reactivity. Finally, I suggest that the manuscript's discussion of individual filoviruses and the attempts to link exposure to specific filoviruses with particular land-use changes may be somewhat premature, especially given the acknowledged cross-reactivity (lines 365–366). I believe that such detailed conclusions would be better supported if a specific virus were confirmed in the region through PCR and/or virus isolation.

Specific Comments

Abstract: The list of viruses includes filoviruses, not species of Filoviridae. It should read "filoviruses."

Line 15: Consider adding a cited estimate for deforestation in Guinea to support this statement.

Line 41: There has only been one reported human case of TAFV infection. Recommend revising the statement to reflect that

there is insufficient evidence to claim it causes milder disease. Also, change “asymptomatic human disease” to “asymptomatic infection.”

Lines 85–86: Revise to the current nomenclature: “Ebola Zaire” should be referred to as “Ebola virus.”

Lines 146–149: Clarify whether the PCR-confirmed EBOV survivor serum samples were run side-by-side with these specimens, and if so, please include these results in the corresponding figure.

Lines 189: Consider revising the subtitle to: “Durable EBOV antibody responses, six years post-2013–16 West African EBOV epidemic.” We don’t know if these individuals are “immune”.

Figure 3: The figure is too difficult to see as presented. I was not able to review.

Lines 276–277: Emphasize that this is a pseudovirus neutralization assay, not a live virus assay.

Line 298: Clarify whether the positive association between primate hunting and anti-BDBV responses was statistically significant in the multivariate analysis.

Line 372: Unless there is a direct comparison with live virus neutralization, I feel the filovirus pseudovirus neutralization assays should not be described as a well-established surrogate.

Line 461: Specify the titration range of the three-fold dilutions.

Reviewer #3

(Remarks to the Author)
Major
This manuscript by Hood et al. investigates an interesting and important topic: the potential undetected exposure of individuals to different filoviruses and sheds new light on this topic. The manuscript is well written and having paired follow-up examples is a great advantage compared to other seroprevalence studies. Also, the broad antigen panel is that is used, is interesting. The major downside of the manuscript is that it explores too many different questions (hunting practices, antibody profiles, association with land-cover) and fails to go into depth. The many objectives stand in the way of a cohesive and convincing story. I wonder if a manuscript focused on BDBV seroconversion, with some additional triangulation that corroborates that this was really exposure to BDBV would not be the better option.

Mahor concerns:
- My main comment is that I’m not yet convinced of the main conclusion that the signals picked up by serology represent an exposure to the filovirus in question. The control population, which is used to determine the cut-offs of the assay are UK residents. However, in my opinion, this is not a good reference population. The issue with serology in malaria-endemic settings is that repeated malaria exposure causes a polyclonal gamma expansion, leading to aspecific binding of antibodies and a higher rate of false-positive results on serology. The specificity of serological assays, therefore, changes in these settings. I’m not sure if the neutralizing antibody assay used here do not also exhibit this potential aspecific binding to some degree. Could any of the following things be done to exclude this issue: use a (larger) cohort of controls from malaria endemic areas as controls rather than the UK population, check if aspecific binding to other pathogens that are not endemic also occurs, check for malaria antibodies or total IgG and see if it correlates with filovirus antibodies… I understand that it is not possible to do all of this, but some additional steps should be taken to ensure that this is not a specific binding. Supplementary figure 10 is revealing in this regard. The distribution of antibody profiles completely overlap here between hunters and non-hunters.
- An argument against a specific binding is the fact that the antibody profiles in Figure 1 may exhibit a bimodal distribution, with the second peak corresponding to real exposure. I think Figure 1 can therefore benefit from adding the population of EVD-confirmed individuals to see how the distribution of antibodies from infected individuals shapes and compares to the other populations.
- In addition, I think the manuscript could benefit from a statistical analysis which explores in how far these population differ from each other
- Line 55: the authors write: ‘ongoing seroconversion’. However, there is no data in the manuscript on those participants who were seronegative at the beginning of the study, but seropositive at the end. They only provide information on those with decline antibodies. Nevertheless, a more in-depth characterization of those that seroconvert looks to me most interesting. What is their profile, do they cluster geographically, did they recall any illness?
- Line 73: Can the others give details on how many individuals were approached, but refused participation, and add numbers on the size of the villages to estimate how representative the study population is.
- Line 94 onwards: I would avoid the term bushmeat as is done elsewhere in the manuscript, as it has a stigmatizing connotation.
- Line 94 onwards: in this paragraph, only parts of the result are described in a narrative paragraph. While the section is well written, the authors should include a table with all the data, including all the questions asked and the proportions of the different answers. Otherwise, it is difficult for the reader to interpret the data correctly.
- Line 138: I’m not convinced by the relevance of the machine learning algorithm. The K-means clustering will provide an output based on the number of predefined clusters. However, the authors do not provide data that validates these clusters, so it is unsure if they have any biological meaning.

- What is the rationale for choosing 15 clusters (k = 15)? Does not
- Line 146: According to the authors based on the antibody profile 57 individuals were 'likely EBOV exposed'. Would this not provide an opportunity to biologically validate at least these 3 clusters? Is there data in the questionnaire that indicate that have ever been in contact with an EVD patient, or were from villages affected by EVD. If they have antibodies, they were not just exposed, but infected. This would mean that all these individuals had an asymptomatic EVD infection, which seems unlikely.
- Line 162: 'antibody profiles consistent with exposure to …': I find this wording a bit misleading, as we don't have validated antibody profiles of individuals with a confirmed infection with these viruses.
- Line 168: the authors write: '5 individuals neutralized BDBV more strongly' However, the 95% CI's in figure 4 completely overlap. This statement therefor does not seem to be correct. Are the 95% CI calculated based on sample replicates? Is there not a way of comparing the antibody mean of the log antibody titers between the different clustering groups. Similarly for the paragraph starting from line 174.
- Line 168: five out of how many?
- Line 171: Does this data contradict the fact that these antibody profiles correspond to exposure to BDBV?
- Line 180: What is the rationale of not showing these data. Are other clusters also evaluated using neutralizing assays? Again, a more systematic validation of these clusters is needed.
- Line 206: In this paragraph, the authors explore differences between villages. However, in order to interpret this data it is necessary to know the number of participants per village. There is substantial heterogeneity; however, the overall number of participants is low compared to the number of villages. Already, having only 10-20 participants per village, leads to random effects between villages. However, maybe for some villages only a few individuals were recruited. This information is needed.
- Line 208: According to Figure 6, villages are anonymized, which I think is a good idea. However, in line 208, the so-called hotspot name is disclosed. I would avoid this.
- In general, were participants asked if they were ever diagnosed with EVD?
- Fig 7: Heatmaps are often difficult to interpret, which is especially the case here. I wonder if a graph would be more suitable. It is difficult to interpret the color changes of time.
- Line 222: the authors describe multivariate models, but provide no information on which parameters were included in the models. They only describe two significant results.
- Line 231: Did these populations differ? You state so in the discussion, but I mainly see overlap. Are there any statistical test done?
- Line 239: The authors here test the association of 3 different parameters (coverage, shape index, perimeter area ratio), and this for an unknown number of land cover types (three, forest, built area?). They test the association with 5 different antigens. It is unclear from the manuscript how many associations were tested, as the authors only display the significant results. Moreover, from the table it appears to be done on different scale. This would lead to serious of multiple testing. The authors should state which variables exactly were tested, why and also display negative results.
- Line 288; in the discussion the authors give 'Individual-level' epidemiological data to further support their hypothesis. However, they should do so in the results.

Minor:
- The introduction could benefit from a brief recap of filovirus taxonomy
- Line 6: I would use a more nuanced word than hotspot
- Line 15-18 This is a complicated sentence which is difficult to interprate
- Line 22: What do the authors mean with 'more proximal' drivers?
- Line 55: This part seems more fit for a discussion
- Line 71: Who collected the data
- Figure 4: As data are probably pseudonimized, it's better not to disclose study numbers in the legend of the figure. It makes the interpretation of this figure also more complex
- There is an issue with referencing to the figures: all throughout the text it indicates Error! Reference source not found): which complicates the peer review

Reviewer #4

(Remarks to the Author)
This manuscript reports a serological survey of filovirus exposure in rural hunter communities in forested Guinea, with sampling conducted in 2017 (previously published?) and 2023. The study employs a multiplex microsphere-based immunoassay (MMIA) to detect IgG responses to nine filovirus glycoproteins, along with pseudo-typed virus neutralization assays for functional assessment. The authors also integrate environmental and behavioural risk factor analysis, including land use and hunting activities. The central finding is the detection of binding antibodies against multiple filovirus species beyond Ebola virus (EBOV), including BDBV, TAFV, BOMV, LLOV, MLAV, and RESTV, interpreted as evidence for ongoing zoonotic spillover.
This work addresses an important public health question regarding filovirus ecology. However, interpretation of the serological data is limited by well-known issues of broad cross-reactivity among filoviruses, particularly among Ortho-ebolaviruses. Additional clarity and a more cautious interpretation are warranted, especially regarding the clustering approach and risk factor analysis.

Major comments

• Previously published data (2017 cohort) ?

It appears from the Introduction and references (see line 50 [adjust as needed]) that the 2017 wildlife hunter cohort included in this study is substantially or entirely the same as that studied and published previously by Akoi Boré et al., Nature Communications, 2024.
Reference : Akoi Boré, J., et al., Serological evidence of zoonotic filovirus exposure among bushmeat hunters in Guinea. Nature Communications, 2024) (499 bushmeat hunters in Macenta Prefecture collected in 2017).
However, this overlap is not communicated with sufficient clarity or transparency in the current manuscript. While the Introduction states, "[45]. In this study, we return to Forested Guinea to resample and expand the wildlife hunter cohort 6 years later...", the Methods only reference the previous article in the context of blood processing ("Blood processing was performed as described in Akoi Boré et al., 2024 [45]."), rather than explicitly stating the source, characteristics, and degree of overlap with the previously published dataset. For clear attribution and to distinguish what is novel, the manuscript should state explicitly that the 2017 cohort is previously published. This is essential for transparency and to avoid redundant reporting.

• Interpretation of serological cross-reactivity and attribution to filovirus species :
Filoviruses, especially within the Ortho-ebolavirus group, are well recognized to induce broad cross-reactive antibody responses. While the authors acknowledge this, seropositivity based primarily on glycoprotein reactivity is particularly prone to overestimation and misattribution of species. The repeated interpretation of seropositivity as definitive evidence of exposure to multiple, including highly geographically restricted, filovirus species (e.g., Lloviu virus, so far detected only in European bats, and Reston virus) is not sufficiently justified given these cross-reactivities.

• K-means Clustering interpretation
The use of unsupervised K-means clustering to define serological “exposure profiles” in the absence of clear serological cutoffs is innovative, but problematic in its current implementation. The authors describe partitioning the data into 15 clusters; this number seems excessive and potentially not biologically meaningful. The method for selection of the number (e.g., AIC, gap statistic, silhouette width, etc.) should be fully described, with supporting criteria/plots included as supplementary material. L146-154 the manuscript describes eight clusters with apparently interpretable patterns (clusters assigned to EBOV, BDBV, etc.), but provides no information about the remaining clusters nor what constitutes a “negative” cluster. For example, cluster 5 (with high MFI for both BDBV and EBOV) is neither described nor considered. This incomplete reporting undermines the risk factor analyses, which are all based on cluster assignments.

• Specificity of PV Neutralization Assay:
The specificity of the pseudotyped virus (PV) neutralization assay is unclear. Results from the EBOV survivor pool showed no significant difference between neutralization of EBOV and BDBV (or TAFV), suggesting that the PV neutralization assay itself may be subject to substantial cross-reactivity. Could the authors clarify the assay's specificity and provide evidence for its capacity to discriminate species-specific neutralizing antibodies within the orthobolaviruses?
Additionally, are the glycoprotein antigens used in the pseudotyped virus production identical to those used in the multiplex microsphere-based immunoassay (MMIA)?

• PV Neutralization Assay Results interpretation
The authors should be cautious as well with the interpretation of the PV Neutralization Assay Results. The neutralization data are interpreted as supporting species-specific exposures in several cases and authors repeatedly wrote that neutralisation for BDBV (or TAFV) was stronger than for EBOV for select individuals, but in most cases, NT50 confidence intervals overlap (see Figure 4a/b), which is indeed mentioned by the authors for TAFV neutralisation but not for BDBV. What statistical comparisons were performed? Are differences between neutralization curves actually significant? Authors should use appropriate paired statistical tests where possible, and interpret results conservatively when confidence intervals are overlapping. Specific individuals are highlighted as putatively BDBV-exposed, yet displayed overlapping neutralization results (For instance, JAB376 is interpreted by the authors as “Individuals who showed stronger neutralisation of BDBV than EBOV in parallel lentiviral pseudotyped virus neutralized tests” in Figure 7 whereas there is no clear difference in figure 4a). The neutralization assay’s resolution should be addressed and limitations made explicit.
Moreover, the PV neutralization data are limited to paired comparisons of a single filovirus (BDBV, TAFV, or LLOV) versus EBOV for each sample. Were these samples also tested for neutralization against the other filoviruses in the panel? If so, it would strengthen the analysis to present comparative neutralization data for everyone across all tested viruses, rather than only in relation to EBOV.

• Hunter/Non-hunter
L355-356 “Further, overall population-level differences could still be seen between hunters and their close contacts, potentially highlighting the additional risks of increased time in the forest or exposure to fresh bodily fluids.”
While the title and discussion emphasize wildlife hunting as a driver of exposure, the only reported group comparison is a supplementary figure showing MFI distributions. No odds ratios for hunting status are provided. Most of the analysis is based solely on the BDBV cluster (for which only NHP consumption and male sex are reported as significant predictors). A full table showing all major risk factors (including bushmeat consumption, hunting, sex, age, etc.) and their statistical associations with all filovirus seropositivity clusters should be included, along with clear reporting on sample sizes for each comparison. This is essential both to address the stated research question and to support conclusions regarding risk factors.
The authors at times use “hunter cohorts,” “wildlife hunters,” and “study population” interchangeably. Yet Table 1 makes clear that both hunters and their close contacts (non-hunters) are included, however most figures are described to include “wildlife hunters” but appear to include the entire study population (Figures 1 and 3 include both hunter and non-hunter groups, as per Table 1). It is unclear for Figure 6 “amongst Guinean Wildlife hunters” whether the authors only include mean MFI from hunters in this figure or if it is a misuse? This should be clarified throughout the text and in figure legends/titles, as should any analysis restricted to hunters or resampled individuals.

• Resampling
For resampling: L74 mentions 256 individuals, but only 12 dots are depicted in Figure 5—please clarify how many individuals were longitudinally resampled and are analyzed for antibody durability or new exposure. What is the percentage of resampled individuals which are still considered seropositive ?
The authors state they have demonstrated "ongoing seroconversion." Please clarify which results directly support this assertion. Specifically, what is the rate or percentage of seroconversion observed among re-sampled individuals between 2017 and 2023, and how is this determined?

• EBOV serology
Authors concluded on L274 taht they demonstrated “broad and durable B-cell mediated antibody response” to EBOV following the 2013-2016 outbreak. However, this claim should be more cautiously stated, as the study was not designed as a follow-up of confirmed EBOV survivors but rather sampled a mixed population from several villages. While the presence of reactive antibodies to EBOV glycoprotein is shown, the exact percentage of seropositive individuals within the population is not reported, nor are results stratified according to whether villages were affected or unaffected by the outbreak. Figure 6, which presents MFI values by village, is interesting but authors only commented on BDBV MFI and did not discuss EBOV results. Some villages show high EBOV MFI mean despite being unaffected (e.g., villages 4 and 26 in 2017, and villages 4, 35, 42, and 43 in 2023).

Minor comments
- Clarify whether “hunter,” “wildlife hunter,” or “study population” refers to all participants or only a subgroup, in figures, legends, and Results text.

- In L73 and in Table 1, authors should mention that 2017 cohort was previously published by Akoi Boré et al., Nature Communications, 2024.

- OR
Univariable and multivariable are mentioned by the authors L233, but which OR are in the following sentences (OR of BDBV cluster seropositivity for non-human primate consumption and gender) ? When presenting risk factor odds ratios, specify whether they derive from univariable or multivariable models. Consider providing a supplementary table showing ORs (with 95% CIs) for all tested risk factors and each serological outcome.
- Village numbering
The presentation of village-level data (Figure 6) is unclear: are village numbers consistent between 2017 and 2023 ? (this doesn’t seem the case based on the affected/unaffected status). Keeping the same numbers in both panel would help interpretation. Moreover, since village 4 is named Maroua at one point, the sentence “Villages are anonymized” seems unnecessary. Authors can simply mention that villages are numbered.
- Authors should mention the exact number of samples tested at each step in the analyses:
Line 162–163: “Individuals with antibody profiles consistent with exposure to BDBV, TAFV, BOMV, RESTV and LLOV were then assessed for their ability to neutralize the filovirus...” ; Line 168: “Of those tested from the BDBV cluster...” ; Line 174: “Of the individuals tested from the TAFV cluster...”
Please specify the number of samples from each cluster that were tested in the neutralization assay.

Line 190–193: "For individuals with detectable EBOV antibodies in 2017, a comparison of 2017 to 2023 demonstrated the population-level durability of anti-EBOV antibodies across all villages. Antibody decay rates were modeled as an exponential decrease from time of infection, with a regression model fitted to IgG antibody data from each individual above a seropositivity threshold for EBOV in 2017 who was resampled in 2023." Please specify how many were resampled and included in the analyses, in the methods, results and the legends of figure 5.

- On line 100, the statement “Bat exposure and hunting was reported to be highly seasonal, with 87% (292/334) of respondents seeing them or hunting them (92%, 122/133) at the start of the dry season” is unclear with respect to the denominators for each percentage. Please clarify the exact numbers and corresponding percentages for each response group

Version 1:

Reviewer comments:

Reviewer #1

(Remarks to the Author)
The authors have addressed all of my major and minor concerns with care and transparency. The addition of supplementary materials, clarification of methodological approaches, and more cautious interpretation of the results have substantially strengthened the manuscript. The study provides important longitudinal serological evidence of potential filovirus exposure in wildlife hunting communities in Guinea, with appropriate acknowledgment of its limitations.

Reviewer #2

(Remarks to the Author)
Thank you for your revisions. All of my previous comments have been addressed.

Reviewer #3

(Remarks to the Author)
The authors have responded adequatly to most of my concerns.

Open Access This Peer Review File is licensed under a Creative Commons Attribution 4.0 International License, which permits use, sharing, adaptation, distribution and reproduction in any medium or format, as long as you give appropriate credit to the original author(s) and the source, provide a link to the Creative Commons license, and indicate if changes were made.
In cases where reviewers are anonymous, credit should be given to 'Anonymous Referee' and the source.
The images or other third party material in this Peer Review File are included in the article's Creative Commons license, unless indicated otherwise in a credit line to the material. If material is not included in the article's Creative Commons license and your intended use is not permitted by statutory regulation or exceeds the permitted use, you will need to obtain permission directly from the copyright holder.
To view a copy of this license, visit https://creativecommons.org/licenses/by/4.0/

POINT-BY-POINT RESPONSE TO REVIEWERS' COMMENTS

Reviewer #1 (Remarks to the Author):

Hood et al., Nat Commun
"Filovirus-reactive antibodies in wildlife hunting communities, Forested Guinea; evidence of ongoing zoonotic spillover events"

Filoviruses are zoonotic pathogens that can be transmitted from animals to humans, leading to serious public health concerns. Here, the authors conducted a longitudinal study to investigate filovirus exposure in wildlife hunting communities in Forested Guinea through serological surveillance combined with environmental risk factor analysis. They collected serum samples from 511 participants in 2017 and 648 in 2023, and measured levels of viral GP-specific antibodies against multiple filoviruses by using multiplex immunoassays, followed by pseudotyped neutralization assays. Unsupervised clustering revealed individuals with antibody responses to multiple filovirus species beyond EBOV, with neutralisation assays demonstrating both species-specific and cross-reactive responses. Integration of satellite-derived land-cover data with serological findings identified associations between environmental factors such as deforestation and infection risk for different filoviruses. This type of study is important for understanding spillover mechanisms and to inform surveillance strategies in high-risk regions. However, several issues regarding methodological clarity and data interpretation need to be addressed.

We thank the reviewer for their careful and constructive evaluation of our manuscript and for recognising the importance of longitudinal serological surveillance in high-risk, hard-to-reach communities. We have sought to address all comments in detail and have substantially revised the manuscript to improve clarity, transparency, and interpretation.

Major points:

1. Figure 2 shows Cluster 5 with high antibody titers against both BDBV and EBOV, but the authors do not interpretate these findings in either the Results or Discussion. The authors should clarify whether this represents dual exposure to both BDBV and EBOV, cross-reactivity, or infection with an antigenically intermediate virus.

Thank you for bringing this to our attention. The unsupervised clustering algorithm cannot statistically distinguish whether this represents (i) dual exposure to both BDBV and EBOV (ii) cross-reactivity, or (iii) infection with an antigenically intermediate virus. We consider (i) dual exposure to both EBOV and BDBV in a high proportion of individuals (n=13) to be epidemiologically unlikely. We also consider (ii) cross-reactivity following EBOV infection to less likely, as EBOV survivors typically demonstrate cross-reactivity against not only BDBV, but against other more distantly related filoviruses in this assay (1). I present the individual MMIA responses from Cluster 5 (left) in comparison to PCR-confirmed EBOV survivors (right) below to demonstrate the difference. Based on this, we consider (iii) exposure to a virus that is antigenically equidistant from EBOV and BDBV to be the most likely explanation, and have added an interpretation and discussion of these findings to the manuscript.

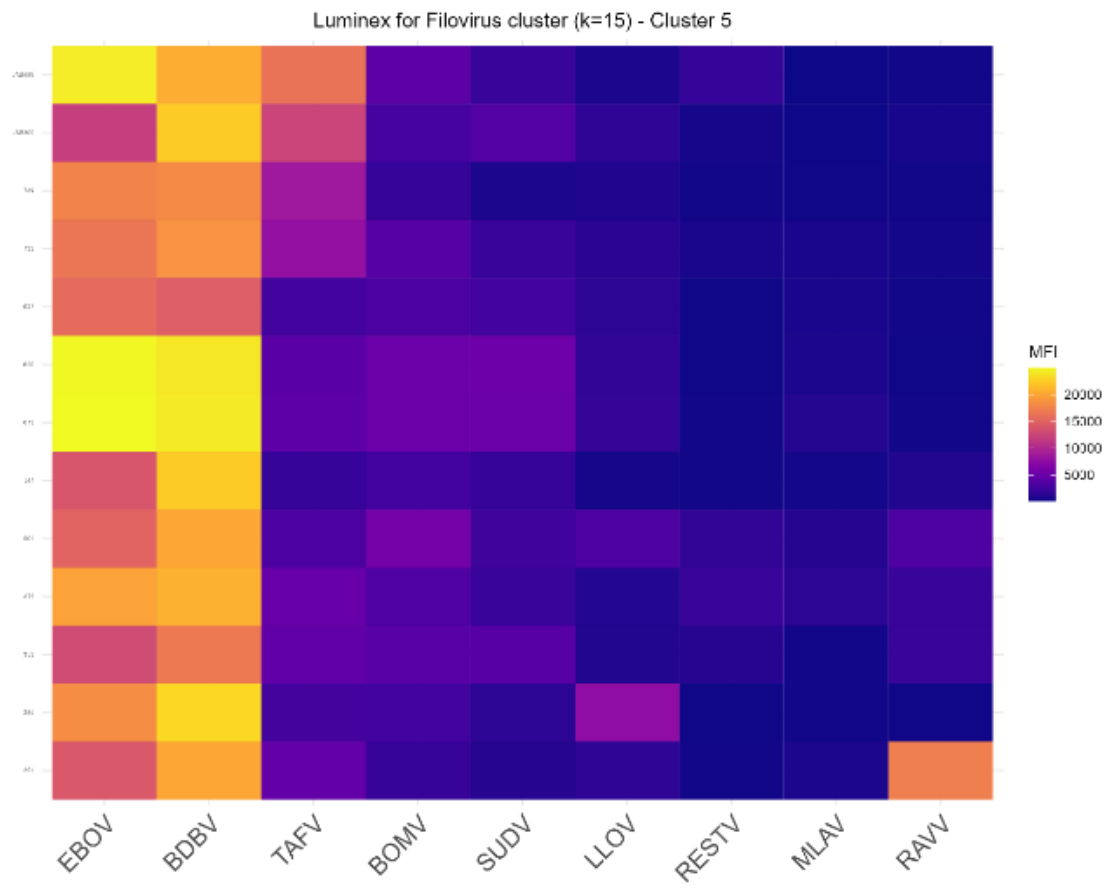

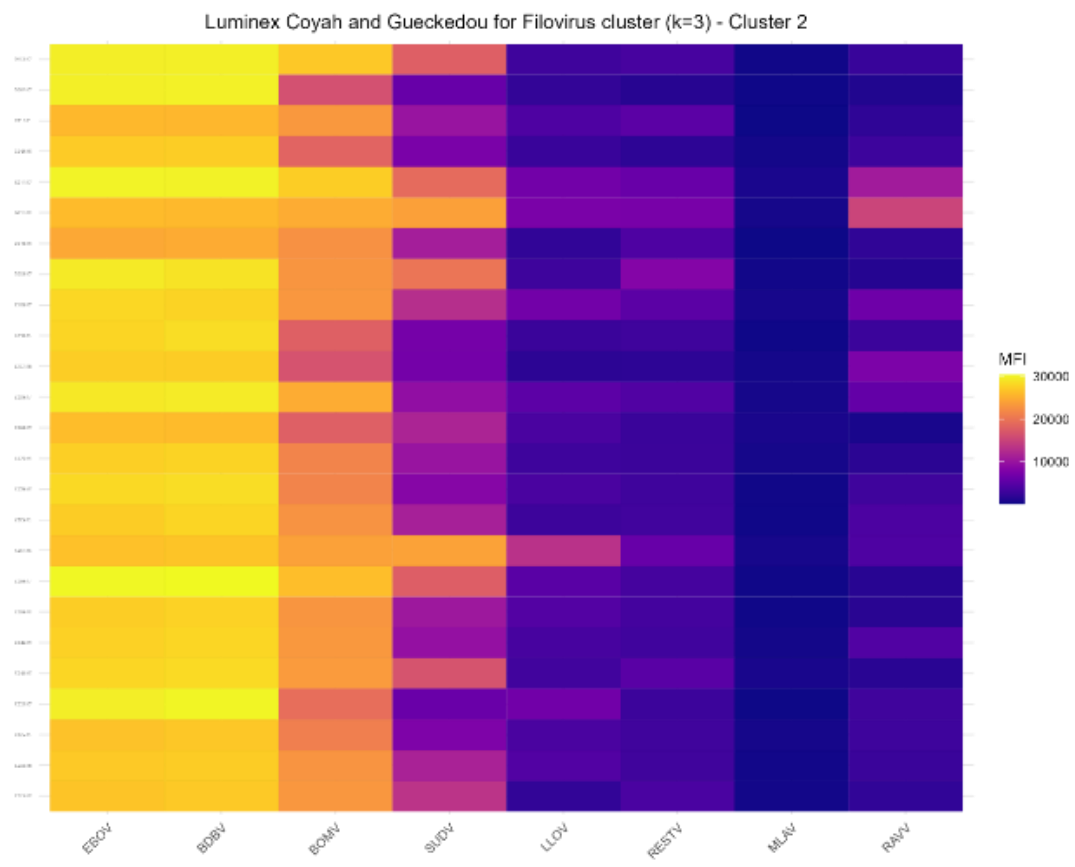

2. The authors do not specify how many of the 511 (2017) and 648 (2023) participants had confirmed prior history of EBOV infection. This is a fundamental methodological issue that affects interpretation of all serological results. Were participants screened for previous EBOV infection? If so, how many had confirmed infection? If not, this should be clearly stated as a limitation.

Unfortunately, we do not have prior PCR-confirmed infection history for EBOV participants and we have included additional wording to acknowledge that this is a limitation of the study (line 295). As is typical for population-based studies conducted in the region after outbreak resolution, retrospective confirmation of EBOV infection by PCR is not feasible. Health prefectures in Forested Guinea did not issue formal “Ebola survivor certificates,” and self-reported histories of suspected EBOV infection were not collected due to concerns regarding recall bias, social stigma, limited access to healthcare during the outbreak period, and clinical overlap with other endemic febrile illnesses such as malaria and typhoid fever. Further, due to the significant stigma and negative associations regarding Ebola research in the area, our local collaborators strongly advised against asking questions regarding Ebola history in order to avoid misunderstandings with study participants.

Consequently, screening for previous EBOV infection would relies on serological screening (e.g., anti-EBOV binding antibodies) which were part of our analysis, which included EBOV-GP in our MMIA panel alongside all other filovirus GPs.

3. In Figure 4, some of participants showed higher neutralizing antibodies compared to EBOV survivors (e.g., JAB371, JAB289, JAB377), but critical information is missing including the sampling year (2017 vs 2023), village of residence, and their history of previous EBOV infection. This information is important for interpreting whether the observed neutralizing antibodies represents 2013–2016 outbreak-related infection or other exposure events.

We agree that additional contextual information is important for interpreting the neutralisation data shown in Figure 4. As recommended, we have added supplementary tables (Supplementary Table 7, Table 8) providing the sampling year (2017 or 2023) and village of residence. As discussed, history of previous EBOV infection is unfortunately, unavailable.

4. In Figure 5, some individuals showed maintained or increased IgG titers between 2017 and 2023, but the authors do not adequately consider re-infection as a confounding factor in their antibody half-life calculations. This could substantially affect the interpretation of “ongoing spillover” versus “antibody boosting”.

We agree that re-infection and subsequent antibody boosting could confound interpretation of antibody durability between 2017 and 2023. As only two longitudinal sampling time points were available, it was not possible to model re-infection or antibody boosting explicitly. We have therefore revised the relevant text (underlined) accordingly (line 208): Based on the population average of these models, the EBOV IgG antibody half-life was estimated as 7.5 years (Figure 6), though the possibility of re-infection and subsequent antibody boosting could not be excluded.

5. On page 17, line 325: The claim that seroconversion to LLOV (or a related virus) indicates "circulation of an antigenically related cuevavirus" is based on extremely limited data (1/8 individuals showing stronger LLOV neutralization). This small subset raises serious concerns about statistical validity and biological significance.

We agree with the reviewer that the original wording overstated the significance of the LLOV findings and have revised this wording (underlined) to adopt a more cautious interpretation (line 351):

Unlike other filoviruses, LLOV is non-endemic to the region, and only 1/8 individuals were found to exhibit more potent neutralisation of LLOV (compared to EBOV). This may be interpreted cautiously as preliminary evidence for the circulation of an antigenically related cuevavirus in the region (rather than the circulation of the LLOV species, yet to be identified outside of Europe).

We have added an additional supplementary figure demonstrating the magnitude of the seroconversion to LLOV in these two individuals for transparency (Supplementary, Figure 11).

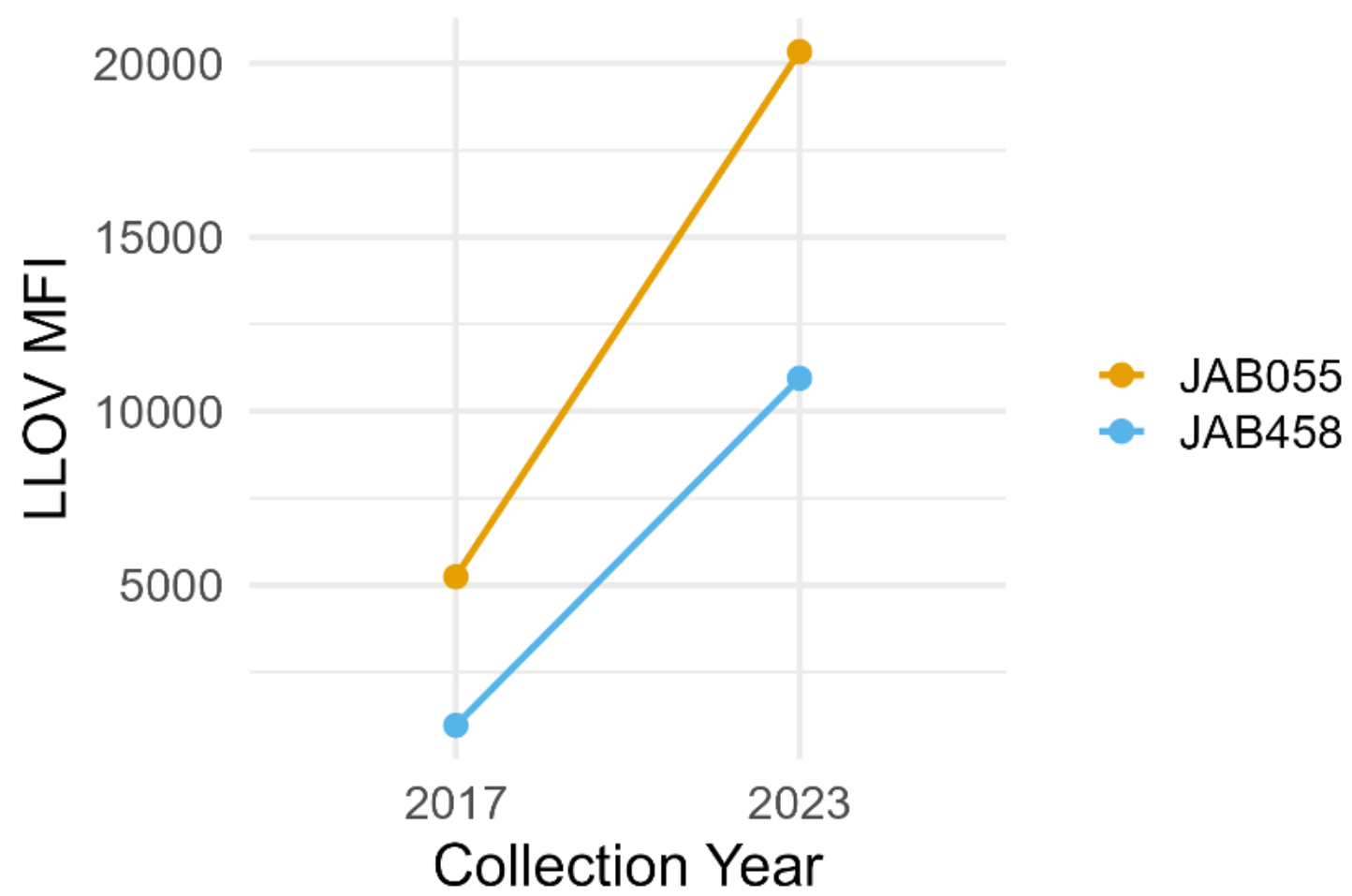

6. Lines 292–296: It seems the authors believe exposure was limited to BDBV-like viruses, but based on the data presented, this reviewer has concerns that exposure to EBOV through contact with bush meat cannot be ruled out. Since the GP proteins of filoviruses are not highly similar, it is unlikely that cross-reactive antibodies would be easily produced, and exposure to multiple viruses may have occurred through contact with bush meat or other sources.

We appreciate the reviewer's concerns regarding the difficulty of disentangling cross-reactive antibody responses from the antibody responses resulting from multiple infections.

Regarding the comment that "given the GP proteins of filoviruses are not highly similar, it is unlikely that cross-reactive antibodies would be easily produced", we refer to evidence in the literature

which suggests that exposure to orthoebolaviruses produces cross-reactive antibodies against the glycoprotein of other related orthoebolaviruses (2). In order to examine cross-reactivity of human serologic responses to EBOV, MacNeil et al. developed antigen preparations for all five EBOV species, and compared serologic responses by IgM capture and IgG enzyme-linked immunosorbent assay (ELISA) in groups of convalescent diagnostic sera from outbreaks in Kikwit, Democratic Republic of Congo (n = 24), Gulu, Uganda (n = 20), Bundibugyo, Uganda (n = 33), and the Philippines (n = 18). They observed strong cross-reactive IgG antibody responses to heterologous antigens among all sets of samples.

Additionally, in a previous publication we assessed the performance of the envelope glycoprotein-based IgG MMIA used in this study in a cohort of PCR-confirmed EVD survivors from the Republic of Guinea, and observed significant pan-orthoebolavirus cross-reactivity resulting from EBOV infection (1). Of the 190 EBOV GP-positive EVD survivors: 190/190 (100%) were co-positive to BDBV,188 (98.9%) were co-positive to TAFV GP, 159 (83.7%) co-positive to BOMV GP, 73 (38.4%) co-positive to SUDV GP, and 3 (1.6%) co-positive to RESTV GP (1).

We aim to use *K*-means unsupervised clustering to identify groups of serological responses which are BDBV-like (highest responses against BDBV, and cross-reactivity against EBOV only), and separate them from individuals who have responses which are EBOV-like (highest against EBOV, with cross-reactive responses against other related filoviruses decreasing with phylogenetic distance). Having down-selected these individuals, we then use parallel neutralisation to identify those who have stronger BDBV neutralisation than EBOV neutralisation. If the serological signals seen were the result of cross-reactive antibodies following EBOV exposure to wildlife meat, then we hypothesise that we would see a (i) binding antibody responses similar to those of PCR-confirmed EBOV survivors (ii) neutralisation of EBOV more strongly than BDBV. We agree with the reviewer that we cannot rule out multiple filovirus infections, and have included wording to this effect throughout the revised manuscript, for example line 305:

"Interpretation of serological profiles with binding to multiple antigens is inherently challenging, as such patterns may arise from heterotypic cross-reactivity, sequential or multiple infections, or exposure to antigenically intermediate (potentially novel) filoviruses."

Minor points:
1. "Error! Reference source not found" appears throughout the manuscript (lines 138, 148, 173, 175, 207).
2. In Table 1, the Guinea 2017 cohort has a total number of participants of 511, divided into 228 males and 223 females. However, there are 60 remaining participants not recorded. Could you explain the missing data? Also, it seems that Table 1 contains calculation errors (228/511 = 45%, not 56% for males). In the "Wildlife hunting" section, for 2017 cohort there are 278 hunters and 232 non-hunters, which adds up to 510. There is 1 missing from the total of 511 participants. Similarly, for the 2023 cohort, there is 1 missing participant.
3. On page 4, line 75: It is stated that the wildlife hunters (all male) constituted 54% of the study population, with the remaining 46% reporting close household contact with a hunter. However, Table 1 states that the wildlife hunters constituted 55%. The data must be presented accurately and consistently.

We thank the reviewer for identifying these errors. They arose from a typographical error in the originally submitted table (misreporting 288 males (56%) as 228 (45%)), and from missing hunting-status data for a single participant.

Table 1 has now been corrected so that all totals and percentages are accurate and internally consistent. Hunting-status data are missing for only one participant in each of the 2017 and 2023 cohorts, which is now explicitly indicated in the table and footnotes. The discrepancy between the proportion of wildlife hunters reported in the text and Table 1 has been resolved by recalculating percentages using consistent denominators and updating the manuscript accordingly.

4. In Figure 4, the EBOV survivor pool shows relatively modest neutralization titers. Since they are confirmed survivors, the authors should comment on this.

We agree that neutralisation titres in the EBOV survivor pool are modest; individuals included in this pool were not prescreened for high binding or neutralising antibody responses, and neutralisation levels are known to vary widely across cohorts and assays.

5. The authors should specify which figure supports the content of the text. For example, lines 161–180 and 175–180 lack figure references.
6. Figure 2: The colors are similar (e.g., BDBV and TAFV), making it difficult to distinguish between them. The coloring should be adjusted.
7. The text in Figure 3 is too small, making it hard to grasp both the figure's content and the related paragraphs (lines 132–154). This figure needs to be adjusted.
8. Inconsistent terminology ("nonhuman primates" vs "non-human primates"), grammatical errors ("sera was incubated" should be "sera were incubated") (page 23, line 518), and incomplete supplementary references need to be corrected (page 14, line 232). "[IQR: 4 – 15)" should be replaced with "[IQR: 4 – 15]" (page 5, line 97).

We have corrected and cross-checked all figure citations in the Results to ensure appropriate referencing throughout the text. Figure formatting issues have been addressed to improve clarity, and typographical, grammatical, and terminology inconsistencies, as well as incomplete references, have been corrected across the manuscript and Supplementary Information.

9. On page 22, lines 461–474 "Pseudotyped virus neutralisation assay (PVNA)": Please specify the luciferase substrate used and its incubation time before reading. For NT50 Calculation Validity, the statement "Hill-Slope constraint of less than 0" is biologically implausible for a neutralisation curve (the slope should be positive). This likely reflects a typo or misinterpretation of software settings. Please clarify or correct this. Please use the temperature units consistently (e.g., "37°C" instead of "37 degrees"). Please spell out "TCID50" at the first mention. Please clarify or spell out "DMEM 1%FBS/1%P/1%S" as "DMEM supplemented with 1% fetal bovine serum and 1% penicillin-streptomycin".
10. On pages 23–24, lines 515–529 "Multiplex microsphere-based immunoassay": For antigen-coupled microspheres, please specify which filovirus envelope proteins (e.g., Ebola GP, Marburg GP) were used as antigens. Omitting this information limits reproducibility and interpretation of cross-reactivity. Please define "room temperature" numerically (e.g., 22–25°C) to ensure consistency across experiments.

We thank the reviewer for these detailed comments and have revised the Methods to clarify all PVNA experimental details, including the luciferase substrate and incubation time, corrected the NT50 curve-fitting description, standardised temperature units, expanded abbreviations, and defined $TCID_{50}$ at first mention. The Hill-Slope constraint of less than 0 is in line with the methods

publication “Pseudotype Neutralization Assays: From Laboratory Bench to Data Analysis” (now cited in Methods)(4). The multiplex microsphere-based immunoassay (MMIA) Methods now explicitly list the filovirus glycoprotein antigens used and define room temperature numerically (22–25 °C). The filovirus MMIA antigens (with Genbank Accession no.) are listed in Table 3 of the Supplementary.

Reviewer #2 (Remarks to the Author):

General Comments

This study investigates serological evidence of filovirus exposure in a wildlife hunter cohort in Guinea. I appreciate that the authors included two negative control cohorts (Negative cohort 2015 and 2017, Coyah and Guéckédou; Blood donor cohort, 2020, UK), which strengthen the study design. The detailed questionnaire on wildlife hunting practices adds valuable context, clearly demonstrating that most respondents not only consumed bushmeat but also handled, washed, or prepared carcasses, with documented hunting of bats.

Previous work has shown that orthoebolaviruses are highly cross-reactive and often indistinguishable serologically, particularly EBOV–BDBV–TAFV and SUDV–RESTV. Cross-reactive binding antibody responses were demonstrated in three of the fifteen identified clusters; however, the remaining clusters consisted of individuals with binding antibody responses against only a single filovirus, which is unexpected given the known cross-reactivity. The BDBV cluster presented in Figure 2 is a notable finding, as five of the thirteen individuals in this cluster were able to neutralize BDBV glycoprotein pseudovirus and showed persistent responses in the 2023 samples. However, these filovirus pseudovirus neutralization assays have not been validated against live filovirus neutralization assays, and it is well known that pseudovirus assays can overestimate virus neutralization.

We thank Reviewer #2 for their careful review and constructive suggestions, which substantially strengthened the clarity of the manuscript.

While we agree with the reviewer that authentic virus neutralisation assay remains the gold standard for assessing the functionality of antibodies in blocking viral entry, it necessitates the use of live authentic virus and thus Biosafety Level-4 (BSL-4) containment. As we did not have access to BSL-4 facilities, we instead used pseudovirus, which are widely used in the literature as a low-containment alternative for assessing functional neutralising activity. Published studies have assessed the correlation between pseudovirus and live authentic virus neutralisation assays in a cohort of PCR-confirmed EVD survivors (5).

While we agree with reviewer comments that PVNAs have a lower limit of detection, in this particular case, because we are comparing samples in parallel, we do not consider this to be an issue. However, we will reword to emphasise this in our interpretations, and we thank the reviewer for calling this to our attention.

We have reworded the manuscript more cautiously (line 405): While authentic virus neutralisation remains the gold standard for measuring the presence and magnitude of neutralising antibodies, the biosafety handling requirements of the viruses in question meant that pseudotyped viruses were used as a surrogate, <u>though they may imperfectly represent authentic virus.</u>

The authors should consider analyzing individuals separately and presenting individual results. For specific individuals, it would be helpful to see both binding antibody and pseudovirus neutralization

responses across the panel of filoviruses to better assess true seropositivity and patterns of cross-reactivity.

We agree with the reviewer that presenting full binding and neutralisation profiles across all filoviruses at the individual level would be informative, and have added this as a limitation in the discussion (line 406). However, the available serum volumes were limited and did not permit comprehensive neutralisation testing of all individuals across the full filovirus panel. We have therefore focused pseudotyped virus testing on selected samples and viruses that were highlighted by the K-means clustering down-selection process for further testing.

Finally, I suggest that the manuscript's discussion of individual filoviruses and the attempts to link exposure to specific filoviruses with particular land-use changes may be somewhat premature, especially given the acknowledged cross-reactivity (lines 365–366). I believe that such detailed conclusions would be better supported if a specific virus were confirmed in the region through PCR and/or virus isolation.

We agree with the reviewer that attribution of exposure to specific filoviruses and detailed linkage to land-use changes should be interpreted cautiously in the absence of virological confirmation, and we have revised the Discussion accordingly. We aimed to take the approach of the wide literature on geostatistical modelling of serological data (different to modelling current infections) to explore relationships between serological indicators at population level and explanatory environmental variables (6).

We now explicitly frame the land-use analyses as associations with serological patterns consistent with antigenically related orthoebolaviruses, rather than definitive evidence of exposure to specific viral species. These observations are presented as hypothesis-generating and intended to identify ecological contexts associated with distinct antibody profiles. In order to better reflect this, language implying species-specific attribution has been softened throughout, including in the abstract.

Specific Comments

Abstract: The list of viruses includes filoviruses, not species of Filoviridae. It should read "filoviruses." Thank you - we have corrected this and standardised terminology throughout the manuscript.

Line 15: Consider adding a cited estimate for deforestation in Guinea to support this statement. A citation has been added.

Line 41: There has only been one reported human case of TAFV infection. Recommend revising the statement to reflect that there is insufficient evidence to claim it causes milder disease. Also, change "asymptomatic human disease" to "asymptomatic infection."

Thank you – these corrections have been made (line 41):

"The severe pathogenicity of many filoviruses has been established (with EBOV, MARV, Sudan (SUDV), and Bundibugyo (BDBV) viruses causing human outbreaks with case fatality rates ranging from 25% to 90%), while others may cause milder (TAFV) or even asymptomatic human infection (RESTV)."

Lines 85–86: Revise to the current nomenclature: "Ebola Zaire" should be referred to as "Ebola

virus."
Thank you - we have corrected this throughout the manuscript.

Lines 146–149: Clarify whether the PCR-confirmed EBOV survivor serum samples were run side-by-side with these specimens, and if so, please include these results in the corresponding figure.

Yes, PCR-confirmed EBOV survivor sera were run side-by-side with study specimens and are shown in the figures labelled "EBOV survivor pool".

Lines 189: Consider revising the subtitle to: "Durable EBOV antibody responses, six years post-2013–16 West African EBOV epidemic." We don't know if these individuals are "immune". Thank you for this comment – we agree and have revised accordingly.

Figure 3: The figure is too difficult to see as presented. I was not able to review. We apologise that Figure 3 was difficult to review in the original submission. The figure has now been revised to improve clarity and readability, including increased font sizes and adjusted layout, and the updated version is provided in the revised manuscript.

Lines 276–277: Emphasize that this is a pseudovirus neutralization assay, not a live virus assay. Revised for clarity.

Line 298: Clarify whether the positive association between primate hunting and anti-BDBV responses was statistically significant in the multivariate analysis.

We thank the reviewer for this comment. We stated in the manuscript (line 328) "primate hunting was positively associated with anti-BDBV antibody responses in the multivariable analysis" but agree it was insufficiently clear in the results. We have now reiterated this more explicitly in the results (line 252): Nonhuman primate consumption was associated with higher odds of BDBV cluster seropositivity in both univariable (OR = 3.49, 95% CI: 1.25–9.74, *p* = 0.017) and multivariable models (aOR = 5.03, 95% CI: 1.70 – 14.90, *p* = 0.004) (Supplementary, **Error! Reference source not found.**) Additionally, we have added a Supplementary Table for clarity (Table 10).

Line 372: Unless there is a direct comparison with live virus neutralization, I feel the filovirus pseudovirus neutralization assays should not be described as a well-established surrogate.

We have therefore revised the manuscript to remove language describing PVNAs as a "well-established surrogate" and to reflect their limitations more explicitly (line 405):

While authentic virus neutralisation remains the gold standard for measuring the presence and magnitude of neutralising antibodies, the biosafety handling requirements of the viruses in question meant that pseudotyped viruses were used as a surrogate, though they may imperfectly represent authentic virus.

Line 461: Specify the titration range of the three-fold dilutions.
1/20, 1/60, 1/180, 1/540, 1/1620, 1/4860, 1/14580. Methods have been revised to include this.

Reviewer #3 (Remarks to the Author):

Major
This manuscript by Hood et al. investigates an interesting and important topic: the potential undetected exposure of individuals to different filoviruses and sheds new light on this topic. The manuscript is well written and having paired follow-up examples is a great advantage compared to other seroprevalence studies. Also, the broad antigen panel is that is used, is interesting. The major downside of the manuscript is that it explores too many different questions (hunting practices, antibody profiles, association with land-cover) and fails to go into depth. The many objectives stand in the way of a cohesive and convincing story. I wonder if a manuscript focused on BDBV seroconversion, with some additional triangulation that corroborates that this was really exposure to BDBV would not be the better option.

We thank Reviewer #3 for their thoughtful and detailed comments, and recognition of what we also find to be an important topic.

We appreciate the reviewer's concern that the manuscript addresses multiple components, which may affect narrative focus. Our intention in integrating serological data with hunting practices and land-cover analyses was to provide contextual information that supports interpretation of the antibody findings and to explore potential ecological and behavioural correlates of exposure within a One Health framework. Further, analysing these multiplex serological data enables evaluating common pathways or patterns of exposure to different pathogens.

In response to this comment and others, we have revised the manuscript to improve the focus, including moderating species-specific interpretations and more clearly framing the land-use and behavioural analyses as hypothesis-generating and supportive rather than definitive. We believe this interdisciplinary approach remains appropriate for Nature Communications and hope it will be of interest to its broad readership, while the revisions strengthen the clarity and balance of the overall narrative.

- My main comment is that I'm not yet convinced of the main conclusion that the signals picked up by serology represent an exposure to the filovirus in question. The control population, which is used to determine the cut-offs of the assay are UK residents. However, in my opinion, this is not a good reference population. The issue with serology in malaria-endemic settings is that repeated malaria exposure causes a polyclonal gamma expansion, leading to aspecific binding of antibodies and a higher rate of false-positive results on serology. The specificity of serological assays, therefore, changes in these settings. I'm not sure if the neutralizing antibody assay used here do not also exhibit this potential aspecific binding to some degree.

Could any of the following things be done to exclude this issue: use a (larger) cohort of controls from malaria endemic areas as controls rather than the UK population, check if aspecific binding to other pathogens that are not endemic also occurs, check for malaria antibodies or total IgG and see if it correlates with filovirus antibodies… I understand that it is not possible to do all of this, but some additional steps should be taken to ensure that this is not a specific binding. Supplementary figure 10 is revealing in this regard. The distribution of antibody profiles completely overlap here between hunters and non-hunters.

We agree with the reviewer that calculating a threshold based on population measures (mean and standard deviation) of the antibody distribution in the presumed seronegative cohort would be

unsuitable due to the differing immunological background between the Guinean and the UK blood donor populations. Instead, we utilised unsupervised classification methods to identify groups from the full distributions of observed responses. While finite mixture models and similar methods are widely used to model two or more distributions from single antigen responses, we feel it is more appropriate to assess exposures to multiple related pathogens.

For these reasons, an unsupervised machine-learning approach was utilized to partition the data into qualitatively different immunological or "exposure" profiles rather than using a binary endpoint to translate a continuous MFI. K-means clustering has the additional advantage of fitting groups within the same population, so that we are not reliant on UK negative controls, which may have very different immunological backgrounds.

To the reviewer's point regarding aspecific binding: we present unpublished data to demonstrate responses in a non-wildlife hunting cohort of controls from urban Guinea (also a malaria endemic area) to demonstrate that they do not appear to show aspecific binding patterns against filovirus antibodies (below).

Finally, we agree that the overlap between the hunters and non-hunters was misleading, as we failed to appropriately highlight that majority of "non-hunters" were the close contacts of the hunters, and thus exposed to wildlife carcasses through butchering and consumption (therefore had similar exposures). Based on this, and other constructive reviewer comments, we have significantly revised our analysis and discussion of hunters vs. "non-hunters" and removed related figures.

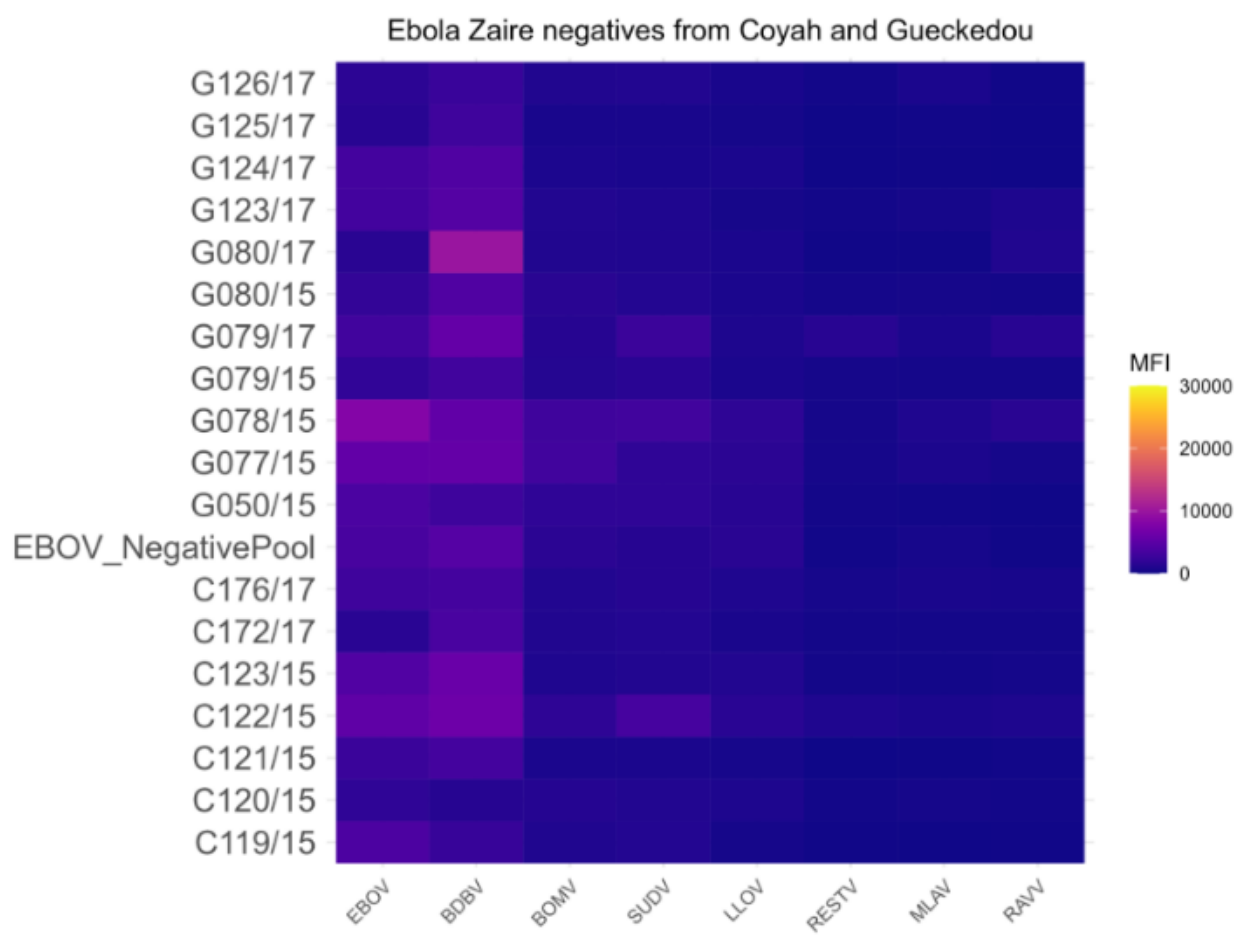

- An argument against a specific binding is the fact that the antibody profiles in Figure 1 may exhibit a bimodal distribution, with the second peak corresponding to real exposure. I think Figure 1 can therefore benefit from adding the population of EVD-confirmed individuals to see how the distribution of antibodies from infected individuals shapes and compares to the other populations. In addition, I think the manuscript could benefit from a statistical analysis which explores in how far these population differ from each other

We thank for reviewer for their constructive comments, and as suggested, an additional figure has been included in the manuscript (Supplementary, Figure 10).

- Line 55: the authors write: 'ongoing seroconversion'. However, there is no data in the manuscript on those participants who were seronegative at the beginning of the study, but seropositive at the

end. They only provide information on those with decline antibodies. Nevertheless, a more in-depth characterization of those that seroconvert looks to me most interesting. What is their profile, do they cluster geographically, did they recall any illness?

A more specific discussion of the 2 individuals who seroconverted to LLOV/LLOV-related virus between 2017 and 2023 was made, and an additional figure was included in the Supplementary (Figure 11). Unfortunately, due to the small numbers risk factors (including geography) could not be statistically investigated, and the questionnaire at the time of sample collection did not ask about recalling previous illness.

Line 73: Can the others give details on how many individuals were approached, but refused participation, and add numbers on the size of the villages to estimate how representative the study population is.

We agree that information on village-level sampling is important for interpreting heterogeneity. While accurate population sizes for individual villages are not available, we have now included a table reporting the number of individuals approached and the number sampled in each village, which provides transparency regarding sampling intensity and representation across sites. This has been added to the Supplementary Information as Table 9.

Line 94 onwards: I would avoid the term bushmeat as is done elsewhere in the manuscript, as it has a stigmatizing connotation.

Thank you for your comment. We have attempted to avoid stigmatising language throughout and are grateful for your careful review.

- Line 94 onwards: in this paragraph, only parts of the result are described in a narrative paragraph. While the section is well written, the authors should include a table with all the data, including all the questions asked and the proportions of the different answers. Otherwise, it is difficult for the reader to interpret the data correctly.

Thank you for your constructive comments – an additional table (Supplementary, Table 5) has been added to the manuscript, as suggested.

Question	n	N	%
**Do you ever hunt or trap bats?**			
Yes	156	641	24
No	485	641	76
**Do other members of your household hunt or trap bats?**			
Yes	51	491	10
No	440	491	90
**Do you handle bats during preparation or cooking for food?**			
Yes	274	535	51
No	261	648	40
**Do you buy bats from other people or markets?**			
Yes	130	646	20
No	516	648	80
**Which season do you hunt more frequently?**			

Start of dry season	122	133	92
Middle of dry season	4	133	3
End of dry season	5	133	4
Start of rainy season	2	133	2
Middle of rainy season	0	133	0
End of rainy season	0	133	0
**What do you do with the hunted bats?**			
For food only	145	155	94
For food and to sell	8	155	5
Other	1	155	1
To sell only	1	155	1
**Do you ever go into bat caves?**			
Yes	139	546	25
No	407	546	75
**Do you do any other kind of activities with the bats when inside the cave?**			
No	85	86	99
Yes	1	86	1
**On a normal visit, how long (minutes) do you spend inside the cave?**			
Median [IQR]	2 [0 - 20]		
Mean [Min - Max]	13 [0 - 60]		
**Do you harvest bat guano?**			
Yes	2	648	0
No	646	648	100

- Line 138: I'm not convinced by the relevance of the machine learning algorithm. The K-means clustering will provide an output based on the number of predefined clusters. However, the authors do not provide data that validates these clusters, so it is unsure if they have any biological meaning.
- What is the rationale for choosing 15 clusters (k = 15)? Does not

We appreciate the reviewer's concerns regarding the relevance, validation, and biological interpretability of the k-means clustering approach, as well as the rationale for selecting k = 15.

Initial attempts to define seropositivity relied on two commonly used approaches for multiplex immunoassays: (i) thresholds based on presumed seronegative controls (UK blood donors) and (ii) finite mixture modelling. The first approach was unsuitable in this context due to the differing immunological background between the Guinean and the UK blood donor populations. Approaches to analyse exposure data from a single antigen, such as finite mixture models, do not include data from other antigens, which may be cross-reactive. Additionally, this imposes binary classifications on continuous MFI data and are highly susceptible to misclassification in the presence of heterotypic cross-reactivity, as we demonstrate in PCR-confirmed EBOV survivors, where extensive orthoebolavirus co-reactivity is observed (1).

K-means clustering was selected because it is well suited to low-dimensional data with approximately spherical cluster structure and has been widely used to interpret multiplexed immunoassay datasets (7, 8). Importantly, clustering was not used as a final classification of exposure, but as an exploratory tool to group individuals with similar antibody profiles for downstream confirmatory analyses.

The number of clusters (k=15) was determined through multiple complementary validation approaches. First, we visually examined the results of different numbers of clusters and evaluated this using the elbow method. As we aimed to identify groups of exposures across different pathogens, we only include k > 4. The elbow method demonstrated diminishing returns in within-cluster sum of squares beyond k=15 (after K=15 was less than 21323813075). The gap statistic confirmed that k=15 produced clustering structure substantially better than expected by chance, while silhouette analysis showed adequate cluster cohesion (average silhouette width = 0.23). The Calinski-Harabasz index and Davies-Bouldin index metrics indicated a range of statistically acceptable solutions rather than a single optimum.

Visual inspection across k = 2–25 showed that k = 15 provided the best balance between resolution and over-fragmentation, capturing distinct antigenic patterns across nine filovirus glycoproteins while maintaining coherent cluster structure. Given that this analysis encompasses nine filovirus species (EBOV, BDBV, TAFV, BOMV, SUDV, LLOV, RESTV, MLAV, and RAVV), k=15 provides the resolution for identifying species-specific serological profiles for downstream confirmatory testing, without over-fragmenting the data into statistically indistinguishable subgroups.

To address concerns regarding biological meaning, we have added new supplementary figures showing individual-level MFI responses within clusters. These demonstrate that clusters share consistent ordinal antigenic profiles (e.g., EBOV > BDBV > BOMV or BDBV > EBOV), supporting the biological coherence of the clustering solution. We now explicitly frame clustering as a hypothesis-generating, exploratory step used to guide targeted neutralisation testing, rather than as a definitive exposure classification; we have significantly revised the results (line 166), discussion (lines 304 - 312) and methods (line 533-536) in order to highlight this rationale and its limitations.

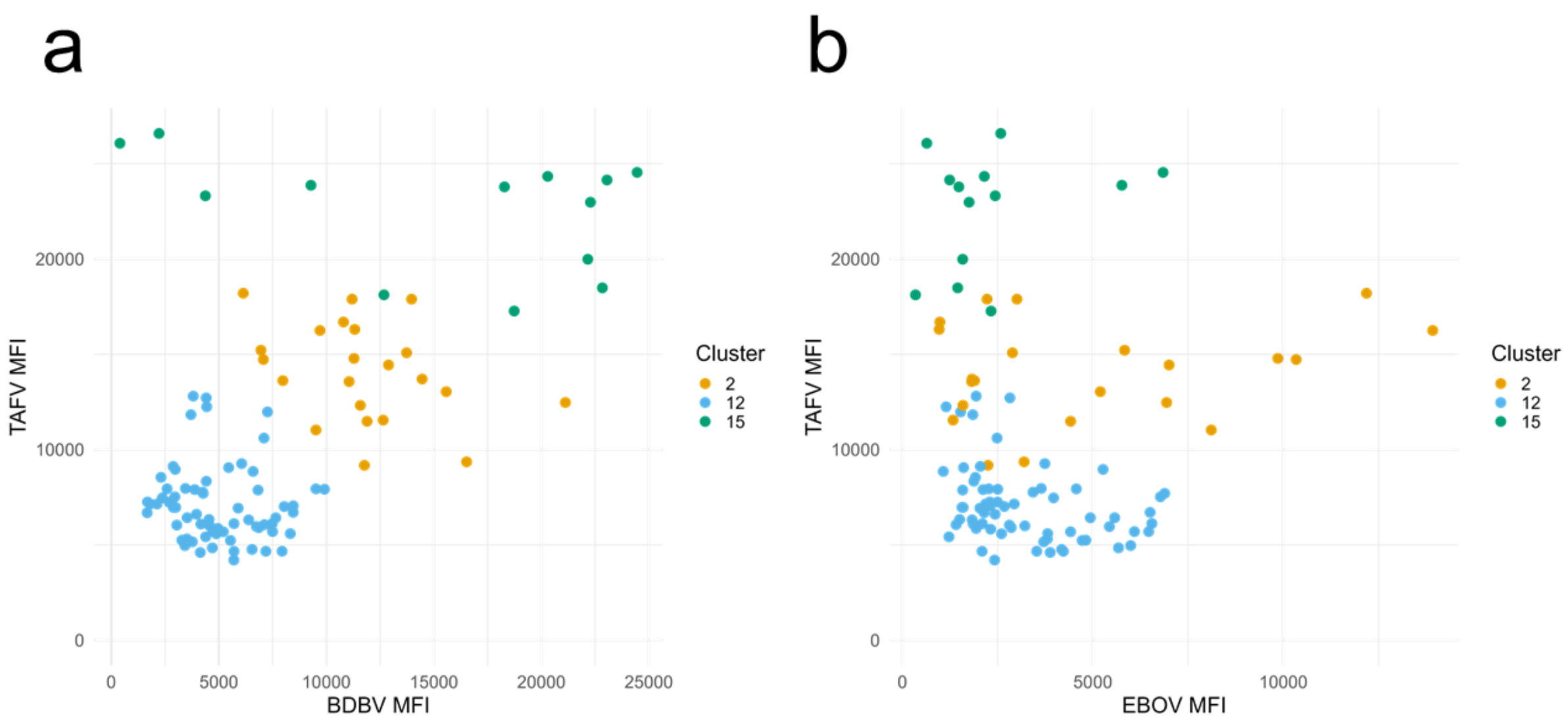

Figure 1 Individual MFI responses against (a) TAFV and BDBV (b) TAFV and EBOV for all hunters from the Guinean wildlife hunting cohort (2017 and 2023) who were categorised into Clusters 2, 12 and 15 from the K-means clustering algorithm analysis for k=15.

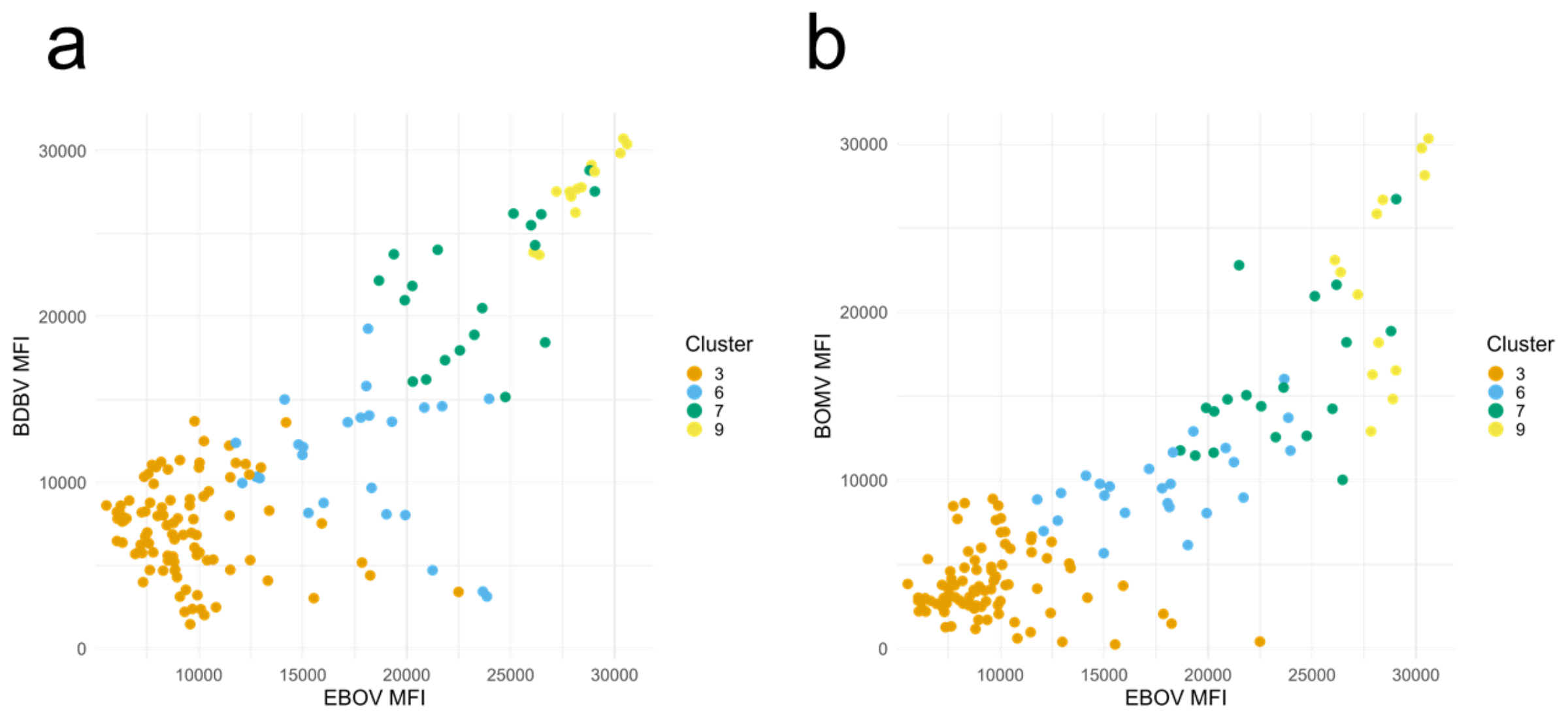

Figure 2 Individual MFI responses against (a) BDBV and EBOV (b) BOMV and EBOV for all hunters from the Guinean wildlife hunting cohort (2017 and 2023) who were categorised into Clusters 3, 6, 7 and 9 from the K-means clustering algorithm analysis for k=15.

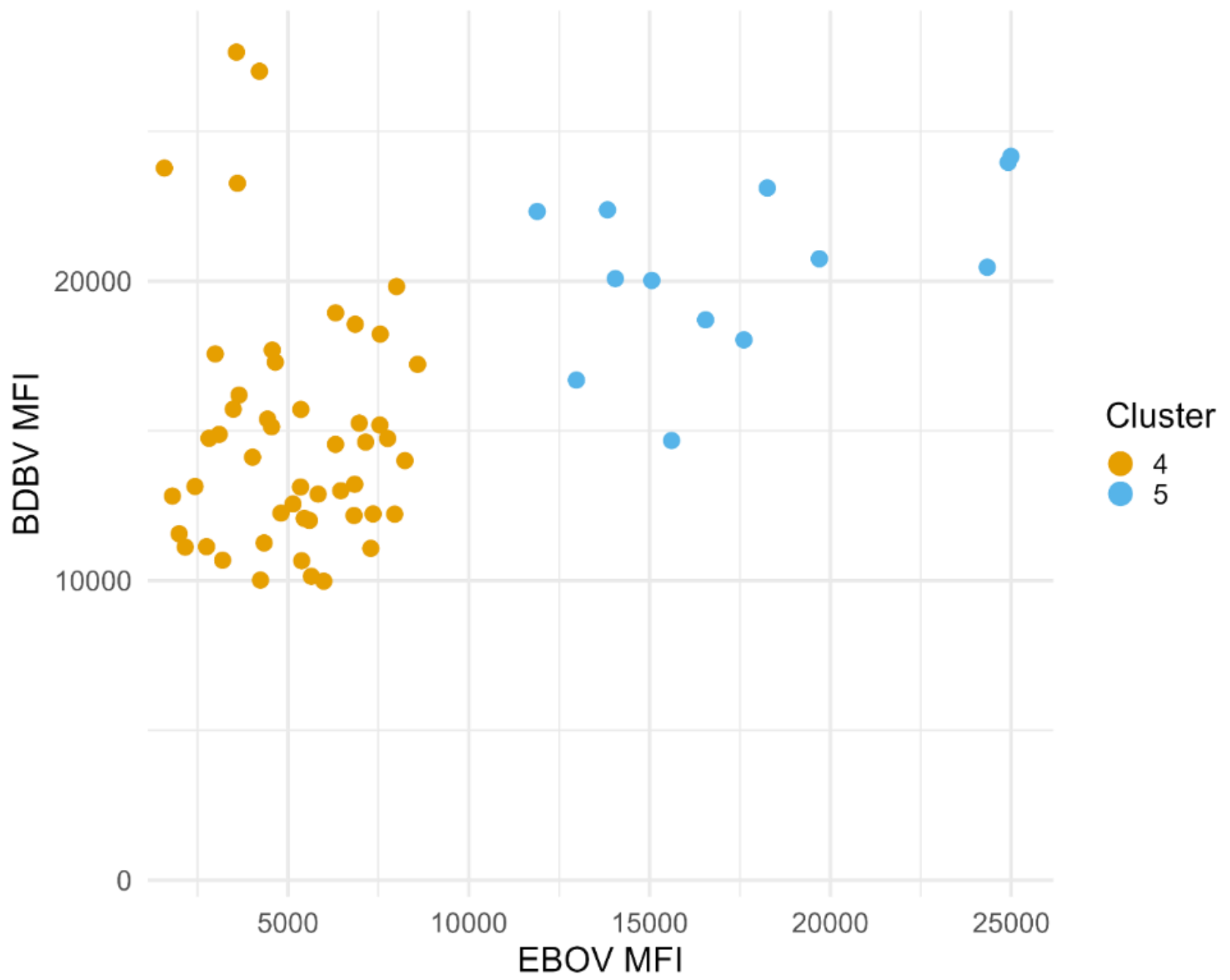

Figure 3 Individual MFI responses against BDBV and EBOV from the Guinean wildlife hunting cohort (2017 and 2023) who were categorised into Clusters 4 and 5 from the K-means clustering algorithm analysis for k=15.

- Line 146: According to the authors based on the antibody profile 57 individuals were 'likely EBOV exposed'. Would this not provide an opportunity to biologically validate at least these 3 clusters? Is there data in the questionnaire that indicate that have ever been in contact with an EVD patient, or were from villages affected by EVD. If they have antibodies, they were not just exposed, but infected. This would mean that all these individuals had an asymptomatic EVD infection, which seems unlikely.

The high seroprevalence of anti-EBOV GP antibodies that we observed in the Guinean study population (6.9% (78/1129) based on a conservative (5 standard deviation) cut-off derived from the UK blood donor population) is comparable to estimates from nearby EVD-affected regions. For example, EBOV GP seropositivity was reported at 3.57% (95% CI: 1.55–6.92%, 8/224) in the general adult population of Meliandou village in Forested Guinea (9), 7.2% (63/878) in western Guinea (10), or 8.4% (95% CI: 7.0 – 10.0%, 107/1282) in neighbouring Sierra Leone (11). The consistently high seroprevalence reported across these studies lends credence to, what we agree is the somewhat controversial notion of asymptomatic or paucisymptomatic EBOV infection.

To investigate further, we performed a rapid literature review of orthoebolavirus seroprevalence surveys conducted in West Africa up until August 10th 2025 that have been published in peer-reviewed scientific journals, in English. We identified 24 studies assessing orthoebolavirus seroprevalence in West Africa: 19 in countries affected by the 2013-2016 epidemic (during or shortly after the outbreak) (Table 1). Majority of these (14/24) assessed seroprevalence in high-risk populations: individuals with fevers of unknown origin (FUO), asymptomatic contacts of known EVD cases, or healthcare workers (HCW). Studies which assessed seroprevalence in the general population (10/23) observed EBOV seroprevalence ranges from 1.3 – 19.4% (Table 1). Interestingly, the highest seroprevalence estimates came from Forested Guinea, where 19.4% (52/268) were seropositive at least one EBOV antigen; 1.1% (3/268) were NP reactive and 9.33% (25/268) were reactive to GP-m. Several studies (highlighted in Table 1) also provided evidence of the circulation of EBOV in the area prior to the 2013-2016 outbreak: for example, 8.5% of febrile individuals 2011-2012 (n=129) in Guinea prior to the 2013-2016 West African EBOV outbreak (12).

Specifically considering asymptomatic infection, given the challenges of surveillance in remote locations, many outbreak investigations rely on retrospective detection, therefore we believe they are likely to overlook mild clinical presentations of EVD. Of the studies which have taken cross-sectional seroprevalence of asymptomatic contacts of EVD cases, they report anywhere from 1.0% (2/210) in Uganda(13), 2.5% (10/404) in Yambuku, Democratic Republic of the Congo (DRC) (14); 2.6% (10/388) in Sierra Leone (15), 4.0% (4/101) in Kikwit, DRC (16); 6.0% (115/1909) in Liberia(17); 6.5% (12/185) in Kono, Sierra Leone(18); 21.4% (12/56) in Gabon(19); 45.9% (11/24) in Gabon(20); up to 71% in Gabon (21). A meta-analysis drawn from studies estimated a secondary attack rate of 27% (95% CI, 14.5–39.6) (22).

Based on this available evidence, we believe that the rate of unrecognized EBOV exposure is high in this region, as reflected in our study.

Finally, we are not making the claim that all of these individuals had asymptomatic (as opposed to symptomatic) EVD infection, as we do not have prior medical history for them. Unfortunately, due to the significant stigma and negative associations regarding Ebola research in the area, our local collaborators advised against asking questions regarding Ebola history in order to avoid misunderstanding with study participants. Biological validation of these clusters would therefore involve investigation of antibodies, which we have done.

Table 1 Summary of published orthoebolavirus seroprevalence studies conducted in West Africa, 1978 – 2025

Population sampled	Country	Year	Seroprevalence	Assay	Citation
Very low risk (general population in non-outbreak setting)					
General population	Liberia	1978 – 1979	6% (26/433)	IF	Knobloch et al., 1982, Ann de

					l'Institut Pasteur (23)
General population (including epilepsy patients)	Liberia	1986	13.4% (30/225)	EBOV -May IF	Van der Waals, 1986, Trop and Geo Med (24)
General population	Nigeria	1988	EBOV: 1.3% (22/1677); SUDV: 1.8% (30/1677)	IF	Tomori et al., 1988, Am Jour of Trop Med and Hyg (25)
General population prior to outbreak	Guinea	2012	≥1 EBOV antigen: 10.4% (154/1483); GP-Mayinga: 5.5% (81/1483); NP: 0.6% (9/1483); VP40: 4.2% (62/1483).<br><br>Forested Guinea — ≥1 antigen: 19.4% (52/268); NP: 1.1% (3/268); GP-m: 9.33% (25/268)	NP, GP, VP40 EBOV IgG MMIA	Keita et al., 2018, Am J Trop Med Hyg (26)<br>*
General population in EVD unaffected area	Mali	2015	3.7% GP-positive and 4.0% NP-positive and 1.5% both<br>6.1% (37/600); GP: 3.7% (22/600); NP: 4.0% (24/600); GP and NP: 1.5% (9/600)	GP, NP IgG ELISA	Bane et al., 2021, EID (27)
General population in EVD unaffected area	Guinea	2022	GP-Kiss: 6.6% (58/878); GP-May: 7.2% (63/878); VP40: 3.6% (32/878); NP: 0.8% (7/878)	NP, GP-Kiss, GP-May, VP40 EBOV; GP, VP40 SUDV; GP, VP40 BDBV, GP RESTV, GP BOMV IgG MMIA	Hounmenou et al, 2024, Int Journ of ID(10)
General population (rural population and case-control study examining nonhuman primate bites)	Cameroon	2024	Rural population: ≥2 EBOV antigen: 10.2% (81/795); ≥2 SUDV antigen: 9.6% (76/795); BDBV antigen: 3.5% (28/795)<br>NHP bites: ≥2 EBOV antigen: 15.6% (97/622); ≥2 SUDV antigen: 14.1% (88/622); BDBV antigen: 6.1% (38/622)	GP, NP, VP40 EBOV; SUDV, BDBV, BOMV, RESTV IgG MMIA	Ramassamy et al. 2024, Journ of ID (28).
Low risk (general population in outbreak setting)					
General population in EVD affected community	Sierra Leone	2015 – 2016	7.5% (12/187)	GP and NP IgG ELISA	Richardson, 2016, PLoS Neglected Tropical Diseases(18)
General population in EVD affected community	Guinea	2017	3.6%, 95% CI: 1.55–6.92 (8/224)	GP IgG ELISA (oral fluid)	Timothy et al., 2019, Lancet ID (9)
General population in EVD	Sierra Leone	2016 – 2018	8% (107/1282)	GP IgG ELISA (FANG)	Manno et al., 2022, EID (11)

affected community					
High risk (contacts of confirmed cases, febrile patients (Fevers of Unknown Origin), healthcare workers)					
Febrile patients, General population	Cameroon, Congo, DRC, Ghana, Uganda	1997–2012	2-3.5%	PV MNA then confirmed by NP IgG ELISA	Steffen et al., 2019, EID (29)
Febrile patients	Sierra Leone	2006 – 2008	8.6% (19/220)	EBOV cell lysate IgM/IgG ELISA	Schoepp et al., 2014, EID (30)
Acutely ill patients	Mali	2009 – 2013	0% (0/376)	EBOV cell lysate IgM/IgG ELISA	Safronetz et al., 2016, EID (31)
Febrile patients	Guinea	2011 – 2012	8.5% (11/129)	NP IgG MMIA	Surtees et al., 2020, PLoS Neglected Tropical Disease (12)
Febrile patients	Sierra Leone	2011 – 2014	22% (53/242)	GP, VP40 IgG/IgM ELISA	Boisen et al., 2015, Viral Immunology (32) *
Febrile patients	Guinea	2016 – 2017	0%	NP IgM ELISA	Dedkov et al, 2021, Int J of Env Res and Public Health(33)
Asymptomatic or paucisymptomatic contacts of EVD survivors	Sierra Leone	2014 – 2015	Paucisymptomatic: 12.0%, 95% CI: 6.1–20.4% (11/92); Asymptomatic: 2.6%, 95% CI: 1.2–4.7% (10/388)	GP IgG ELISA (oral fluid)	Glynn et al., 2017, Lancet ID(15)
Asymptomatic or paucisymptomatic contacts of EVD survivors	Sierra Leone	2015 – 2016	Paucisymptomatic: 25%, 95% CI: 14–40%; Asymptomatic: 4%, 95% CI: 1–14%	GP IgG ELISA	Kelly, 2018, Journal of ID(34)
International HCW returning from West Africa	West Africa	2015 – 2016	0.0% (0/268)	GP IgG ELISA (oral fluid)	Houlihan et al., 2017, PLoS Medicine (35)
Asymptomatic or paucisymptomatic contacts of EVD survivors	Liberia	2015 – 2017	8.25% (222/2688)	GP IgG ELISA (FANG)	Kelly et al., 2022, Lancet ID (36)
Asymptomatic or paucisymptomatic contacts of EVD survivors	Liberia	2015 – 2017	11.6% (222/1909)	GP IgG ELISA (FANG)	Gayedyu-Dennis, 2023, J Infect Dis (17)
Asymptomatic contacts of EVD survivors	Guinea	2016 – 2017	Paucisymptomatic: 8.33%, 95% CI: 5.01–12.80% (18/216); Asymptomatic: 3.32%, 95% CI: 1.23–5.01% (39/1174)	GP, NP, VP40 IgG MMIA	Diallo et al., 2019, Lanced ID (37)

Asymptomatic or paucisymptomatic contacts of EVD survivors	Liberia	2016 – 2017	Paucisymptomatic: 5.2% (22/421); Asymptomatic: 4.3% (18/421)	GP IgG ELISA (FANG)	Kelly et al., 2022, Open Forum Infectious Diseases(38)
Asymptomatic contacts of EVD survivors	Sierra Leone	2017	12.7% (34/267)	GP IgG ELISA	Halfmann et al., 2019, PLoS Neglected Tropical Diseases (39)

IF = Indirect immunofluorescence, ELISA = Enzyme-linked immunosorbent assay, IgG = Immunoglobulin G, IgM = Immunoglobulin M, GP = Glycoprotein, NP = Nucleoprotein, VP40 = 40-kDa viral protein , EBOV = Ebola virus, May = Mayinga, Kiss = Kissidougou, SUDV = Sudan virus, BDBV = Bundibugyo virus, RESTV = Reston virus, BOMV = Bombali virus, MMIA = Multiplex microsphere-based immunoassay, PV = Pseudovirus, MNA = Microneutralization assay, FANG = Filovirus Animal Nonclinical Group, HCW = Healthcare worker, EVD = Ebola virus disease.

*Evidence of EBOV circulating in West Africa prior to outbreak

- Line 162: ‘antibody profiles consistent with exposure to …’: I find this wording a bit misleading, as we don’t have validated antibody profiles of individuals with a confirmed infection with these viruses.

Thank you for your comment – below we show on the antibody profiles of PCR-confirmed EVD survivors from Guinea as additional support (1).

Anti-filovirus responses in EBOV survivors, Coyah and Guéckédou

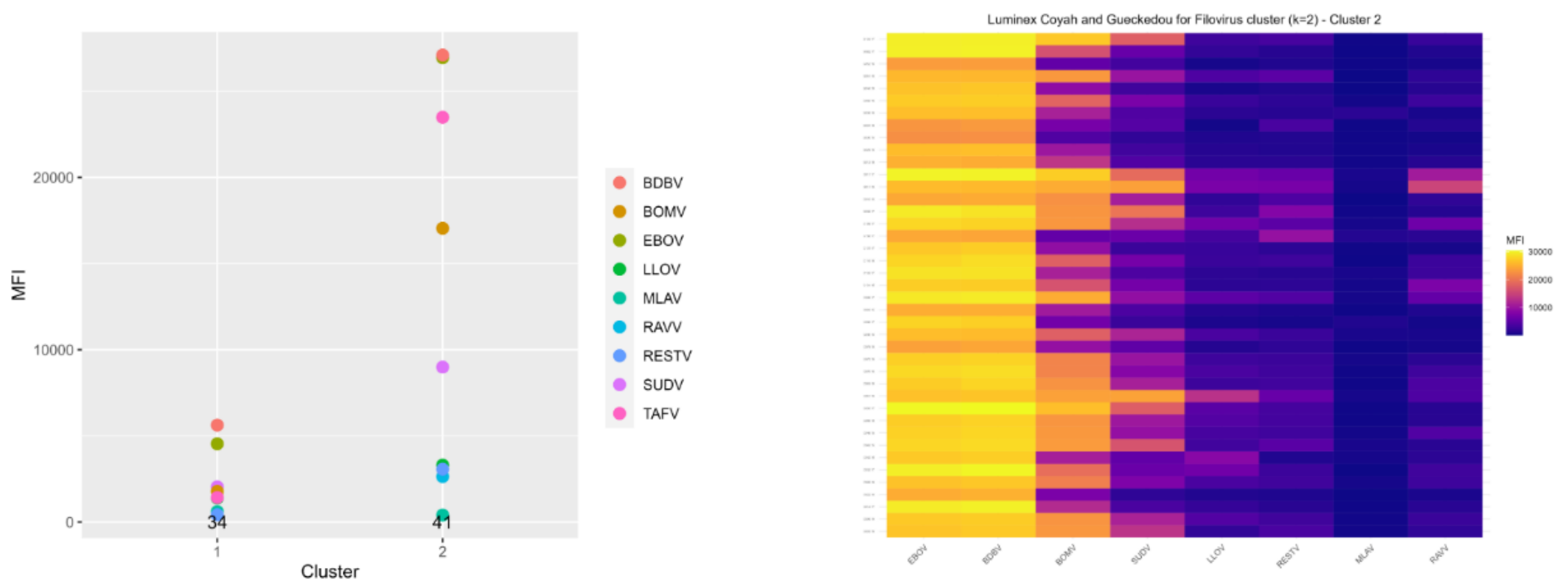

- Line 168: the authors write: ‘5 individuals neutralized BDBV more strongly’ However, the 95% CI’s in figure 4 completely overlap. This statement therefor does not seem to be correct. Are the 95% CI calculated based on sample replicates? Is there not a way of comparing the antibody mean of the log antibody titers between the different clustering groups. Similarly for the paragraph starting from line 174.

We thank the reviewer for this important statistical point. We agree that asserting “X neutralised Y more strongly” is inappropriate where 95% confidence intervals overlap, so we have reworded the Results and Figure 4 legend throughout to explicitly note when confidence intervals overlap and

avoid categorical claims of a statistically significant difference in neutralisation (line 180, line 187, line 332). Additionally, we also clarified the NT50 methods: NT50 and 95% CIs were derived from nonlinear regression of serial-dilution replicates for each sample (line 516). As we did not perform neutralisations for all viruses for each individual in each cluster (due to limited sample volume), we were not able to appropriately compare the antibody means between the clustering groups.

- Line 168: five out of how many? Clarified.

- Line 171: Does this data contradict the fact that these antibody profiles correspond to exposure to BDBV?

We do not interpret the observed data as contradicting this interpretation, as the antibody profiles demonstrate binding reactivity to both BDBV and EBOV, and further, not all binding antibodies are neutralising (hence the ability of neutralisation tests to have better specificity than binding antibody assays).

- Line 180: What is the rationale of not showing these data. Are other clusters also evaluated using neutralizing assays? Again, a more systematic validation of these clusters is needed.

Neutralisation assays were performed on selected individuals from clusters exhibiting the highest binding antibody responses, using parallel testing within clusters where sufficient serum volume was available. It was not feasible to perform neutralisation assays for every individual or across all clusters due to limitations in serum availability, assay throughput, and the availability of pseudotyped viruses (e.g., MLAV pseudotypes could not be generated). We have now clarified this rationale and its limitations (line 406) more explicitly in the Methods and Discussion and frame the neutralisation data as targeted, supportive validation rather than comprehensive cluster-wide testing.

- Line 206: In this paragraph, the authors explore differences between villages. However, in order to interpret this data it is necessary to know the number of participants per village. There is substantial heterogeneity; however, the overall number of participants is low compared to the number of villages. Already, having only 10-20 participants per village, leads to random effects between villages. However, maybe for some villages only a few individuals were recruited. This information is needed.

These surveys were not designed to be representative, but rather purposively sample wildlife hunters and their families. While accurate population sizes for individual villages are not available, we have now included a table reporting the number of individuals approached and the number sampled in each village. This has been added to the Supplementary Information (Table 9).

- Line 208: According to Figure 6, villages are anonymized, which I think is a good idea. However, in line 208, the so-called hotspot name is disclosed. I would avoid this.

Thank you for your careful review; we have removed the village name from the manuscript.

- In general, were participants asked if they were ever diagnosed with EVD?

Due to persistent social stigma and sensitivities surrounding Ebola research in the region, local collaborators advised against collecting self-reported histories of Ebola infection to minimise the risk of misunderstanding or distress among participants.

- Fig 7: Heatmaps are often difficult to interpret, which is especially the case here. I wonder if a graph would be more suitable. It is difficult to interpret the color changes of time.

We appreciate the reviewer's comment regarding the presentation of Figure 7. We agree that heatmaps can be challenging to interpret in some contexts; however, in this case we retained the heatmap format because it allows simultaneous visualisation of longitudinal changes across multiple individuals and antigens, which would be difficult to convey clearly in a single alternative graph. To improve interpretability, we have ensured colouring is colour-blind safe.

- Line 222: the authors describe multivariate models, but provide no information on which parameters were included in the models. They only describe two significant results.

Thank you for your comment – an additional table including the results from the univariable and multivariable logistic regression analysis (with all parameters included in the model) has been added to the Supplementary (Table 10).

- Line 231: Did these populations differ? You state so in the discussion, but I mainly see overlap. Are there any statistical test done?

We agree with the reviewer that differences between hunters and non-hunters were difficult to interpret and largely overlapping, particularly given the high degree of reported wildlife contact in both populations. We realise that we failed to appropriately describe how even "non-hunters" have significant exposure to wildlife meat through butchering and cooking of carcasses. In response to this and other reviewer feedback, we have removed the hunter versus non-hunter comparison from the manuscript to avoid overinterpretation and to improve clarity and focus.

- Line 239: The authors here test the association of 3 different parameters (coverage, shape index, perimeter area ratio), and this for an unknown number of land cover types (three, forest, built area?). They test the association with 5 different antigens. It is unclear from the manuscript how many associations were tested, as the authors only display the significant results. Moreover, from the table it appears to be done on different scale. This would lead to serious of multiple testing. The authors should state which variables exactly were tested, why and also display negative results.

We used a previously, published data-driven approach to explore the proportion of land cover and habitat fragmentation (as measured by shape index and perimeter area ratio) across multiple spatial scales (500 m, 1 km, 2 km, 5 km and 10 km buffer sizes). A predefined set of landscape metrics (proportion coverage, shape index, fractal dimension index, perimeter–area ratio) were evaluated across major land cover classes (trees, built area, crops), as obtained from ESRI land cover classification derived from Sentinel-2 remote-sensing images captured at a 100-meter resolution in 2017 and 2023. As we were interested in groups with common exposures, we tested for association with predefined serological patterns rather than individual viral species (40). Tested variables and associations, including non-significant results, are all included in the code and dataset which has been made available with the manuscript, and the text has been revised to emphasise that these

analyses are hypothesis-generating, with the methods section expanded to explain this rationale (lines 599 – 612).

- Line 288; in the discussion the authors give 'Individual-level' epidemiological data to further support their hypothesis. However, they should do so in the results.

In the discussion we make reference to individual-level data, which is presented in the results: "Two of these individuals were household contacts (JAB135, JAB136), also from an EBOV-unaffected village". We have included an additional table for further clarity (Supplementary, Table 7).

Minor:
- The introduction could benefit from a brief recap of filovirus taxonomy
- Line 6: I would use a more nuanced word than hotspot
- Line 15-18 This is a complicated sentence which is difficult to interprate
- Line 22: What do the authors mean with 'more proximal' drivers?
- Line 55: This part seems more fit for a discussion
- Line 71: Who collected the data
- Figure 4: As data are probably pseudonimized, it's better not to disclose study numbers in the legend of the figure. It makes the interpretation of this figure also more complex
- There is an issue with referencing to the figures: all throughout the text it indicates Error! Reference source not found): which complicates the peer review

We thank the reviewer for their constructive comments, which we have sought to address in the revisions, which we believe have substantially improved the manuscript.

Reviewer #4 (Remarks to the Author):

This manuscript reports a serological survey of filovirus exposure in rural hunter communities in forested Guinea, with sampling conducted in 2017 (previously published?) and 2023. The study employs a multiplex microsphere-based immunoassay (MMIA) to detect IgG responses to nine filovirus glycoproteins, along with pseudo-typed virus neutralization assays for functional assessment. The authors also integrate environmental and behavioural risk factor analysis, including land use and hunting activities. The central finding is the detection of binding antibodies against multiple filovirus species beyond Ebola virus (EBOV), including BDBV, TAFV, BOMV, LLOV, MLAV, and RESTV, interpreted as evidence for ongoing zoonotic spillover.
This work addresses an important public health question regarding filovirus ecology. However, interpretation of the serological data is limited by well-known issues of broad cross-reactivity among filoviruses, particularly among Ortho-ebolaviruses. Additional clarity and a more cautious interpretation are warranted, especially regarding the clustering approach and risk factor analysis.

Major comments

Previously published data (2017 cohort) ?
It appears from the Introduction and references (see line 50 [adjust as needed]) that the 2017 wildlife hunter cohort included in this study is substantially or entirely the same as that studied and published previously by Akoi Boré et al., Nature Communications, 2024.
Reference : Akoi Boré, J., et al., Serological evidence of zoonotic filovirus exposure among bushmeat hunters in Guinea. Nature Communications, 2024) (499 bushmeat hunters in Macenta Prefecture collected in 2017).
However, this overlap is not communicated with sufficient clarity or transparency in the current manuscript. While the Introduction states, "[45]. In this study, we return to Forested Guinea to resample and expand the wildlife hunter cohort 6 years later...", the Methods only reference the previous article in the context of blood processing ("Blood processing was performed as described in Akoi Boré et al., 2024 [45]."), rather than explicitly stating the source, characteristics, and degree of overlap with the previously published dataset. For clear attribution and to distinguish what is novel, the manuscript should state explicitly that the 2017 cohort is previously published. This is essential for transparency and to avoid redundant reporting.

We thank Reviewer #4 for their careful and constructive comments, which prompted clearer presentation, and a more cautious interpretation of the clustering approach and risk factor analyses, substantially improving the manuscript.

Firstly; thank you for highlighting the ambiguity regarding the 2017 cohort and Akoi Bore et al. in our manuscript. We have taken this important point on board and rephrased at multiple places (Introduction – line 46, Results - Table 1, line 73, Materials & Methods – line 429) in the manuscript to ensure this is clearly communicated.

Interpretation of serological cross-reactivity and attribution to filovirus species :
Filoviruses, especially within the Ortho-ebolavirus group, are well recognized to induce broad cross-reactive antibody responses. While the authors acknowledge this, seropositivity based primarily on glycoprotein reactivity is particularly prone to overestimation and misattribution of

species. The repeated interpretation of seropositivity as definitive evidence of exposure to multiple, including highly geographically restricted, filovirus species (e.g., Lloviu virus, so far detected only in European bats, and Reston virus) is not sufficiently justified given these cross-reactivities.

We thank the reviewer for these insightful comments and agree that extensive serological cross-reactivity among ortho-ebolaviruses can lead to overestimation of seroprevalence and misattribution of species when relying primarily on GP reactivity. We have revised the manuscript accordingly to address these limitations more explicitly (line 304).

However, given that GP—the sole viral protein expressed on the virion surface—is the main target of the humoral response in EVD survivors *(3, 37, 41)*, and that anti-GP antibodies decline less rapidly than anti-NP or anti-VP40 antibodies *(42)*, it may be possible that other target antigens may underestimate seroprevalence. Other more highly conserved proteins, such as NP, were also less likely to allow us to differentiate between the filovirus species. Future directions could include inclusion of additional viral proteins (e.g., NP, VP35, VP50) to balance the trade-off between sensitivity and specificity, and this has been included in the discussion (line 408). We have added discussion of these considerations to the discussion based on this valuable feedback.

We also acknowledge the challenges of cross-reactivity, and our revised manuscript attempted to state our claims more cautiously, e.g. line 353: "This may be interpreted cautiously as preliminary evidence for the circulation of an antigenically related cuevavirus in the region (rather than the circulation of the LLOV species, yet to be identified outside of Europe)."

K-means Clustering interpretation

The use of unsupervised K-means clustering to define serological "exposure profiles" in the absence of clear serological cutoffs is innovative, but problematic in its current implementation. The authors describe partitioning the data into 15 clusters; this number seems excessive and potentially not biologically meaningful. The method for selection of the number (e.g., AIC, gap statistic, silhouette width, etc.) should be fully described, with supporting criteria/plots included as supplementary material. L146-154 the manuscript describes eight clusters with apparently interpretable patterns (clusters assigned to EBOV, BDBV, etc.), but provides no information about the remaining clusters nor what constitutes a "negative" cluster. For example, cluster 5 (with high MFI for both BDBV and EBOV) is neither described nor considered. This incomplete reporting undermines the risk factor analyses, which are all based on cluster assignments.

We thank the reviewer for this detailed review of the k-means clustering approach and agree that clearer justification, validation, and more transparent reporting were required. We have substantially revised the manuscript to address these concerns.

As discussed in the revised methods, k-means clustering was employed as an exploratory tool to identify patterns of antibody reactivity in continuous multiplex immunoassay data, to avoid the use of binary seropositivity cut-offs (which would result in substantial misclassification due to cross-reactivity). Clustering was therefore used to group individuals with similar immunological profiles for downstream testing and analysis, and not as a definitive classification of exposure; we have significantly revised the results (line 166), discussion (lines 304 - 312) and methods (line 533-536) to better describe its intended use and acknowledge its limitations.

We have provided additional details to justify the selection of k = 15. The number of clusters (k=15) was determined through multiple complementary validation approaches. First, we visually examined the results of different numbers of clusters and evaluated this using the elbow

method. As we aimed to identify groups of exposures across different pathogens, we only include k > 4. The elbow method demonstrated diminishing returns in within-cluster sum of squares beyond k=15 (after K=15 was less than 21323813075). The gap statistic confirmed that k=15 produced clustering structure substantially better than expected by chance, while silhouette analysis showed adequate cluster cohesion (average silhouette width = 0.23). The Calinski-Harabasz index and Davies-Bouldin index metrics indicated a range of statistically acceptable solutions rather than a single optimum.

Visual inspection across k = 2–25 showed that k = 15 provided the best balance between resolution and over-fragmentation, capturing distinct antigenic patterns across nine filovirus glycoproteins while maintaining coherent cluster structure. Given that this analysis encompasses nine filovirus species (EBOV, BDBV, TAFV, BOMV, SUDV, LLOV, RESTV, MLAV, and RAVV), k=15 provides the resolution for identifying species-specific serological profiles for downstream confirmatory testing, without over-fragmenting the data into statistically indistinguishable subgroups.To address concerns regarding biological meaning, we have added new supplementary figures showing individual-level MFI responses within clusters. These demonstrate that clusters share consistent ordinal antigenic profiles (e.g., EBOV > BDBV > BOMV or BDBV > EBOV), supporting the biological coherence of the clustering solution.

In response to concerns about incomplete reporting, we have revised the Results (Figure 3) to describe and display all clusters (rather than just 8) alongside their biological annotations, in order to improve transparency and demonstrate their biological relevance. Cluster 5 is now described explicitly in the text.

Risk factor analyses are now framed as exploratory and hypothesis-generating, and their limitations are discussed explicitly. We believe these revisions address the reviewer's concerns and substantially strengthen the clarity, transparency, and interpretability of the clustering approach.

Specificity of PV Neutralization Assay:
The specificity of the pseudotyped virus (PV) neutralization assay is unclear. Results from the EBOV survivor pool showed no significant difference between neutralization of EBOV and BDBV (or TAFV), suggesting that the PV neutralization assay itself may be subject to substantial cross-reactivity. Could the authors clarify the assay's specificity and provide evidence for its capacity to discriminate species-specific neutralizing antibodies within the orthobolaviruses?

We agree with the reviewer that pseudotyped virus neutralisation assays (PVNAs) can exhibit cross-reactivity within ortho-ebolaviruses and do not provide definitive species-specific discrimination, as reflected by overlapping neutralisation responses in the EBOV survivor pool. However, parallel neutralisation assays are a technique which have been used as the gold standard for differentiating between highly-cross reactive viral species such as the Japanese Encephalitis Virus group.

We have revised the manuscript to clarify that PVNAs were used as a functional, comparative assay to support serological patterns rather than as a definitive species-level identifier. We state that discrimination of species-specific neutralising responses within ortho-ebolaviruses remains limited without live-virus assays, and this limitation is discussed more explicitly (line 405), and have reworded the Results and Figure 4 legend throughout to explicitly note when confidence intervals overlap to soften claims of species-specific neutralisation (line 180, line 187, line 332).

Additionally, are the glycoprotein antigens used in the pseudotyped virus production identical to those used in the multiplex microsphere-based immunoassay (MMIA)? No, they were not. This has been clarified in additional supplementary tables (Table 3 and Table 4).

PV Neutralization Assay Results interpretation

The authors should be cautious as well with the interpretation of the PV Neutralization Assay Results. The neutralization data are interpreted as supporting species-specific exposures in several cases and authors repeatedly wrote that neutralisation for BDBV (or TAFV) was stronger than for EBOV for select individuals, but in most cases, NT50 confidence intervals overlap (see Figure 4a/b), which is indeed mentioned by the authors for TAFV neutralisation but not for BDBV. What statistical comparisons were performed? Are differences between neutralization curves actually significant? Authors should use appropriate paired statistical tests where possible, and interpret results conservatively when confidence intervals are overlapping. Specific individuals are highlighted as putatively BDBV-exposed, yet displayed overlapping neutralization results (For instance, JAB376 is interpreted by the authors as "Individuals who showed stronger neutralisation of BDBV than EBOV in parallel lentiviral pseudotyped virus neutralized tests" in Figure 7 whereas there is no clear difference in figure 4a). The neutralization assay's resolution should be addressed and limitations made explicit.

Moreover, the PV neutralization data are limited to paired comparisons of a single filovirus (BDBV, TAFV, or LLOV) versus EBOV for each sample. Were these samples also tested for neutralization against the other filoviruses in the panel? If so, it would strengthen the analysis to present comparative neutralization data for everyone across all tested viruses, rather than only in relation to EBOV.

We agree with the reviewer that the original interpretation of the PV neutralisation data was overly strong in places and required more conservative interpretation. We have revised the manuscript to remove statements implying species-specific exposure where NT_{50} confidence intervals overlap, including for BDBV, and now describe such findings more cautiously. In cases where confidence intervals overlapped or statistical support was lacking, this is now stated clearly, and individuals are no longer highlighted as definitively "more strongly" neutralising one virus over another.

We have also revised the text to explicitly address the limited resolution of PV neutralisation assays within ortho-ebolaviruses and to emphasise that these assays provide supportive, functional information rather than definitive species-level discrimination in the absence of live-virus neutralisation, or other alternative methods.

Finally, we agree with the reviewer that a comprehensive neutralisation testing of sera across all filoviruses would be interesting and informative. Unfortunately, neutralisation testing was limited to paired comparisons with EBOV because of constraints in serum volume, assay throughput, and pseudotyped virus availability; comprehensive testing of all samples against all filoviruses in the panel was therefore not feasible. We now state this limitation explicitly and frame the PV neutralisation data as targeted, comparative analyses rather than exhaustive profiling across all filoviruses (line 406).

Hunter/Non-hunter

L355-356 "Further, overall population-level differences could still be seen between hunters and their close contacts, potentially highlighting the additional risks of increased time in the forest or exposure to fresh bodily fluids."

While the title and discussion emphasize wildlife hunting as a driver of exposure, the only reported group comparison is a supplementary figure showing MFI distributions. No odds ratios for hunting status are provided. Most of the analysis is based solely on the BDBV cluster (for which only NHP consumption and male sex are reported as significant predictors). A full table showing all major risk factors (including bushmeat consumption, hunting, sex, age, etc.) and their statistical associations with all filovirus seropositivity clusters should be included, along with clear reporting on sample sizes for each comparison. This is essential both to address the stated research question and to support conclusions regarding risk factors.

The authors at times use “hunter cohorts,” “wildlife hunters,” and “study population” interchangeably. Yet Table 1 makes clear that both hunters and their close contacts (non-hunters) are included, however most figures are described to include “wildlife hunters” but appear to include the entire study population (Figures 1 and 3 include both hunter and non-hunter groups, as per Table 1). It is unclear for Figure 6 “amongst Guinean Wildlife hunters” whether the authors only include mean MFI from hunters in this figure or if it is a misuse? This should be clarified throughout the text and in figure legends/titles, as should any analysis restricted to hunters or resampled individuals.

We thank the reviewer for highlighting a major ambiguity within the study. We agree that the original presentation overstated differences between hunters and non-hunters and that inconsistent terminology contributed to confusion. Wildlife hunters were individuals who actively hunted, and “non-hunters” were individuals who did not hunt themselves, were close contacts of hunters. Based on our questionnaire responses, even “non-hunters” frequently butchered, cooked and consumed wildlife carcasses and therefore had similar exposure to wildlife meat.

In response to this and related reviewer feedback, we have removed explicit hunter versus non-hunter comparisons and associated interpretations from the manuscript, including the statement on lines 355–356. We therefore elected to focus risk factor analyses on more specific behavioural variables (e.g., nonhuman primate consumption) – relevant tables with the ORs have been added (Supplementary, Table 10).

Overall, the title seeks to highlight that we demonstrated increased filovirus reactivity within purposively sampled wildlife hunting communities. Due to this study design (resulting in ubiquitous wildlife meat exposure) we are unable to assess the risk of wildlife hunting, which we have outlined as a major limitation of the study (line 381) in the discussion.

Finally, we acknowledge the inconsistent terminology and have revised the manuscript to distinguish clearly between “wildlife hunters,” “close household contacts,” and the overall “study population i.e., wildlife hunting communities” throughout. Thank you for bringing this to our attention.

Resampling

For resampling: L74 mentions 256 individuals, but only 12 dots are depicted in Figure 5—please clarify how many individuals were longitudinally resampled and are analyzed for antibody durability or new exposure. What is the percentage of resampled individuals which are still considered seropositive ?

We thank the reviewer for raising these points and agree that clearer explanation was required. We have revised the manuscript to explicitly state that although 256 individuals were re-contacted and provided samples in 2023, only a subset met the criteria for inclusion in the longitudinal antibody durability analysis shown in Figure 5 (i.e., individuals who were seropositive for EBOV above the defined threshold in 2017 and had sufficient paired data available) (line 206). The number of individuals included in each longitudinal analysis is now reported in the figure caption (Figure 5).

The authors state they have demonstrated "ongoing seroconversion." Please clarify which results directly support this assertion. Specifically, what is the rate or percentage of seroconversion observed among re-sampled individuals between 2017 and 2023, and how is this determined?

In particular we refer to 2 individuals who demonstrated seroconversion to LLOV or a related virus occurring between 2017 and 2023; we have included an additional Supplementary figure demonstrating the magnitude of the antibody change (Supplementary, Figure 11).

EBOV serology

Authors concluded on L274 that they demonstrated "broad and durable B-cell mediated antibody response" to EBOV following the 2013-2016 outbreak. However, this claim should be more cautiously stated, as the study was not designed as a follow-up of confirmed EBOV survivors but rather sampled a mixed population from several villages. While the presence of reactive antibodies to EBOV glycoprotein is shown, the exact percentage of seropositive individuals within the population is not reported, nor are results stratified according to whether villages were affected or unaffected by the outbreak. Figure 6, which presents MFI values by village, is interesting but authors only commented on BDBV MFI and did not discuss EBOV results. Some villages show high EBOV MFI mean despite being unaffected (e.g., villages 4 and 26 in 2017, and villages 4, 35, 42, and 43 in 2023).

We agree with the reviewer that the original wording overstated the strength of the conclusions regarding EBOV-specific immunity. The study was not designed as a longitudinal follow-up of PCR-confirmed Ebola virus disease survivors, but rather sampled a mixed population across multiple villages, and we have revised the manuscript to reflect this more clearly. Specifically, we have removed or softened language describing a "broad and durable B-cell mediated antibody response" following the 2013–2016 outbreak, and now state more cautiously that EBOV glycoprotein–reactive antibodies were detectable at the population level several years after the outbreak, without inferring confirmed infection or immunity (line 296).

We have included the findings of increased EBOV reactivity in unaffected villages to the results (line 204) and expanded in the discussion on the limitations of our attempts to determine EBOV exposure in villages and individuals (lines 393-398).

Minor comments

Clarify whether "hunter," "wildlife hunter," or "study population" refers to all participants or only a subgroup, in figures, legends, and Results text. Clarified.

In L73 and in Table 1, authors should mention that 2017 cohort was previously published by Akoi

Boré et al., Nature Communications, 2024. Citations have been added.

OR Univariable and multivariable are mentioned by the authors L233, but which OR are in the following sentences (OR of BDBV cluster seropositivity for non-human primate consumption and gender) ? When presenting risk factor odds ratios, specify whether they derive from univariable or multivariable models. Consider providing a supplementary table showing ORs (with 95% CIs) for all tested risk factors and each serological outcome. Supplementary table has been added.

Village numbering
The presentation of village-level data (Figure 6) is unclear: are village numbers consistent between 2017 and 2023 ? (this doesn't seem the case based on the affected/unaffected status). Keeping the same numbers in both panel would help interpretation. Moreover, since village 4 is named Maroua at one point, the sentence "Villages are anonymized" seems unnecessary. Authors can simply mention that villages are numbered.

Villages were numbered according to geographic proximity (longitude, latitude). As additional villages were sampled in 2023, the village numbering system is different between 2017 and 2023. We apologise for any confusion and have revised the manuscript for clarity.

Authors should mention the exact number of samples tested at each step in the analyses:
Line 162–163: "Individuals with antibody profiles consistent with exposure to BDBV, TAFV, BOMV, RESTV and LLOV were then assessed for their ability to neutralize the filovirus..." ; Line 168: "Of those tested from the BDBV cluster..." ; Line 174: "Of the individuals tested from the TAFV cluster..."
Please specify the number of samples from each cluster that were tested in the neutralization assay. Clarified.

Line 190–193: "For individuals with detectable EBOV antibodies in 2017, a comparison of 2017 to 2023 demonstrated the population-level durability of anti-EBOV antibodies across all villages. Antibody decay rates were modeled as an exponential decrease from time of infection, with a regression model fitted to IgG antibody data from each individual above a seropositivity threshold for EBOV in 2017 who was resampled in 2023." Please specify how many were resampled and included in the analyses, in the methods, results and the legends of figure 5. Clarified.

On line 100, the statement "Bat exposure and hunting was reported to be highly seasonal, with 87% (292/334) of respondents seeing them or hunting them (92%, 122/133) at the start of the dry season" is unclear with respect to the denominators for each percentage. Please clarify the exact numbers and corresponding percentages for each response group. An additional table has been added to the supplementary to clarify.

We thank the reviewer for their considered and thoughtful review. We have revised the manuscript in light of the minor points to improve clarity and consistency throughout. Terminology has been standardised to distinguish clearly between wildlife hunters, close household contacts, and the overall study population, and figure titles and legends now specify which participants are included in each analysis. The prior publication of the 2017 cohort (Akoi Boré et al., Nature Communications, 2024) is now stated explicitly, and all reported odds ratios are clearly identified as deriving from

univariable or multivariable models, with a supplementary table (Supplementary, Table 10) provided for the tested risk factors and outcomes.

References

- 1. Roe MD, Hood G, Spencer SL, Yan L, Bore JA, Tipton T, et al. Performance of an envelope glycoprotein-based multiplex immunoassay for Ebola virus antibody detection in a cohort of Ebola virus disease survivors. Journal of virological methods. 2024:115057.
- 2. MacNeil A, Reed Z, Rollin PE. Serologic cross-reactivity of human IgM and IgG antibodies to five species of Ebola virus. PLoS Negl Trop Dis. 2011;5(6):e1175–e.
- 3. Flyak AI, Shen X, Murin CD, Turner HL, David JA, Fusco ML, et al. Cross-reactive and potent neutralizing antibody responses in human survivors of natural ebolavirus infection. Cell. 2016;164(3):392–405.
- 4. Ferrara F, Temperton N. Pseudotype Neutralization Assays: From Laboratory Bench to Data Analysis. Methods Protoc. 2018;1(1).
- 5. Steeds K, Hall Y, Slack GS, Longet S, Strecker T, Fehling SK, et al. Pseudotyping of VSV with Ebola virus glycoprotein is superior to HIV-1 for the assessment of neutralising antibodies. Scientific reports. 2020;10(1):14289.
- 6. Ashton RA, Kefyalew T, Rand A, Sime H, Assefa A, Mekasha A, et al. Geostatistical modeling of malaria endemicity using serological indicators of exposure collected through school surveys. Am J Trop Med Hyg. 2015;93(1):168–77.
- 7. White MT, Baudemont G, Donnadieu F, Diatta AS, Sarr I, Diagne A, et al. Whose line is it anyway? Defining sero-positivity cut-offs for infectious disease surveillance. J Infect Dis. 2025.
- 8. Chan Y, Fornace K, Wu L, Arnold BF, Priest JW, Martin DL, et al. Determining seropositivity—A review of approaches to define population seroprevalence when using multiplex bead assays to assess burden of tropical diseases. PLOS Neglected Tropical Diseases. 2021;15(6):e0009457.
- 9. Timothy JW, Hall Y, Akoi-Boré J, Diallo B, Tipton TR, Bower H, et al. Early transmission and case fatality of Ebola virus at the index site of the 2013–16 west African Ebola outbreak: a cross-sectional seroprevalence survey. The Lancet Infectious Diseases. 2019;19(4):429–38.
- 10. Hounmenou CG, Marcis FL, Kaba D, Diaby M, Soumah AK, Diallo H, et al. Ebola virus circulation in a non-epidemic Guinean rural area: A mixed-method approach to assessing endemicity. International journal of infectious diseases : IJID : official publication of the International Society for Infectious Diseases. 2024;146:107129.
- 11. Manno D, Ayieko P, Ishola D, Afolabi MO, Rogers B, Baiden F, et al. Ebola Virus Glycoprotein IgG Seroprevalence in Community Previously Affected by Ebola, Sierra Leone. Emerg Infect Dis. 2022;28(3):734–8.
- 12. Surtees R, Stern D, Ahrens K, Kromarek N, Lander A, Kreher P, et al. Development of a multiplex microsphere immunoassay for the detection of antibodies against highly pathogenic viruses in human and animal serum samples. PLoS Neglected Tropical Diseases. 2020;14(10):e0008699.
- 13. Clark DV, Kibuuka H, Millard M, Wakabi S, Lukwago L, Taylor A, et al. Long-term sequelae after Ebola virus disease in Bundibugyo, Uganda: a retrospective cohort study. Lancet Infect Dis. 2015;15(8):905–12.
- 14. World Health Organization. Ebola haemorrhagic fever in Zaire, 1976. Bull World Health Organ. 1978;56(2):271–93.
- 15. Glynn JR, Bower H, Johnson S, Houlihan CF, Montesano C, Scott JT, et al. Asymptomatic infection and unrecognised Ebola virus disease in Ebola-affected households in Sierra

Leone: a cross-sectional study using a new non-invasive assay for antibodies to Ebola virus. The Lancet infectious diseases. 2017;17(6):645–53.

- 16. Rowe AK, Bertolli J, Khan AS, Mukunu R, Muyembe-Tamfum J, Bressler D, et al. Clinical, virologic, and immunologic follow-up of convalescent Ebola hemorrhagic fever patients and their household contacts, Kikwit, Democratic Republic of the Congo. The Journal of infectious diseases. 1999;179(Supplement_1):S28–S35.
- 17. Gayedyu-Dennis D, Fallah MP, Drew C, Badio M, Moses JS, Fayiah T, et al. Identifying Paucisymptomatic or Asymptomatic and Unrecognized Ebola Virus Disease Among Close Contacts Based on Exposure Risk Assessments and Screening Algorithms. J Infect Dis. 2023;227(7):878–87.
- 18. Richardson ET, Kelly JD, Barrie MB, Mesman AW, Karku S, Quiwa K, et al. Minimally symptomatic infection in an Ebola 'hotspot': a cross-sectional serosurvey. PLoS neglected tropical diseases. 2016;10(11):e0005087.
- 19. Bertherat E, Renaut A, Nabias R, Dubreuil G, Georges-Courbot MC. Leptospirosis and Ebola virus infection in five gold-panning villages in northeastern Gabon. Am J Trop Med Hyg. 1999;60(4):610–5.
- 20. Leroy EM, Baize S, Volchkov VE, Fisher-Hoch SP, Georges-Courbot MC, Lansoud-Soukate J, et al. Human asymptomatic Ebola infection and strong inflammatory response. Lancet. 2000;355(9222):2210–5.
- 21. Heffernan RT, Pambo B, Hatchett RJ, Leman PA, Swanepoel R, Ryder RW. Low seroprevalence of IgG antibodies to Ebola virus in an epidemic zone: Ogooue-Ivindo region, Northeastern Gabon, 1997. The Journal of infectious diseases. 2005;191(6):964–8.
- 22. Dean NE, Halloran ME, Yang Y, Longini IM. Transmissibility and pathogenicity of Ebola virus: a systematic review and meta-analysis of household secondary attack rate and asymptomatic infection. Clinical Infectious Diseases. 2016;62(10):1277–86.
- 23. Knobloch J, Albiez EJ, Schmitz H. A serological survey on viral haemorrhagic fevers in liberia. Annales de l'Institut Pasteur / Virologie. 1982;133(2):125–8.
- 24. Van der Waals FW, Pomeroy K, Goudsmit J, Asher D, Gajdusek D. Hemorrhagic fever virus infections in an isolated rainforest area of central Liberia. Limitations of the indirect immunofluorescence slide test for antibody screening in Africa. Tropical and geographical medicine. 1986;38(3):209–14.
- 25. Tomori O, Fabiyi A, Sorungbe A, Smith A, McCormick JB. Viral hemorrhagic fever antibodies in Nigerian populations. The American journal of tropical medicine and hygiene. 1988;38(2):407–10.
- 26. Keita AK, Butel C, Thaurignac G, Diallo A, Nioke T, Traoré F, et al. Serological Evidence of Ebola Virus Infection in Rural Guinea before the 2014 West African Epidemic Outbreak. Am J Trop Med Hyg. 2018;99(2):425–7.
- 27. Bane S, Rosenke K, Maiga O, Feldmann F, Meade-White K, Callison J, et al. Ebola Virus IgG Seroprevalence in Southern Mali. Emerging Infectious Diseases. 2021;27(6):1681.
- 28. Ramassamy J-L, Ayouba A, Thaurignac G, Bilounga Ndongo C, Nnuka P, Betsem E, et al. High Seroreactivities to Orthoebolaviruses in Rural Cameroon: A Case-Control Study on Nonhuman Primate Bites and a Cross-sectional Survey in Rural Populations. The Journal of Infectious Diseases. 2024;230(5):e1067–e76.
- 29. Steffen I, Lu K, Yamamoto LK, Hoff NA, Mulembakani P, Wemakoy EO, et al. Serologic prevalence of Ebola virus in equatorial Africa. Emerging infectious diseases. 2019;25(5):911.
- 30. Schoepp RJ, Rossi CA, Khan SH, Goba A, Fair JN. Undiagnosed acute viral febrile illnesses, Sierra Leone. Emerging infectious diseases. 2014;20(7):1176.
- 31. Safronetz D, Sacko M, Sogoba N, Rosenke K, Martellaro C, Traore S, et al. Vectorborne infections, Mali. Emerging Infectious Diseases. 2016;22(2):340.
- 32. Boisen ML, Schieffelin JS, Goba A, Oottamasathien D, Jones AB, Shaffer JG, et al. Multiple circulating infections can mimic the early stages of viral hemorrhagic fevers and possible

human exposure to filoviruses in Sierra Leone prior to the 2014 outbreak. Viral immunology. 2015;28(1):19–31.

- 33. Dedkov VG, Magassouba NF, Stukolova OA, Savina VA, Camara J, Soropogui B, et al. Differential laboratory diagnosis of acute fever in guinea: preparedness for the threat of hemorrhagic fevers. International journal of environmental research and public health. 2021;18(11):6022.
- 34. Kelly JD, Barrie MB, Mesman AW, Karku S, Quiwa K, Drasher M, et al. Anatomy of a hotspot: chain and seroepidemiology of Ebola virus transmission, Sukudu, Sierra Leone, 2015–16. The Journal of infectious diseases. 2018;217(8):1214–21.
- 35. Houlihan CF, McGowan CR, Dicks S, Baguelin M, Moore DA, Mabey D, et al. Ebola exposure, illness experience, and Ebola antibody prevalence in international responders to the West African Ebola epidemic 2014–2016: A cross-sectional study. PLoS medicine. 2017;14(5):e1002300.
- 36. Kelly JD, Van Ryn C, Badio M, Fayiah T, Johnson K, Gayedyu-Dennis D, et al. Clinical sequelae among individuals with pauci-symptomatic or asymptomatic Ebola virus infection and unrecognised Ebola virus disease in Liberia: a longitudinal cohort study. The Lancet Infectious Diseases. 2022;22(8):1163–71.
- 37. Diallo MSK, Rabilloud M, Ayouba A, Touré A, Thaurignac G, Keita AK, et al. Prevalence of infection among asymptomatic and paucisymptomatic contact persons exposed to Ebola virus in Guinea: a retrospective, cross-sectional observational study. The Lancet Infectious Diseases. 2019;19(3):308–16.
- 38. Kelly JD, Frankfurter RG, Tavs JM, Barrie MB, McGinnis T, Kamara M, et al., editors. Association of lower exposure risk with paucisymptomatic/asymptomatic infection, less severe disease, and unrecognized Ebola virus disease: a seroepidemiological study. Open forum infectious diseases; 2022: Oxford University Press US.
- 39. Halfmann PJ, Eisfeld AJ, Watanabe T, Maemura T, Yamashita M, Fukuyama S, et al. Serological analysis of Ebola virus survivors and close contacts in Sierra Leone: A cross-sectional study. PLoS Neglected Tropical Diseases. 2019;13(8):e0007654.
- 40. Longet S, Leggio C, Bore JA, Key S, Tipton T, Hall Y, et al. Influence of Landscape Patterns on Exposure to Lassa Fever Virus, Guinea. Emerg Infect Dis. 2023;29(2):304–13.
- 41. Becquart P, Mahlakõiv T, Nkoghe D, Leroy EM. Identification of Continuous Human B-Cell Epitopes in the VP35, VP40, Nucleoprotein and Glycoprotein of Ebola Virus. PLOS ONE. 2014;9(6):e96360.
- 42. Diallo MSK, Ayouba A, Thaurignac G, Sow MS, Kpamou C, Barry TA, et al. Temporal evolution of the humoral antibody response after Ebola virus disease in Guinea: a 60-month observational prospective cohort study. The Lancet Microbe. 2021;2(12):e676–e84.
